# RNF31 restricts EV-A71 replication through innate immune activation and VP4 degradation, and is antagonized by viral 3C proteases

**DOI:** 10.1371/journal.ppat.1014415

**Published:** 2026-07-02

**Authors:** Qingxiang Zhang, Yuan Gao, Wenying Gao, Xue Zhang, Shengyuan Xu, Wenyan Zhang

**Affiliations:** 1 Institute of Virology and AIDS Research, Centre of Infectious Diseases and Pathogen Biology, Key Laboratory of Organ Regeneration and Transplantation of the Ministry of Education, the First Hospital of Jilin University, Changchun, China; 2 Jilin Provincial Key Laboratory on Molecular and Chemical Genetics, the Second Hospital of Jilin University, Changchun, China; National University of Singapore, SINGAPORE

## Abstract

A persistent evolutionary arms race exists between enteroviruses and their hosts, in which viruses employ multiple strategies to antagonize host antiviral defenses and sustain efficient replication. However, how host restriction factors are broadly targeted by enteroviruses during this process, as well as the underlying molecular mechanisms, remain poorly understood. Here, we identify ring finger protein 31 (RNF31) as a previously unrecognized host restriction factor that limits enterovirus A71 (EV-A71) replication through a dual antiviral mechanism. Specifically, RNF31 enhances innate antiviral immune signaling by promoting K63-linked polyubiquitination of Retinoic acid-inducible gene I (RIG-I). Simultaneously, RNF31 directly suppresses EV-A71 replication by inducing K27- and K48-linked polyubiquitination of the Viral Protein 4 (VP4), thereby resulting in its proteasome-dependent degradation. Furthermore, we demonstrate that the viral 3C protease (3Cpro) cleaves RNF31 at residue Q400, abolishing both RNF31-mediated activation of innate immunity and VP4 degradation, ultimately resulting in the loss of its antiviral activity. Notably, 3Cpro from multiple enteroviruses, including Coxsackievirus A16 (CV-A16), Coxsackievirus B3 (CV-B3), and Enterovirus D68 (EV-D68), cleave RNF31 at this same conserved site. Consistent with these findings, RNF31 significantly inhibits the replication of these diverse enteroviruses. Collectively, this study establishes RNF31 as a host restriction factor that suppresses the replication of multiple enteroviruses and reveals a shared immune evasion strategy employed by enteroviruses.

## Author summary

Enteroviruses must overcome host antiviral defenses to replicate efficiently, but how they broadly counteract host restriction factors is not fully understood. Here, we identify RNF31 as a host protein that inhibits the replication of multiple enteroviruses. RNF31 acts in two ways: it enhances innate antiviral immune signaling and directly promotes the degradation of the viral protein VP4. However, enteroviruses evade this defense by using their 3C protease to cleave RNF31, thereby disabling its antiviral functions. This cleavage mechanism is conserved among several enteroviruses. Our findings reveal RNF31 as a broad antiviral factor and uncover a shared strategy used by enteroviruses to escape host immunity.

## Introduction

Enterovirus A71 (EV-A71), a member of the genus *Enterovirus* within the family *Picornaviridae*, is a major causative agent of hand, foot, and mouth disease (HFMD) [1,2]. As a neurotropic enterovirus of significant clinical importance, EV-A71 infection typically presents with mild symptoms, including fever, oral herpangina, and rashes on the hands and feet. However, a subset of cases can progress to severe neurological complications, such as meningitis, brainstem encephalitis, acute flaccid paralysis, and neurogenic pulmonary edema [3,4]. EV-A71 was first isolated in 1969 from a patient with central nervous system infection in California, USA [5]. Since the 1990s, EV-A71 has caused multiple large-scale HFMD outbreaks in the Asia-Pacific region, posing a serious threat to infant and child health [6].

EV-A71 enters host cells primarily through receptor-mediated endocytosis. Subsequent endosomal acidification induces conformational changes in the viral capsid, leading to the release of the positive-sense single-stranded RNA genome into the cytoplasm [7,8]. The genomic RNA functions directly as mRNA and is translated into a single large polyprotein, which is subsequently cleaved by the viral 2A protease (2Apro) and 3C protease (3Cpro) to generate four structural proteins (VP1, VP2, VP3, and VP4) and seven non-structural proteins (2A, 2B, 2C, 3A, 3B, 3C, and 3D) [9]. The structural proteins VP1-VP3 are located on the virion surface or within the capsid scaffold and are involved in receptor binding, antigenicity, tissue tropism, capsid stability, and the regulation of viral assembly and uncoating [10,11]. In contrast, VP4 is a small, highly conserved, and hydrophobic protein located on the inner surface of the capsid, where it plays a critical role in viral entry and genome release. Upon endosomal acidification, VP4 is extruded from the capsid interior and inserts into the host membrane, forming a transient pore that enables the translocation of viral RNA into the cytoplasm [12]. This membrane-permeabilizing activity of VP4 is a key determinant of viral uncoating efficiency and represents a critical rate-limiting step in the EV-A71 life cycle [11]. The non-structural proteins assemble on rearranged intracellular membrane structures to form viral replication complexes, where new positive-strand genomic RNA is synthesized via a negative-strand RNA intermediate. Newly synthesized positive-strand RNA molecules subsequently associate with structural proteins in the cytoplasm to form progeny capsids. Viral maturation is completed by the proteolytic cleavage of VP0, yielding infectious virions. Mature viral particles are then released predominantly through cell lysis [13,14].

The 3Cpro of EV-A71 is a highly conserved cysteine protease that plays a central regulatory role in the viral life cycle. In addition to its role in polyprotein processing to generate mature structural and non-structural proteins essential for viral replication and assembly, 3Cpro targets and cleaves multiple host restriction factors, including NOD-like receptor family, pyrin domain-containing 3 (NLRP3), Promyelocytic leukemia protein isoform III (PMLIII), Promyelocytic leukemia protein isoform IV (PMLIV), Heterogeneous nuclear ribonucleoprotein A1 (hnRNP A1), 2′-5′-Oligoadenylate synthetase 3 (OAS3), Interferon (IFN) regulatory factor 7 (IRF7), and Transforming growth factor β-activated kinase 1 (TAK1), thus facilitating viral immune evasion and sustained replication [15–20]. Structural biology studies have elucidated the catalytic triad and substrate-binding pocket of EV-A71 3Cpro, providing a strong theoretical framework for the rational design of highly selective small-molecule inhibitors and broad-spectrum anti-enteroviral therapeutics [21,22].

EV-A71 infection activates RIG-I-like receptor (RLR) signaling, leading to the induction of type I IFN responses and the establishment of an antiviral defense in host cells [23–25]. Notably, the initiation and fine-tuning of innate immune signaling pathways, including RLR-mediated antiviral responses, are tightly regulated by post-translational modifications [26]. Ubiquitination is a fundamental post-translational modification that plays a critical role in maintaining cellular homeostasis [27]. Through the formation of polyubiquitin chains with distinct linkage types-including K6, K11, K27, K29, K33, K48, K63, and linear (M1-linked) chains-ubiquitination precisely regulates a wide range of biological processes, such as protein degradation, DNA repair, signal transduction, and membrane trafficking [28,29].

Ring finger protein 31 (RNF31) is also known as HOIL-1-interacting protein (HOIP), and contains multiple functional domains, including a RANBP2-type zinc finger (ZNF-RBZ) domain, a ubiquitin-associated (UBA) domain, and a RING-in-between-RING (RBR) domain [30]. The RBR domain confers its E3 ubiquitin ligase activity. Together with RanBP-type and C3HC4-type zinc finger containing 1 (RBCK1; also known as HOIL-1) and SHANK-associated RH domain-interacting protein (SHARPIN), RNF31 forms the linear ubiquitin chain assembly complex (LUBAC) [31]. LUBAC catalyzes the formation of linear polyubiquitin chains, stabilizes signaling complexes, and promotes the activation of NF-κB, IFN, and anti-apoptotic signaling pathways, thereby playing a central role in immune responses, inflammatory regulation, and cell survival [32–35]. In addition, RNF31 modulates the function of substrate proteins by regulating their stability. For example, RNF31 promotes K48-linked ubiquitination and subsequent proteasomal degradation of the transcriptional coactivator YAP, leading to the suppression of PD-L1 expression and tumor immune evasion [36]. Moreover, RNF31 mediates K48-linked ubiquitination of inhibitor of nuclear factor κB kinase subunit alpha (IKKα) and facilitates its proteasomal degradation, which in turn activates NF-κB signaling, enhances the expression of pro-inflammatory factors, and promotes M1 macrophage polarization [37]. Although RNF31 has been reported to enhance antiviral immune responses, its role in modulating EV-A71 replication via innate immunity and its potential function as an E3 ubiquitin ligase in direct antiviral defense remain unexplored [38].

In this study, RNF31 was identified as a host restriction factor during EV-A71 infection. Mechanistic analyses revealed that RNF31 suppresses EV-A71 replication through two distinct pathways. First, RNF31 promotes K63-linked ubiquitination of RIG-I, thereby enhancing RLR-mediated innate immune signaling. Second, RNF31 induces the proteasomal degradation of the viral structural protein VP4 by mediating its K27- and K48-linked polyubiquitination. Notably, this VP4-targeted degradation mechanism is conserved among multiple enteroviruses, including Coxsackievirus A16 (CV-A16), Coxsackievirus B3 (CV-B3), and Enterovirus D68 (EV-D68). In response, enteroviruses have evolved an immune evasion strategy in which the viral 3Cpro specifically cleaves RNF31, thereby disrupting RNF31-mediated antiviral functions and facilitating viral replication. Collectively, these findings elucidate the antiviral role of RNF31 during enterovirus infection and reveal a corresponding viral immune evasion mechanism, providing a conceptual framework for the development of potential therapeutic strategies against enterovirus infections.

## Results

### Identification of RNF31 as a substrate protein of the EV-A71 3Cpro

The 3Cpro encoded by EV-A71 is a key viral protein that not only mediates cleavage and maturation of the viral polyprotein but also participates in the assembly of membrane structures required for viral replication [39]. Increasing evidence indicates that 3Cpro maintains a dynamic balance between viral replication and host immune suppression by directly cleaving or functionally remodeling host proteins [40]. On this basis, the present study employed an LC-MS/MS-based proteomic strategy to systematically identify potential host cleavage substrates of EV-A71 3Cpro. Because proteolytic cleavage of interacting proteins by 3Cpro frequently leads to their destabilization or degradation, co-IP assays were performed using WT 3Cpro or its catalytic site mutant 3Cpro^H40A,C147A^ as bait proteins (S1A Fig). Candidate substrates were identified by comparing proteins differentially enriched between the two co-IP groups. Immunoblotting (IB) analysis confirmed robust and comparable expression of WT 3Cpro and 3Cpro^H40A,C147A^ in transfected cells (S1B Fig). LC-MS/MS analysis identified 60 proteins significantly enriched in WT 3Cpro precipitates relative to the empty vector control and 66 proteins significantly enriched in 3Cpro^H40A,C147A^ precipitates relative to WT 3Cpro. Among these, five proteins RNF31, AGL, TNRC6C, WRNIP1, and ZFR were detected in both groups but exhibited significantly higher enrichment in the 3C-DM samples than in the WT 3Cpro samples (S1C Fig), suggesting that they may represent putative substrates of EV-A71 3Cpro.

**Fig 1:**
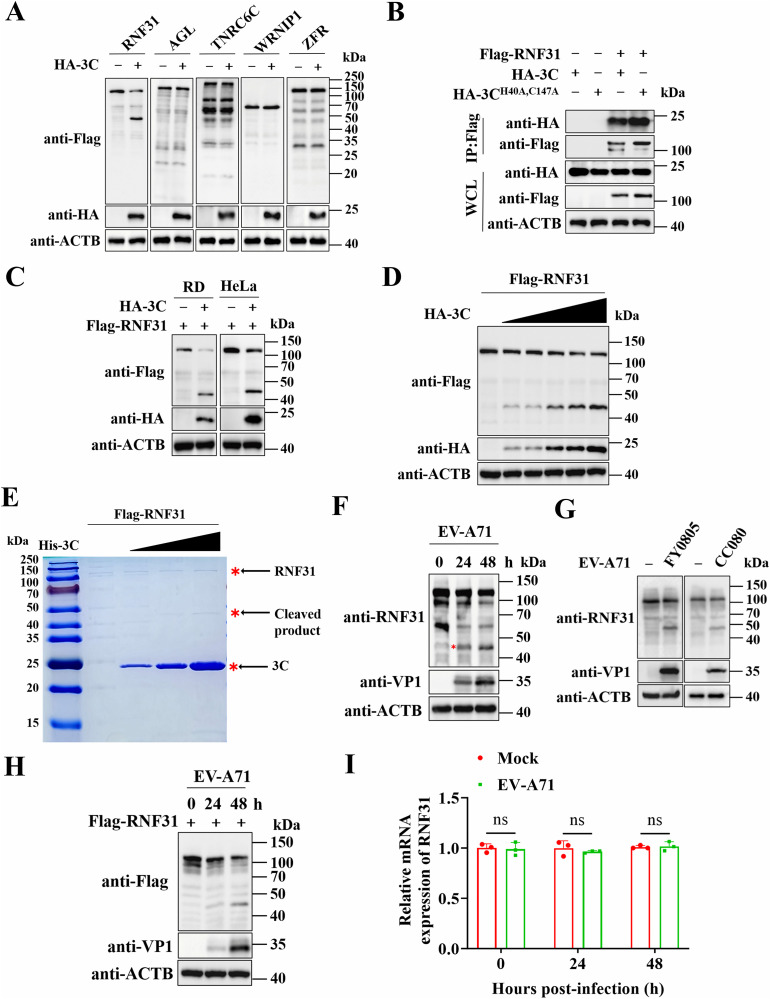
Identification of RNF31 as a substrate protein of EV-A71 3Cpro. **(A)** HEK293T cells were co-transfected with a plasmid encoding a Flag-tagged host protein, along with either the HA-EV-A71 3C plasmid or a control plasmid. After 24 h, cell lysates were analyzed by IB. **(B)** HEK293T cells were co-transfected with Flag-RNF31 and either HA-3C or the catalytically inactive mutant HA-3C^H40A,C147A^. After 48 h, cell lysates were subjected to co-IP using an anti-Flag antibody, followed by IB analysis. **(C)** RD and HeLa cells were transfected with Flag-RNF31 together with HA-3C or a control plasmid, and protein expression was analyzed by IB at 24 h post-transfection. **(D)** HEK293T cells were co-transfected with Flag-RNF31 and increasing amounts of HA-3C. Cell lysates were harvested 24 h later and analyzed by IB. **(E)** Purified His-3C and RNF31 proteins were incubated in an in vitro cleavage buffer at 30°C for 2 **h.** The reaction products were resolved by SDS-PAGE and visualized by Coomassie Brilliant Blue staining. **(F)** HEK293T cells were infected with EV-A71, and endogenous RNF31 protein levels were examined by IB at 24 and 48 h post-infection. **(G)** HEK293T cells were infected with EV-A71-CC080 and EV-A71-FY0805, endogenous RNF31 protein levels were examined by IB at 48 h post-infection. **(H)** HEK293T cells were transfected with Flag-RNF31 for 24 h prior to EV-A71 infection, and cell lysates were analyzed by IB at 24 and 48 h post-infection. **(I)** RNF31 mRNA levels in EV-A71-infected HEK293T cells were quantified by RT-qPCR; ns indicates no statistically significant difference.

Subsequent validation by IB demonstrated that only RNF31 underwent pronounced cleavage upon 3Cpro expression (Fig 1A). Co-IP assays further confirmed that RNF31 interacts with both WT 3Cpro and 3Cpro^H40A,C147A^ (Fig 1B). Consistently, confocal microscopy revealed clear cytoplasmic colocalization of RNF31 with both WT 3Cpro and 3Cpro^H40A,C147A^ (S1D Fig). To determine whether this cleavage event is cell type-specific, additional validation was performed in RD and HeLa cells, in which comparable and robust RNF31 cleavage was also observed (Fig 1C). Moreover, increasing expression levels of 3Cpro resulted in a corresponding enhancement of RNF31 cleavage, indicating a dose-dependent effect (Fig 1D). To further assess whether 3Cpro directly cleaves RNF31, purified recombinant 3Cpro and RNF31 proteins were incubated in vitro, with RNF31 exposed to increasing concentrations of 3Cpro. As the concentration of 3Cpro increased, a progressively stronger cleavage product appeared below the full-length RNF31 band. These results provide direct evidence that RNF31 is cleaved by 3Cpro (Fig 1E).

To determine whether endogenous RNF31 cleavage occurs during EV-A71 infection, we analyzed changes in RNF31 expression in EV-A71 infected HEK293T cells. The results showed that the abundance of full-length RNF31 protein decreased as the duration of viral infection increased, concomitant with the appearance of an approximately 45 kDa fragment. This fragment was detected using an antibody specific to the N-terminal region of RNF31, suggesting that EV-A71 infection induces proteolytic cleavage of RNF31 (Fig 1F). In addition, infection of HEK293T cells with two additional EV-A71 strains produced the same cleavage pattern (Fig 1G). Consistently, similar cleavage fragments were observed in EV-A71-infected cells overexpressing RNF31 (Fig 1H), further supporting the notion that RNF31 is cleavaged during viral infection. Notably, RNF31 mRNA levels remained unchanged following EV-A71 infection (Fig 1I), indicating that the observed reduction in RNF31 protein abundance was not attributable to transcriptional downregulation.

### EV-A71 3Cpro cleaves RNF31 at the Q400 Site

**Fig 2:**
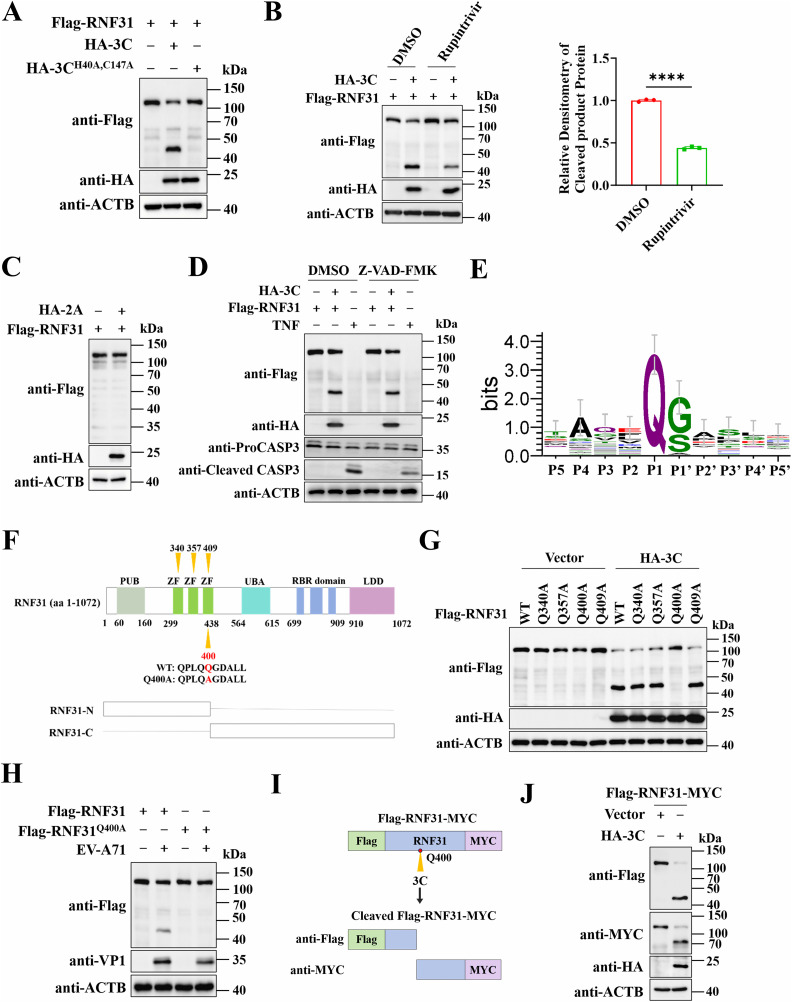
EV-A71 3Cpro cleaves RNF31 at the Q400 Site. **(A)** HEK293T cells were co-transfected with Flag-RNF31 and either HA-3C or HA-3C^H40A,C147A^. Cell lysates were collected 24 h post-transfection and analyzed by IB. **(B)** HEK293T cells co-transfected with Flag-RNF31 and HA-3C were treated with the 3C protease inhibitor rupintrivir (10 μM) or DMSO for 12 h at 24 h post-transfection, followed by IB analysis. Signal intensities were quantified and normalized to the non-cleaved state based on three independent experiments. **(C)** HEK293T cells were transfected with plasmids encoding Flag-RNF31 and HA-2A. Cell lysates were harvested 24 h later and subjected to IB analysis. **(D)** HEK293T cells co-transfected with Flag-RNF31 and HA-3C were treated with the pan-caspase inhibitor Z-VAD-FMK (20 μM), and cell lysates were analyzed by IB. TNF-α (50 ng/mL) was used as a positive control to confirm the inhibitory effect of Z-VAD-FMK on caspase-3 (CASP3) activation. **(E)** Sequence features of the EV-A71 3Cpro cleavage site within the substrate protein. **(F)** Schematic representation of RNF31 domain architecture, with predicted EV-A71 3Cpro cleavage sites indicated. **(G)** HEK293T cells were co-transfected with HA-3C and Flag-RNF31 or its predicted cleavage-site mutants, and cell lysates were analyzed by IB 24 h post-transfection. **(H)** HEK293T cells transfected with Flag-RNF31 or the Flag-RNF31^Q400A^ mutant were infected with EV-A71 at 24 h post-transfection. Cell lysates were collected 48 h post-infection and analyzed by IB. **(I)** Schematic illustration of EV-A71 3C mediated cleavage of Flag-RNF31-MYC. **(J)** HEK293T cells were transfected with Flag-RNF31-MYC together with HA-3C or an empty control plasmid, and cell lysates were analyzed by IB at 24 h post-transfection. Statistical significance is indicated as follows: *P < 0.05; **P < 0.01; ***P < 0.001; ****P < 0.0001; ns, not significant.

To assess whether 3Cpro-mediated cleavage of RNF31 depends on its proteolytic activity, HEK293T cells were co-transfected with Flag-tagged RNF31 and either WT 3Cpro or a catalytically inactive mutant 3Cpro^H40A,C147A^. IB analysis revealed that robust RNF31 cleavage was observed exclusively in cells expressing WT 3Cpro, whereas RNF31 remained intact in cells transfected with the inactive mutant, with no detectable cleavage products (Fig 2A). These results indicate that the protease activity of 3Cpro is essential for RNF31 cleavage. To further confirm the dependence of this process on 3Cpro enzymatic activity, cells were treated with the EV-A71 protease inhibitor rupintrivir [41]. As expected, rupintrivir treatment markedly reduced the efficiency of 3Cpro mediated RNF31 cleavage (Fig 2B). Since EV-A71 encodes another viral protease, 2Apro, in addition to 3Cpro, we next examined whether 2Apro contributes to RNF31 cleavage. Co-expression of Flag-RNF31 with HA-tagged 2Apro did not result in the appearance of RNF31 cleavage products, indicating that 2Apro does not mediate RNF31 cleavage (Fig 2C). To investigate whether RNF31 cleavage is dependent on caspase activity, Flag-RNF31 and HA-3Cpro were co-expressed in HEK293T cells in the presence or absence of the pan-caspase inhibitor Z-VAD-FMK. Treatment with Z-VAD-FMK did not attenuate 3Cpro-mediated RNF31 cleavage. In contrast, under the same conditions, tumor necrosis factor-α (TNF-α)-induced activation of caspase-3 (CASP3), used as a positive control, was effectively inhibited (Fig 2D). Together, these results demonstrate that 3Cpro induced cleavage of RNF31 occurs in a caspase-independent manner.

**Fig 3:**
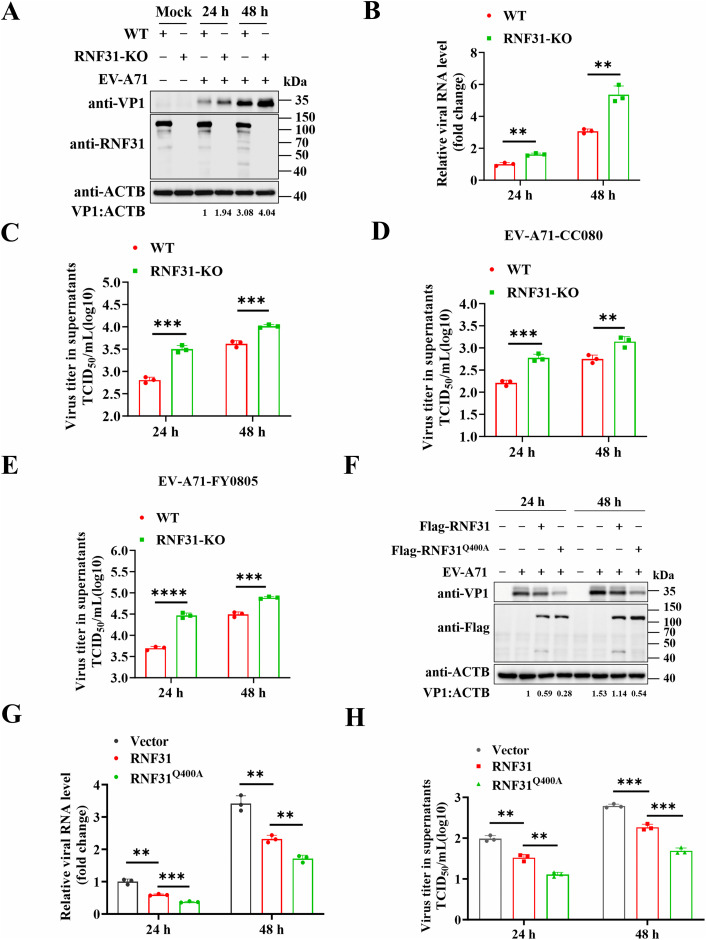
RNF31 inhibits EV-A71 replication in RD cells. **(A-C)** WT and RNF31-KO RD cells were infected with EV-A71 for 24 h and 48 **h.** Following infection, cell lysates were analyzed by IB **(A)**, while culture supernatants were subjected to RT-qPCR (B) and TCID₅₀ assays **(C)**. **(D and E)** WT and RNF31-KO RD cells were infected with EV-A71(CC080 or FY0805) for 24 h and 48 **h.** Following infection, culture supernatants were subjected to TCID₅₀ assays. **(F-H)** RD cells transfected with Flag-RNF31, Flag-RNF31^Q400A^, or control plasmids were infected with EV-A71 for 24 h and 48 **h.** After infection, cell lysates were analyzed by IB **(F)**, and supernatants were collected for RT-qPCR (G) and TCID₅₀ assays **(H)**. Data are presented as the mean ± SEM from three independent experiments. Statistical significance is indicated as follows: *P < 0.05; **P < 0.01; ***P < 0.001; ****P < 0.0001; ns, not significant.

We next performed a systematic analysis of residues flanking known cleavage sites (P5-P5′) in both the EV-A71 polyprotein processed by the 3Cpro and previously reported host protein substrates (S3 Table). As shown in Figs 2E, 3Cpro exhibited clear substrate specificity, with a strong preference for cleavage at the Q(P1)-G/S(P1′) peptide bond, in which a glutamine residue occupies the P1 position. Given that the N-terminal RNF31 cleavage fragment generated by 3Cpro was approximately 45 kDa, four glutamine residues Q340, Q357, Q400, and Q409 were identified as potential cleavage sites within RNF31 (Fig 2F). To precisely map the 3Cpro mediated cleavage site, four RNF31 point mutants (Q340A, Q357A, Q400A, and Q409A) were generated by individually substituting the candidate glutamine residues with alanine. As shown in Fig 2G, Compared with WT RNF31, the Q400A mutant displayed marked resistance to 3Cpro mediated proteolysis, whereas the Q340A, Q357A, and Q409A mutants remained susceptible to cleavage. To further validate this finding under viral infection conditions, Flag-tagged RNF31^Q400A^ was transfected into HEK293T cells followed by EV-A71 infection. No cleavage products were detected in cells expressing RNF31^Q400A^, whereas a distinct cleavage band was readily observed in cells expressing WT RNF31 (Fig 2H). These results indicate that EV-A71 3Cpro specifically cleaves RNF31 at glutamine 400 (Q400). To further confirm the precise cleavage site, a dual-tagged RNF31 construct (Flag-RNF31-MYC) was generated and co-transfected with 3Cpro into HEK293T cells. IB analysis revealed N-terminal and C-terminal cleavage fragments detected by anti-Flag and anti-MYC antibodies, respectively, with molecular weights of approximately 45 kDa and 75 kDa (Fig 2I and 2J). These fragment sizes are consistent with the predicted cleavage products resulting from cleavage at Q400. Collectively, these data demonstrate that 3Cpro cleaves RNF31 specifically at the Q400 residue.

### RNF31 inhibits EV-A71 replication

The specific cleavage of RNF31 by EV-A71 3Cpro suggests that RNF31 may function as a host inhibitory factor during viral replication, thereby driving the evolution of viral antagonistic mechanisms targeting this protein. Based on this hypothesis, an RNF31-knockout (RNF31-KO) RD cell line was generated using CRISPR-Cas9 technology. Sanger sequencing revealed the insertion of a single guanine (G) nucleotide in the first exon of the RNF31 gene, resulting in a frameshift mutation (S2A and S2B Fig). IB analysis further confirmed the complete absence of RNF31 protein expression in RNF31-KO cells (S2C Fig). In parallel, CCK-8 assays demonstrated that RNF31 deficiency did not significantly affect cell viability (S2D Fig). EV-A71 replication was subsequently compared between RNF31-WT and RNF31-KO RD cells. At 24 h and 48 h post-infection, viral titers and viral protein levels were significantly elevated in RNF31-KO cells relative to control cells (Fig 3A-C). Consistently, RNF31 knockout also elevated the viral titers of two additional EV-A71 strains (Fig 3D and 3E), indicating that RNF31 restricts EV-A71 replication. Consistent with these observations, ectopic expression of RNF31 in RD cells markedly reduced viral titers and viral protein expression at both 24 h and 48 h post-infection (Fig 3F-H), further supporting the antiviral role of RNF31. Notably, compared with RNF31-WT, the cleavage-resistant RNF31^Q400A^ mutant exhibited a more pronounced antiviral effect (Fig 3F-H), providing additional evidence that RNF31 acts as a critical host restriction factor during EV-A71 infection. To assess the generalizability of these findings, parallel experiments were performed in HEK293T cells. Consistent with the results obtained in RD cells, RNF31 deficiency significantly enhanced EV-A71 replication (S2E-J Fig), whereas RNF31 overexpression effectively suppressed viral propagation (S2K-M Fig).

### RNF31 enhances the RLR-mediated type I IFN pathway

To elucidate the specific mechanism by which RNF31 inhibits EV-A71 proliferation, we systematically examined its effects on distinct stages of the EV-A71 life cycle. Our results showed neither overexpression nor knock out of RNF31 affected the processes of EV-A71 viral attachment, endocytosis, or translation (S3A-F Fig). Instead, RNF31 significantly suppressed EV-A71 replication efficiency by interfering with the viral replication stage (S3G and S3H Fig). Previous studies have established that RNF31, as an E3 ubiquitin ligase, plays a central role in regulating innate immune and inflammatory signaling pathways [42,43]. On this basis, we next investigated whether RNF31 restricts viral infection by potentiating host antiviral innate immune responses. Using a dual-luciferase reporter assay, we assessed the activities of the IFN-β and IFN-stimulated response element (ISRE) promoters. To establish a well-defined and robust model for innate immune activation, we initially employed Sendai virus (SeV), a potent activator of the RIG-I signaling pathway that is widely used to evaluate IFN-β and ISRE responses [44]. Under SeV stimulation, RNF31 overexpression significantly enhanced the activation of both IFN-β and ISRE promoters (S4A and S4B Fig). Consistent with these findings, RT-qPCR analysis revealed that RNF31 overexpression markedly increased the mRNA levels of IFN-β and the ISG15 (S4C and S4D Fig).

**Fig 4:**
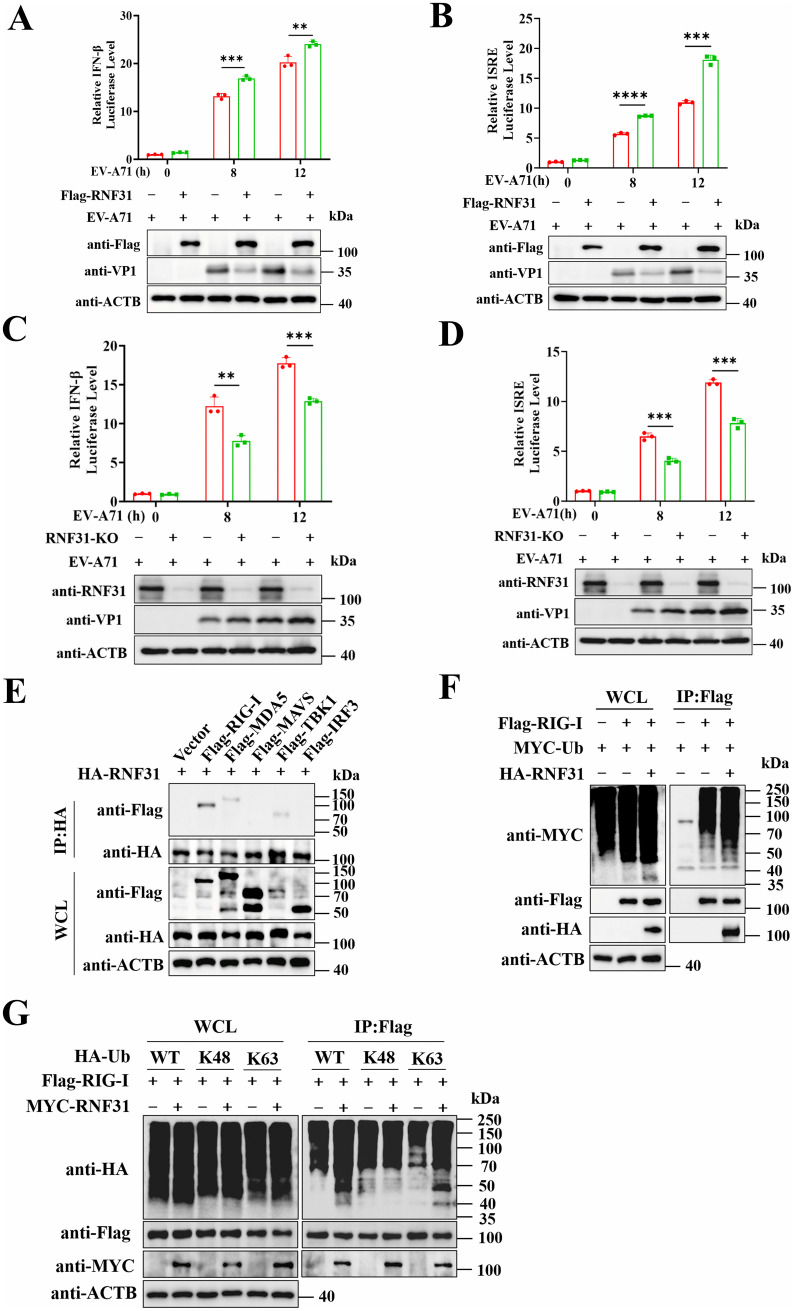
RNF31 enhances the RLR-mediated type I IFN pathway. **(A and B)** RD cells were co-transfected with Flag-RNF31, Renilla luciferase, and IFN-β-Luc or ISRE-Luc reporter plasmids and infected with EV-A71 at 24 h post-transfection. IFN-β (A) and ISRE (B) promoter activities were subsequently determined by luciferase reporter assays. **(C and D)** WT and RNF31-KO RD cells were co-transfected with Renilla luciferase, and IFN-β-Luc or ISRE-Luc reporter plasmids and infected with EV-A71 at 24 h post-transfection. IFN-β (C) and ISRE (D) promoter activities were subsequently determined by luciferase reporter assays. **(E)** Co-IP assays were performed using lysates from HEK293T cells co-transfected with HA-RNF31 and Flag-tagged RIG-I, MDA5, MAVS, TBK1, or IRF3 in the presence of MG132 (10 μM). Immunoprecipitation (IP) was carried out with anti-HA antibodies, followed by IB analysis. **(F)** Co-IP analysis was conducted using lysates from HEK293T cells co-transfected with HA-RNF31, Flag-RIG-I, and MYC-Ub in the presence of MG132 (10 μM). IP was performed with anti-Flag antibodies, followed by IB analysis. **(G)** Co-IP analysis was performed using lysates from HEK293T cells co-transfected with MYC-RNF31, Flag-RIG-I, and HA-Ub (WT, K48, or K63) in the presence of MG132 (10 μM), followed by IP with anti-Flag antibodies and IB analysis. Data are presented as the mean ± SEM from three independent experiments. Statistical significance is indicated as follows: *P < 0.05; **P < 0.01; ***P < 0.001; ****P < 0.0001; ns, not significant.

Vesicular stomatitis virus (VSV) is highly sensitive to IFN-mediated antiviral responses and is commonly used as a model virus to assess how innate immune components regulate viral replication through the IFN signaling pathway [45]. To further clarify the antiviral function of RNF31, HEK293T cells were transfected with RNF31 or an empty vector and subsequently infected with VSV-eGFP. Fluorescence microscopy and flow cytometric analyses demonstrated that RNF31 significantly suppressed VSV-eGFP replication (S4E and S4F Fig). Moreover, during EV-A71 infection, RNF31 overexpression similarly enhanced IFN-β and ISRE promoter activities (Fig 4A and 4B) and increased the mRNA expression of IFN-β and ISG15 (S4G and S4H Fig). In contrast, RNF31 knockout markedly reduced IFN-β and ISRE promoter activities (Fig 4C and 4D) and decreased the mRNA expression of IFN-β and ISG15 (S4I and S4J Fig), further supporting the role of RNF31 in promoting antiviral innate immune responses. Collectively, these results indicate that RNF31 inhibits EV-A71 replication by augmenting the host antiviral innate immune response.

Given the central role of IFN-β production mediated by the RLR signaling pathway in host defense against RNA virus infection, we next investigated the underlying mechanism at which RNF31 functions within this pathway using co-IP assays. The results showed that RNF31 interacted with RIG-I, MDA5, and TBK1, but not with MAVS or IRF3 (Fig 4E). Since ubiquitination of innate immune signaling components is critical for their activation and for subsequent type I interferon production, we further examined the effect of RNF31 on the ubiquitination status of RIG-I, MDA5, and TBK1. Co-IP assays revealed that RNF31 significantly increased the ubiquitination level of RIG-I (Fig 4F), whereas no appreciable changes were observed in the ubiquitination of MDA5 or TBK1 (S4K and S4L Fig). Numerous studies have demonstrated that K63-linked polyubiquitination of RIG-I is essential for its conformational activation and initiation of downstream innate immune signaling [46,47]. Consistent with this, we found that RNF31 markedly enhanced K63-linked ubiquitination of RIG-I (Fig 4G). Collectively, these results indicate that RNF31 promotes RIG-I-mediated type I IFN production and antiviral immune responses by facilitating K63-linked polyubiquitination of RIG-I, thereby suppressing EV-A71 replication.

### RNF31 promotes proteasomal degradation of EV-A71 VP4 protein

**Fig 5:**
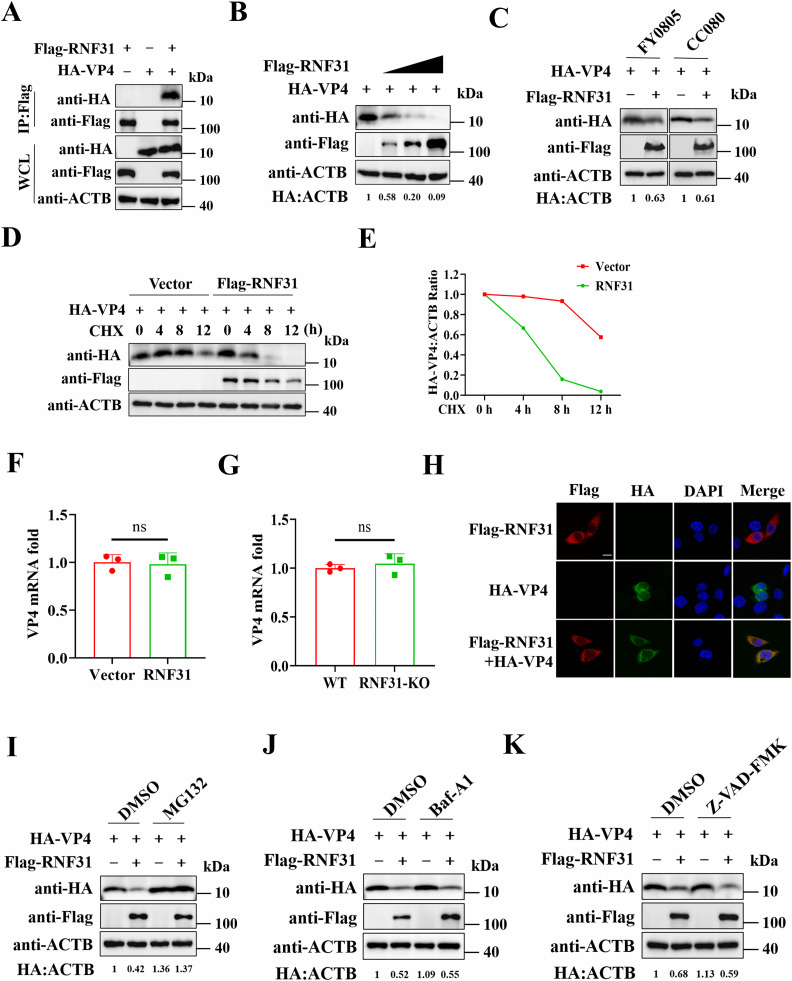
RNF31 promotes proteasomal degradation of EV-A71 VP4 protein. **(A)** HEK293T cells were co-transfected with Flag-RNF31 and HA-VP4 plasmids for 36 h and subsequently treated with MG132 (10 μM). After an additional 12 h, cell lysates were subjected to co-IP using anti-Flag antibodies, followed by IB analysis. **(B)** HEK293T cells were co-transfected with HA-VP4 and increasing amounts of Flag-RNF31, and cell lysates were analyzed by IB at 48 h post-transfection. **(C)** HEK293T cells were co-transfected with HA-VP4 from two additional EV-A71 strains and Flag-RNF31, and cell lysates were analyzed by IB at 48 h post-transfection. **(D and E)** HEK293T cells were co-transfected with HA-VP4 and Flag-RNF31 or an empty control plasmid for 24 h, treated with CHX (50 μg/mL), and harvested at the indicated time points. Cell lysates were analyzed by IB **(D)**, and band intensities were quantified using ImageJ software and normalized to ACTB as an internal control **(E)**. **(F and G)** VP4 mRNA levels were quantified by RT-qPCR in HEK293T cells transfected with HA-VP4 in the presence of Flag-RNF31 overexpression (F) or in RNF31-KO cells **(G)**. **(H)** HeLa cells were co-transfected with Flag-RNF31 and HA-VP4 was examined by confocal laser scanning microscopy. Nuclei were counterstained with DAPI. Scale bars, 10 μm. **(I-K)** HEK293T cells co-transfected with Flag-RNF31 and HA-VP4 for 24 h were treated with DMSO or MG132 **(I)**, Bafilomycin A1 **(J)**, or Z-VAD-FMK (K) for 12 h, followed by IB analysis of cell lysates. Statistical analysis showed no significant differences (ns).

Given that members of the RNF family typically exert their functions through E3 ubiquitin ligase-mediated degradation of target proteins, we investigated whether RNF31 regulates the stability of EV-A71 proteins through the ubiquitin-proteasome pathway. Co-IP assays were first performed to examine the interactions between RNF31 and the EV-A71 structural proteins VP1-VP4. As shown in Fig 5A and S5A-C Fig, RNF31 interacted with VP1, VP2, VP3, and VP4. To determine whether RNF31 promotes degradation of these proteins, expression plasmids encoding VP1-VP4 were co-transfected into HEK293T cells together with increasing amounts of RNF31. IB analysis revealed that VP4 protein levels decreased progressively in an RNF31 dose-dependent manner (Fig 5B), whereas the expression levels of VP1, VP2, and VP3 remained largely unaffected (S5D-S5F Fig). Furthermore, we observed that RNF31 similarly promotes the degradation of the VP4 protein from two additional EV-A71 strains (Fig 5C). These results indicate that RNF31 selectively reduces the protein abundance of VP4. Cycloheximide (CHX) chase assays were subsequently performed to evaluate the effect of RNF31 on VP4 protein stability. RNF31 overexpression markedly accelerated VP4 degradation (Fig 5D and 5E), whereas RNF31 knockout significantly delayed VP4 turnover (S5G and S5H Fig). In parallel, RT-qPCR analysis demonstrated that neither RNF31 overexpression nor knockout altered VP4 mRNA levels (Fig 5F and 5G), indicating that RNF31 regulates VP4 predominantly at the post-translational level. Consistently, immunofluorescence staining revealed clear cytoplasmic colocalization of RNF31 and VP4 (Fig 5H).

In eukaryotic cells, protein degradation primarily occurs through the ubiquitin proteasome system, the autophagy-lysosome pathway, or apoptosis-associated mechanisms [48]. To identify the pathway responsible for RNF31-mediated VP4 degradation, HEK293T cells co-expressing Flag-RNF31 and HA-VP4 were treated with the proteasome inhibitor MG132, the autophagy inhibitor Bafilomycin A1 (Baf A1), or the pan-caspase inhibitor Z-VAD-FMK. MG132 treatment completely abrogated RNF31-induced VP4 degradation, whereas Baf A1 and Z-VAD-FMK had no detectable effect (Fig 5I-K). Moreover, treatment with another proteasome inhibitor, Bortezomib, similarly suppressed RNF31-mediated VP4 degradation (S5I Fig). Collectively, these findings demonstrate that RNF31 promotes degradation of the EV-A71 VP4 protein through the ubiquitin-proteasome pathway.

### RNF31 catalyzes K27/K48-linked polyubiquitination of VP4 at K33 and K51

**Fig 6:**
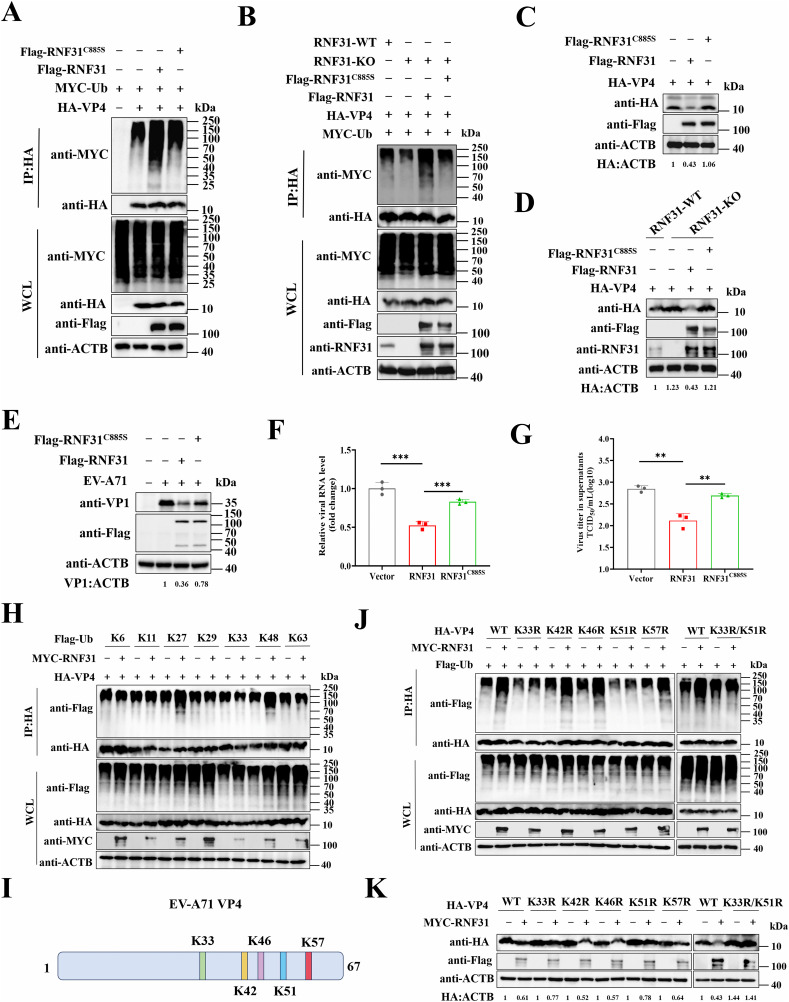
RNF31 catalyzes K27/K48-linked polyubiquitination of VP4 at K33 and K51. **(A)** Co-IP analysis was performed using lysates from HEK293T cells co-transfected with HA-VP4, MYC-Ub, and either Flag-RNF31 or Flag-RNF31^C885S^. IP was conducted with anti-HA antibodies in the presence of MG132 (10 μM), followed by IB analysis. **(B)** HA-VP4 and MYC-Ub were transfected into WT and RNF31-KO HEK293T cells. RNF31-KO cells were simultaneously reconstituted with either Flag-RNF31 or Flag-RNF31^C885S^. After 48 h, co-IP was performed on cell lysates using anti-HA antibodies in the presence of MG132 (10 μM), followed by IB analysis. **(C)** HEK293T cells were transfected with HA-VP4 together with either Flag-RNF31 or Flag-RNF31^C885S^, and cell lysates were analyzed by IB at 48 h post-transfection. **(D)** HA-VP4 was transfected into WT and RNF31-KO HEK293T cells, with RNF31-KO cells simultaneously reconstituted with either Flag-RNF31 or Flag-RNF31^C885S^. Cell lysates were analyzed by IB at 48 h post-transfection. **(E-G)** HEK293T cells were transfected with a control plasmid, Flag-RNF31, or Flag-RNF31^C885S^ for 24 h and subsequently infected with EV-A71. At 48 h post-infection, cell lysates were collected for IB analysis **(E)**, and culture supernatants were harvested for RT-qPCR (F) and TCID₅₀ assays **(G)**. **(H)** Co-IP assays were performed using lysates from HEK293T cells co-transfected with HA-VP4, MYC-RNF31, and either Flag-tagged ubiquitin (WT) or ubiquitin mutants (K6, K11, K27, K29, K33, K48, or K63). IP was conducted with anti-HA antibodies in the presence of MG132 (10 μM), followed by IB analysis. **(I)** Schematic representation of lysine residue distribution within EV-A71 VP4. **(J)** Co-IP analysis was performed using lysates from HEK293T cells co-transfected with MYC-RNF31, Flag-Ub, and either HA-VP4 or its mutants. IP was carried out with anti-HA antibodies in the presence of MG132 (10 μM), followed by IB analysis. **(K)** HEK293T cells were co-transfected with MYC-RNF31 and either HA-VP4 or its mutants, and cell lysates were analyzed by IB at 48 h post-transfection. Statistical significance is indicated as follows: *P < 0.05; **P < 0.01; ***P < 0.001; ****P < 0.0001; ns, not significant.

Given that RNF31 promotes VP4 proteasomal degradation, we investigated whether RNF31 directly catalyzes VP4 ubiquitination. Co-IP assays revealed that RNF31 overexpression significantly increased the ubiquitination level of VP4, an enzymatically inactive RNF31 mutant (RNF31^C885S^) could not increase VP4 ubiquitination (Fig 6A). Moreover, in RNF31-KO HEK293T cells, VP4 ubiquitination was substantially diminished. Reconstitution with RNF31-WT restored VP4 ubiquitination, whereas restoration with RNF31^C885S^ failed to do so (Fig 6B). Consistent with these findings, RNF31^C885S^ failed to promote VP4 degradation, while RNF31 deficiency led to a marked accumulation of VP4 protein. This accumulation was abrogated by complementation with Flag-RNF31, but not with Flag-RNF31^C885S^ (Fig 6C and 6D). These results demonstrate that RNF31 mediates VP4 ubiquitination and subsequent degradation in an E3 ubiquitin ligase-dependent manner. We next evaluated the contribution of RNF31 E3 ligase activity to its antiviral function. Compared with Flag-RNF31, RNF31^C885S^ significantly compromised its ability to suppress EV-A71 replication (Fig 6E-6G). In addition, treatment with HOIPIN-8, a specific inhibitor of RNF31 enzymatic activity, markedly attenuated RNF31-mediated VP4 ubiquitination and concomitantly enhanced EV-A71 replication (S6 Fig). Collectively, these results demonstrate that RNF31 mediates VP4 ubiquitination and proteasomal degradation in an E3 ubiquitin ligase-dependent manner, thereby exerting antiviral activity against EV-A71.

To further characterize the types of polyubiquitin chains conjugated to VP4, plasmids expressing Flag-tagged ubiquitin mutants restricted to individual lysine linkages (K6, K11, K27, K29, K33, K48, or K63) were co-transfected with plasmids encoding MYC-tagged RNF31 and HA-tagged VP4. The results showed that RNF31 markedly enhanced VP4 ubiquitination only in the presence of K27- or K48-linked ubiquitin mutants (Fig 6H), indicating that RNF31 primarily mediates K27- and K48-linked polyubiquitination of VP4. Because ubiquitination typically occurs on lysine residues of target proteins, we next sought to identify the VP4 lysine residues required for RNF31-mediated ubiquitination. Five lysine residues in VP4 were individually mutated to arginine, and their ubiquitination levels were examined (Fig 6I). Mutation of either K33 or K51 significantly reduced RNF31-mediated ubiquitination of VP4, whereas the K33R/K51R double mutation nearly abolished this modification (Fig 6J). Consistent with these findings, the single-point mutants (K33R or K51R) remained partially susceptible to RNF31-induced degradation, whereas the double mutant (K33R/K51R) was completely resistant to RNF31-mediated degradation (Fig 6K). Collectively, these results demonstrate that RNF31, through its E3 ubiquitin ligase activity, catalyzes K27- and K48-linked polyubiquitination of VP4 at lysine residues K33 and K51, thereby promoting proteasomal degradation of VP4 and suppressing EV-A71 replication.

### Cleavage of RNF31 abolished its antiviral activity

**Fig 7:**
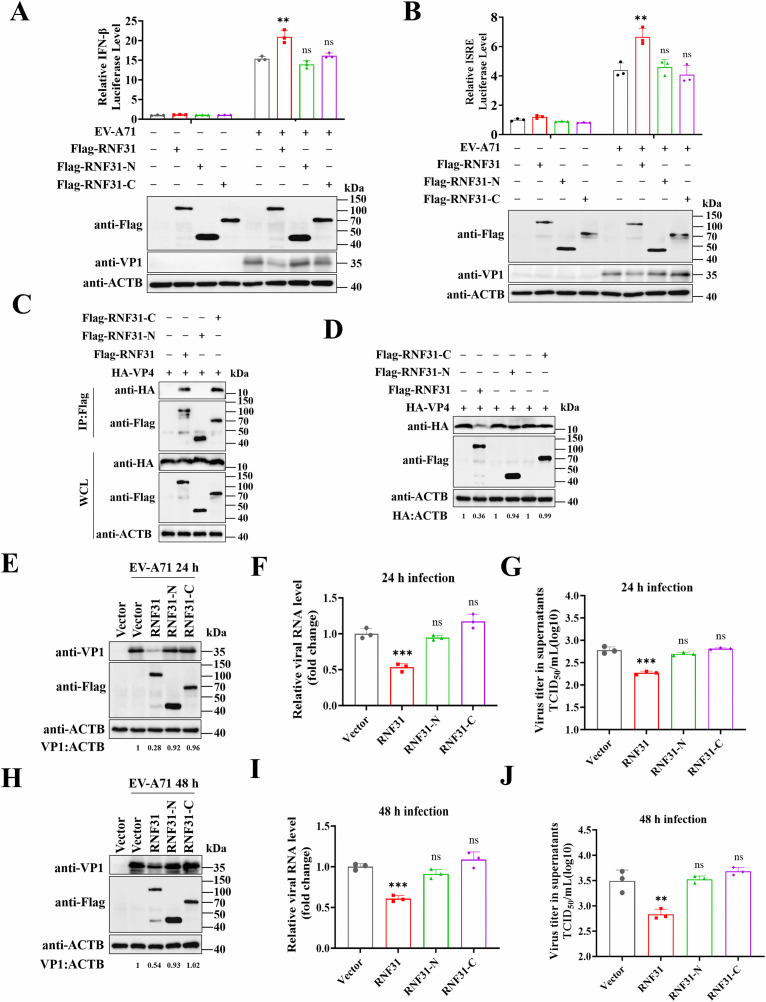
Cleavage of RNF31 abolished its antiviral activity. **(A and B)** RD cells were co-transfected with Flag-RNF31 or its truncated variants, Renilla luciferase, and IFN-β-Luc or ISRE-Luc reporter plasmids, and subsequently infected with EV-A71 at 24 h post-transfection. Following infection, IFN-β (A) and ISRE (B) promoter activities were measured using luciferase reporter assays. **(C)** HEK293T cells were co-transfected with HA-VP4 and either a control plasmid, Flag-RNF31, Flag-RNF31-N, or Flag-RNF31-C. At 36 h post-transfection, cells were treated with MG132 (10 μM) for 12 h, after which cell lysates were subjected to co-IP using anti-Flag antibodies, followed by IB analysis. **(D)** HEK293T cells were transfected with HA-VP4 or a control plasmid together with Flag-RNF31, Flag-RNF31-N, or Flag-RNF31-C, and cell lysates were analyzed by IB at 48 h post-transfection. **(E-J)** RD cells were transfected with a control plasmid, Flag-RNF31, Flag-RNF31-N, or Flag-RNF31-C and subsequently infected with EV-A71 at 24 h post-transfection. Following infection, cell lysates were collected for IB analysis **(E and H)**, and culture supernatants were harvested for RT-qPCR (F and I) analysis and TCID₅₀ determination **(G and J)**. Statistical significance is indicated as follows: *P < 0.05; **P < 0.01; ***P < 0.001; ****P < 0.0001; ns, not significant.

To define the functional consequences of RNF31 cleavage, we analyzed its impact on RIG-I-mediated RLR signaling and EV-A71 VP4 protein degradation. Dual-luciferase reporter assays and RT-qPCR analyses showed that neither the N-terminal (RNF31-N) nor the C-terminal (RNF31-C) cleavage fragment activated IFN-β or ISRE promoter activity (Fig 7A and 7B), nor did they induce the expression of IFN-β or ISG15 (S7A and S7B Fig). These findings indicate that the intact RNF31 protein is required for the activation of antiviral innate immune responses. We then assessed the ability of RNF31 cleavage products to regulate VP4 by co-IP and IB analyses. RNF31-N neither interacted with VP4 nor promoted its degradation. Although RNF31-C retained the ability to bind VP4, it likewise failed to induce VP4 degradation (Fig 7C and 7D). These results suggest that both the N-terminal zinc-finger (ZnF) domain and the C-terminal RBR domain are essential for RNF31-mediated VP4 degradation.

To further determine whether 3Cpro mediated cleavage of RNF31 compromises its antiviral activity, RD cells were transfected with RNF31-WT, RNF31-N, RNF31-C, or an empty vector and subsequently infected with EV-A71. Compared with the empty-vector control, expression of RNF31-N or RNF31-C did not significantly alter viral titers or viral protein levels at 24 h or 48 h post-infection (Fig 7E-J). In contrast, RNF31-WT exhibited a robust antiviral effect. Collectively, these results demonstrate that 3Cpro mediated cleavage of RNF31 disrupts its structural integrity, thereby abolishing its ability to activate the RLR signaling pathway and to mediate VP4 degradation, ultimately negating its antiviral activity against EV-A71.

### RNF31 is a host restriction factor broadly antagonized by enteroviral 3Cpro

**Fig 8:**
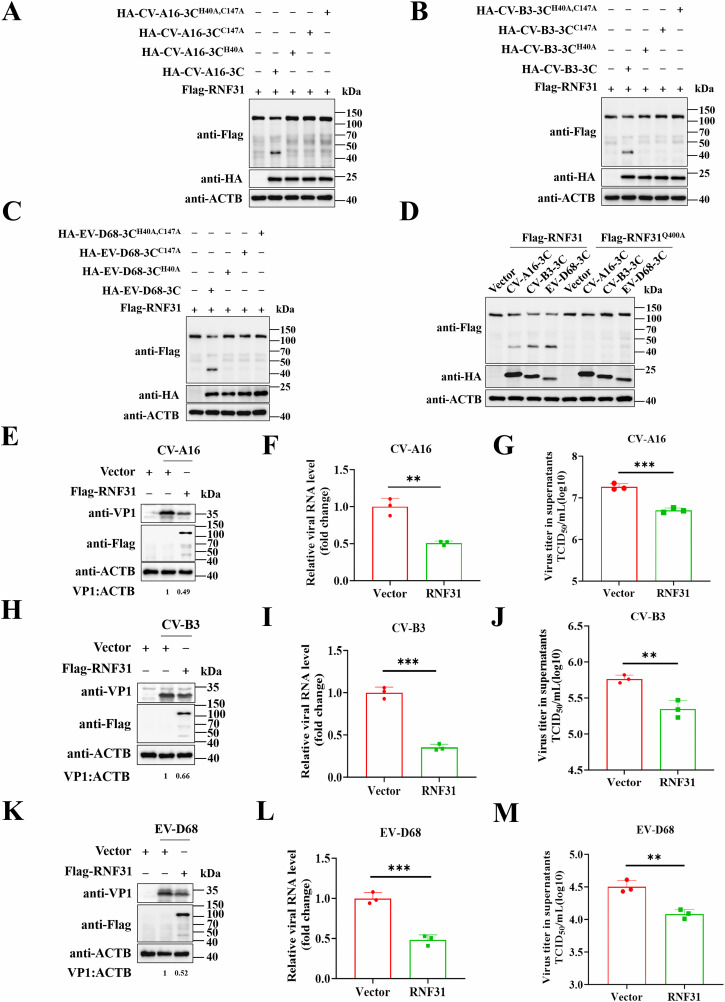
RNF31 is a host restriction factor broadly antagonized by enteroviral 3Cpro. **C**) HEK293T cells were co-transfected with Flag-RNF31 and 3Cpro or its catalytically inactive mutant from CV-A16 **(A)**, CV-B3 **(B)**, or EV-D68 **(C)**. Cell lysates were collected 24 h post-transfection and analyzed by IB. **(D)** HEK293T cells were co-transfected with Flag-RNF31 or the cleavage-resistant mutant Flag-RNF31^Q400A^ together with plasmids expressing the 3Cpro of CV-A16, CV-B3, or EV-D68. Cell lysates were analyzed by IB at 24 h post-transfection. **(E-G)** RD cells were transfected with a control plasmid or Flag-RNF31 and infected with CV-A16 at 24 h post-transfection. At 48 h post-infection, cell lysates were analyzed by IB **(E)**, and culture supernatants were collected for RT-qPCR (F) and TCID₅₀ assays **(G)**. **(H-J)** RD cells were transfected with a control plasmid or Flag-RNF31 and infected with CV-B3 at 24 h post-transfection. At 48 h post-infection, cell lysates were analyzed by IB **(H)**, and supernatants were used for RT-qPCR (I) and TCID₅₀ assays **(J)**. **(K-M)** RD cells were transfected with a control plasmid or Flag-RNF31 and infected with EV-D68 at 24 h post-transfection. At 48 h post-infection, cell lysates were analyzed by IB **(K)**, and supernatants were collected for RT-qPCR (L) and TCID₅₀ assays **(M)**. Statistical significance is indicated as follows: *P < 0.05; **P < 0.01; ***P < 0.001; ****P < 0.0001; ns, not significant.

Finally, We investigated whether RNF31 could be cleaved by 3Cpro from other enteroviruses. Previous studies have shown that enteroviral 3Cpro proteins are highly conserved in both structure and function, particularly within their catalytic core regions. Based on this conservation, RNF31 was co-transfected with either WT or protease-deficient 3Cpro from CV-A16, CV-B3, and EV-D68. The results demonstrated that all WT 3Cpro proteins efficiently cleaved RNF31, whereas the corresponding protease-inactive mutants completely lost this activity (Fig 8A-C). Further analyses revealed that 3Cpro from CV-A16, CV-B3, and EV-D68 cleaved RNF31 at the same site (Fig 8D), indicating that Q400 represents a conserved cleavage site targeted by multiple enteroviral 3Cpro proteins, consistent with the cleavage pattern observed for EV-A71 3Cpro.

Having established that RNF31 is cleaved by 3Cpro from multiple enteroviruses, we next assessed whether RNF31 exerts antiviral activity against CV-A16, CV-B3, and EV-D68. Ectopic expression of RNF31 significantly reduced both mRNA and protein levels of the viral capsid protein VP1 from all three viruses and markedly suppressed viral replication, as measured by the TCID₅₀ assay (Fig 8E-M). Furthermore, given that RNF31 restricts EV-A71 replication by promoting degradation of the viral VP4 protein, we examined whether RNF31 regulates other enteroviruses through a similar mechanism. Co-expression of RNF31 with VP4 from CV-A16, CV-B3, or EV-D68 resulted in a pronounced reduction in VP4 protein levels from all three viruses (S8 Fig). Collectively, these findings demonstrate that enteroviral 3Cpro cleave the host restriction factor RNF31 to antagonize its antiviral function and suggest that RNF31-associated immune regulatory pathways represent a conserved target exploited by multiple enteroviruses.

## Discussion

**Fig 9:**
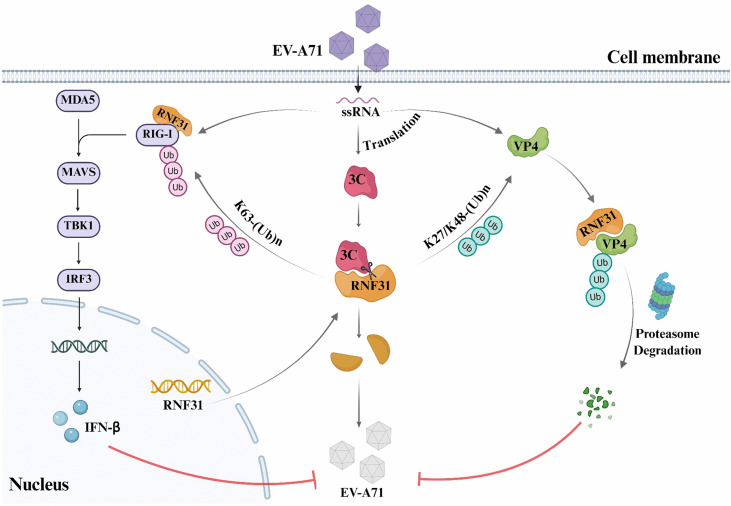
A Proposed model depicting how EV-A71 3Cpro evades RNF31-mediated antiviral responses. Created in BioRender. Gao, Y. (2026). https://BioRender.com/sufoyg1.

Enteroviruses constitute a major group of human pathogens responsible for a wide range of diseases, including hand, foot, and mouth disease, myocarditis, and neurological complications. Among them, EV-A71, CV-A16,CV-B3, and EV-D68 are frequently associated with recurrent outbreaks and, in some cases, severe public health emergencies. The ubiquitin system serves as a critical regulatory hub in innate immunity, not only fine-tuning RLR signaling but also directly restricting viral replication through the targeted degradation of viral proteins [49]. However, the identity of key host restriction factors and the mechanisms by which viruses antagonize their antiviral functions remain incompletely understood. In this study, we demonstrate that RNF31 restricts enterovirus infection through a dual mechanism. Firstly, RNF31 enhances RLR-mediated innate immune signaling by promoting K63-linked ubiquitination of RIG-I, thereby inducing the expression of type I IFN and IFN-stimulated genes. Secondly, RNF31 acts as an E3 ubiquitin ligase to directly target the EV-A71 structural protein VP4, promoting its K27/K48-linked polyubiquitination and subsequent proteasomal degradation, which suppresses viral replication at the protein level. This coordinated mode of action-integrating immune regulation with direct antiviral activity highlights the multilayered role of RNF31 in host defense against enteroviruses. Nevertheless, enteroviruses have evolved an immune evasion strategy in which their 3C protease cleaves RNF31, thereby disabling its antiviral functions and facilitating viral replication (Fig 9).

RNF31 was initially identified as a core component of the LUBAC, where it promotes ubiquitination of key signaling molecules such as RIPK1 and NEMO by catalyzing the formation of M1 linear ubiquitin chains in response to inflammatory stimuli [34,50]. This activity leads to activation of the NF-κB signaling pathway. Increasing evidence indicates that the function of RNF31 is not restricted to linear ubiquitination; rather, it can also catalyze non-M1 ubiquitin linkages to regulate the stability and function of diverse substrate proteins. For example, RNF31 has been reported to promote K63-linked ubiquitination of NLRP3, thereby enhancing inflammasome activation. The present study further expands the functional repertoire of RNF31 by elucidating its role in antiviral innate immunity, demonstrating that RNF31 enhances RIG-I-mediated signaling through the promotion of K63-linked ubiquitination of RIG-I. Conversely, EV-A71 antagonizes this pathway by cleaving RNF31, thereby suppressing RIG-I-dependent RLR signaling. This observation is consistent with findings reported by Chen et al, showing that EV-A71 infection reduces RIG-I ubiquitination and impairs type I IFN signaling [51].

VP4 of EV-A71 is a small structural protein located within the viral capsid that plays a critical role in viral entry, uncoating, and genome release, and contributes to the assembly efficiency and infectivity of viral particles. Although VP4 is essential for the viral life cycle, its potential as a target of host antiviral defenses has rarely been explored. This study provides the first experimental evidence that a host E3 ubiquitin ligase can specifically target and degrade VP4 via the ubiquitin-proteasome pathway. Mechanistically, RNF31 mediates K27- and K48-linked polyubiquitination of VP4 at lysine residues K33 and K51, thereby promoting its proteasomal degradation without affecting the stability of other viral structural proteins. These findings suggest that the host does not indiscriminately eliminate viral proteins but instead selectively targets critical structural components of the viral life cycle to achieve efficient suppression of viral replication. Notably, EV-A71 cleaves RNF31 at the Q400 site via its 3Cpro, generating N-terminal and C-terminal fragments. Although the C-terminal fragment retains the ability to bind VP4, it fails to promote VP4 degradation, indicating that the intact structure of RNF31 and the coordinated action of its functional domains are required for efficient VP4 degradation.

Previous studies have demonstrated that EV-A71 cleaves multiple key immune signaling molecules through its 3C protease, including TRIF, TAK1, TAB1, IRF7, OAS3, and NLRP3, thereby disrupting innate immune signaling pathways at multiple levels and enabling immune evasion [15,18–20,52]. These studies have largely focused on viral antagonism of signal transduction components or effector molecules, elucidating how EV-A71 promotes its own replication by impairing immune signaling cascades. However, whether EV-A71 employs an immune evasion strategy that directly targets core regulatory components of the host ubiquitin system-particularly E3 ubiquitin ligases has remained largely unexplored. In the present study, we demonstrate that EV-A71 specifically targets and cleaves the E3 ubiquitin ligase RNF31 via its 3Cpro, thereby effectively abrogating its antiviral function. Notably, this mechanism is conserved among enteroviruses, as similar cleavage of RNF31 was observed for CV-A16, CV-B3, and EV-D68. These findings uncover an additional layer of enteroviral immune evasion, indicating that EV-A71 not only interferes with downstream immune signaling outputs but also broadly weakens host antiviral defenses by disrupting an upstream regulatory hub of ubiquitination.

In this study, the inhibitory effect of RNF31 on the replication of multiple enteroviruses was validated at the cellular level using both overexpression and knockout approaches. Because RNF31-knockout mice exhibit an embryonic lethal phenotype, direct assessment of its antiviral function in vivo is currently not feasible, representing a limitation of the present study [53]. It warrants further investigation that the specific recognition and cleavage of RNF31 by the EV-A71 3Cpro suggest a defined substrate preference and structural selectivity. Based on this property, future studies may explore the use of RNF31-derived substrate sequences recognized by 3Cpro to design competitive inhibitory peptides or peptidomimetics that bind to the protease active site while resisting proteolytic cleavage. Such inhibitors could effectively block 3Cpro activity and thereby suppress viral replication. Importantly, this strategy is directly inspired by the authentic substrate interface exploited during host-virus interactions and may offer enhanced binding specificity and antiviral efficacy.

Additional several limitations of the present study highlight important directions for future research. First, although RNF31 cleavage and its antiviral effects were identified in several enteroviruses, we did not assess whether similar effects occur in other viruses, such as rhinoviruses and coronaviruses. Second, while the interaction between RNF31 and EV-A71 was validated in RD cells and HEK293T cells, it remains unclear whether this interaction occurs in the natural target cells of EV-A71 infection. Extending these findings to physiologically relevant human cell types, including neuronal cells, skin cells, and oral epithelial cells, would further enhance the clinical and translational relevance of our results.

In summary, this study reveals a previously unrecognized antiviral mechanism in which RNF31 restricts EV-A71 replication by enhancing RLR-mediated signaling transduction and promoting degradation of the viral VP4 protein, while also elucidating how EV-A71 counteracts this host defense through 3Cpro mediated cleavage of RNF31. These findings not only expand our understanding of RNF31 function in antiviral innate immunity but also uncover a novel strategy by which enteroviruses exploit viral proteases to target host E3 ubiquitin ligases and facilitate their own replication. Collectively, this work provides a conceptual framework for the future development of host-directed antiviral therapeutics and more effective vaccine strategies against enteroviruses.

## Materials and methods

### Cells and viruses

HEK293T (American Type Culture Collection [ATCC], catalog no. CRL-11268), Human Rhabdomyosarcoma RD (ATCC, catalog no. CCL-136) and HeLa (ATCC, catalog no. CRM-CCL-2) were cultured as monolayers in Dulbecco’s modified Eagle’s medium (DMEM) (Gibco, 11965–092), which was supplemented with 10% heat-inactivated (56°C, 30 min) fetal bovine serum (FBS, ST30–3302, PAN Seratech), and 50 IU/mL penicillin, 100 mg/mL streptomycin (03–031-1B, Biological Industries), 12.5 ng/mL amphotericin B solution (C0224, Beyotime). Cells were maintained in a humidified environment at 37°C with 5% CO_2_. All cell lines have been confirmed to be mycoplasma-free. EV-A71 strain CC063 was isolated and characterized from HFMD patients by Wang et al in 2010. CV-A16 strain CC024 was isolated and characterized by Li et al. Both the EV-D68 strain US/KY/14–18953 and CV-B3 strain US/CT/Nancy were purchased from ATCC. All viruses were titered using the median tissue culture infectious dose (TCID_50_) assay.

### Antibodies and reagents

The following antibodies were used in this study: Anti-Flag mouse monoclonal antibody (mAb) (Sigma, catalog no. F1804), anti-HA mouse mAb (Sigma, catalog no. H3663), anti-ACTB/β-actin mouse mAb (GenScript, catalog no. A00702), anti-MYC rabbit polyclonal antibody (pAb) (Proteintech, catalog no. 16286–1-AP), anti-Flag rabbit pAb (Proteintech, catalog no. 20543–1-AP), anti-HA rabbit pAb (Invitrogen, catalog no. 71–5500), anti-RNF31/HOIP rabbit mAb (Abcam, catalog no. ab315162), anti-pro-CASP3/caspase 3 rabbit mAb (ABclonal, catalog no. A19654), anti-CASP3/caspase 3 rabbit mAb (ABclonal, catalog no. A19664), anti-GFP mouse mAb (Abcam, catalog no. ab1218), horse radish peroxidase (HRP)-conjugated secondary antibody (Jackson Immunoresearch, catalog no. 115-035-062 for anti-mouse and 111-035-045 for anti-rabbit), Alexa Fluor 488-labeled goat anti-mouse IgG (Invitrogen, catalog no. A11001), Alexa Fluor 647-labeled goat anti-rabbit IgG (Invitrogen, catalog no. A32733), anti-EV-A71 VP1 rabbit mAb (Abcam, catalog no. ab308206), anti-EV-D68 VP1 rabbit mAb (Abcam, catalog no. ab308217), anti-CV-A16 VP1 rabbit pAb and anti-CV-B3 VP1 rabbit pAb were prepared and stored by our laboratory.

The chemical reagents used in this study are as follows: MG132 (Selleck, catalog no. S2619), Bortezomib (Selleck, catalog no. S1013), Bafilomycin A1 (Selleck, catalog no. S1413), TNF-α (Selleck, catalog no. E7641), Z-VAD-FMK (Med Chem Express, catalog no. HY-16658B), CHX (Sigma, catalog no. 66-81-9), Rupintrivir (Med Chem Express, catalog no. HY-106161), HOIPIN-8 (Med Chem Express, catalog no. HY-122882).

### Plasmids and transfection

Full-length RNF31 was amplified by PCR using cDNA derived from HEK293T cells as a template and cloned into the VR1012 vector between the SalI and BamHI restriction sites, with a Flag tag fused to the N terminus. Flag-tagged RNF31 point mutants (Q340A, Q357A, Q400A, Q409A, and C885S), as well as the dual-tagged construct Flag-RNF31-MYC, were generated from the Flag-RNF31 plasmid by site-directed mutagenesis using PCR. Truncated RNF31 variants (RNF31-N and RNF31-C) were constructed from the Flag-RNF31 template by homologous recombination and inserted into the SalI and BamHI sites of the VR1012 vector. Mutant forms of the 3Cpro (H40A, C147A, and H40A/C147A) from these four enteroviruses, as well as EV-A71 VP4 mutants (K33R, K42R, K46R, K51R, K57R, and K33R/K51R), were generated by PCR-based site-directed mutagenesis. Expression plasmids encoding viral proteins from EV-A71, EV-D68, CV-A16, CV-B3 and all other plasmids were obtained from our laboratory. All constructs were verified by DNA sequencing. Plasmids were transfected into HEK293T, RD, and HeLa cells using Lipofectamine 2000 (Invitrogen; catalog no. 11668–019) according to the manufacturer’s instructions. The sequences of all oligonucleotide primers used in this study are listed in S1 Table.

### Virus infection

HEK293T and RD cells were transfected with overexpression plasmids using Lipofectamine 2000 when they reached approximately 80% confluence. At 24 h post-transfection, cells were washed once with phosphate-buffered saline (PBS) and infected by adding virus-containing DMEM at a multiplicity of infection (MOI) of 0.1. Plates were incubated at 37°C with gentle agitation every 20 min to ensure uniform viral adsorption. After infection, the inoculum was removed and replaced with fresh DMEM supplemented with 2% FBS. Cells were then cultured for the indicated durations according to experimental requirements. At the designated time points, cell lysates and culture supernatants were collected. Viral titers in the supernatants were determined using TCID₅₀ assay, and intracellular viral protein expression was analyzed by IB.

### CRISPR-Cas9-mediated RNF31 gene knockout

RNF31-knockout HEK293T and RD cell lines were generated using the CRISPR/Cas9 system. A single-guide RNA (sgRNA) targeting RNF31 (5′-CACCGGGCGCTCGCCAGCTCCTCG-3′) was designed using the online tool CCTop (https://cctop.cos.uni-heidelberg.de). After annealing, the sgRNA oligonucleotides were cloned into the lentiCRISPR v2 vector at the BsmBI restriction sites. The resulting lentiCRISPR v2-RNF31-sgRNA plasmid or the empty vector control, together with the packaging plasmids RRE, REV, and VSV-G, were co-transfected into HEK293T cells cultured in 10-cm dishes. Lentivirus-containing supernatants were collected 48 h post-transfection, filtered, and titrated using a lentivirus detection kit. The viral supernatants were then used to infect HEK293T and RD cells seeded in 12-well plates. 48 h after infection, puromycin selection was performed using 2 μg/mL for HEK293T cells and 0.5 μg/mL for RD cells (Sigma, catalog no. P8833). Surviving cells were serially diluted and plated into 96-well plates to isolate single-cell clones. After clonal expansion, RNF31-knockout cell lines were confirmed by genomic DNA sequencing and IB analysis.

### Cell viability assay

Cell viability was evaluated using a Cell Counting Kit-8 (CCK-8; TransGen Biotech, catalog no. FC101–02). Wild-type (WT) and RNF31-knockout cells were seeded into 96-well plates, and cell viability was assessed at 24 h and 48 h post-seeding. Following the addition of CCK-8 reagent (10 μL per well), plates were incubated at 37°C for 1 h. Absorbance was then measured at 450 nm using a microplate reader.

### RNA extraction and RT-qPCR

Intracellular and viral RNA were extracted from the indicated cell types using TRIzol reagent (Invitrogen; catalog no. 15596018CN). The isolated RNA was reverse transcribed into cDNA using a High-Capacity cDNA Reverse Transcription Kit (Applied Biosystems, catalog no. 4368814) according to the manufacturer’s instructions. Real-time quantitative PCR (RT-qPCR) was performed on an Mx3005P system (Agilent Technologies/Stratagene) using RealMaster Mix (SYBR Green Kit; Roche, catalog no. 491314001). The amplification protocol consisted of an initial activation of HotMaster Taq DNA polymerase at 95°C for 2 min, followed by 40 cycles of denaturation at 95°C for 15 s, annealing at 57°C for 15 s, and extension at 68°C for 20 s. The sequences of all oligonucleotide primers used for RT-qPCR are listed in S2 Table.

### Dual-luciferase reporter assays

HEK293T or RD cells seeded in 12-well plates were transfected with the indicated expression plasmids or empty vectors using Lipofectamine 2000. Cells were co-transfected with a firefly luciferase reporter plasmid and 1 ng of a Renilla luciferase plasmid as an internal control. At 24 h post-transfection, cells were infected with Sendai virus (SeV) or EV-A71 for the indicated durations. Cells were harvested at the specified time points, and luciferase activities were measured using the Dual-Luciferase Reporter Assay System (Promega; catalog no. E1910) according to the manufacturer’s instructions. Luminescence was recorded using a GloMax 20/20 luminometer (Promega).

### Immunofluorescence assay (IFA)

HeLa cells seeded in glass-bottom culture dishes were transfected with the indicated expression plasmids or control vectors. At 48 h post-transfection, the culture medium was removed, and cells were washed twice with prewarmed PBS for 5 min each. Cells were then fixed with 4% paraformaldehyde at 37°C for 10 min. After three washes with PBS, cells were permeabilized with 0.25% Triton X-100 at 37°C for 10 min. Following an additional three PBS washes, cells were blocked with 10% FBS at room temperature for 1 h. Cells were subsequently incubated overnight at 4°C with the appropriate primary antibodies. After three PBS washes, cells were incubated with Alexa Fluor-conjugated secondary antibodies at room temperature in the dark for 1 h. Nuclei were then counterstained with 4′,6-diamidino-2-phenylindole (DAPI; Sigma, catalog no. D9542) for 2 min, followed by mounting in 90% glycerol. Fluorescence images were acquired using a laser-scanning confocal microscope (Olympus; FV3000).

### Western blotting assay (Immunoblotting)

Transfected or infected HEK293T or RD cells were harvested and lysed on ice for 30 min in RIPA buffer (50 mM Tris-HCl [pH 7.8], 150 mM NaCl, 1.0% NP-40, 5% glycerol, and 4 mM EDTA), with gentle mixing every 10 min. Cell lysates were clarified by centrifugation to remove insoluble debris, and the resulting supernatants were mixed with 4 × SDS loading buffer (0.08 M Tris-HCl [pH 6.8], 2.0% SDS, 10% glycerol, 0.1 M dithiothreitol, and 0.2% bromophenol blue) and boiled at 100°C for 5 min. Proteins were separated by denaturing electrophoresis on 12.5% SDS-PAGE gels and transferred onto polyvinylidene fluoride (PVDF) membranes. Membranes were blocked with 5% nonfat milk at room temperature for 1 h and incubated overnight at 4°C with primary antibodies. After washing, membranes were incubated with HRP-conjugated secondary antibodies diluted 1:10000. Protein bands were visualized using an ultrasensitive ECL chemiluminescence detection kit (Proteintech; catalog no. B500024), and images were captured using a digital imaging system. Protein band intensities were quantified using ImageJ software.

### Co-immunoprecipitation (Co-IP)

HEK293T cells treated with MG132 (10 μM) for 10 h were harvested, resuspended in 1 mL of lysis buffer (50 mM Tris-HCl [pH 7.5], 150 mM NaCl, 1% NP-40), and lysed by sonication for 1 min using an ultrasonic cell disruptor. Cell lysates were clarified by centrifugation to remove insoluble debris, and the resulting supernatants were incubated with antibody-conjugated protein G agarose beads overnight at 4°C. The following day, beads were washed six to eight times with wash buffer (20 mM Tris-HCl [pH 7.5], 100 mM NaCl, 0.1 mM EDTA, and 0.05% Tween-20) at 4°C, with centrifugation at 800 × *g* for 1 min between washes. Bound proteins were eluted by incubation with elution buffer (0.1 M glycine-HCl [pH 2.5]) and boiling at 100°C for 10 min. Eluted proteins were subsequently analyzed by IB.

### Protein Purification

EV-A71 3Cpro was cloned into the prokaryotic expression vector pET-28a with an N-terminal 6 × His tag. The recombinant plasmid was transformed into *Escherichia coli* BL21 (DE3) competent cells (TransGen Biotech, catalog no. CD601–02), and protein expression was induced in 3 L of bacterial culture. When the optical density at 600 nm (OD₆₀₀) reached approximately 0.6, protein expression was induced with 0.5 mM isopropyl β-D-1-thiogalactopyranoside (IPTG) and carried out overnight for 12 h at 16°C with shaking at 100 rpm. Bacterial cells were harvested by centrifugation at 6000 × *g* for 6 min and resuspended in 50 mL of HEPES buffer (20 mM HEPES [pH 7.5], 200 mM NaCl), followed by sonication at 4°C. Cell lysates were clarified by centrifugation at 12000 rpm for 30 min, and the supernatants were filtered through a 0.45 μm membrane. The His-tagged 3Cpro protein was subsequently purified by Ni-NTA affinity chromatography and eluted using imidazole at increasing concentrations. Fractions containing the target protein were pooled, supplemented with 10% glycerol, and stored at -80°C until use. RNF31 protein was purified using a eukaryotic expression system. HEK293T cells cultured in 10-cm dishes were transfected with Flag-tagged RNF31 and harvested 48 h post-transfection. RNF31 was enriched using Flag antibody-conjugated protein G agarose beads following the co-IP protocol and eluted by competition with Flag peptide at 4°C for 4 h.

### RNF31 in vitro cleavage assay

Purified EV-A71 3Cpro and RNF31 protein were mixed in cleavage buffer (50 mM Tris-HCl [pH 7.3], 1 mM EDTA) and incubated at 30°C for 2 h. The incubated samples were analyzed by SDS-PAGE, stained with Coomassie Brilliant Blue, and photographed.

### Flow cytometry

HEK293T cells were transfected with either an RNF31 expression plasmid or an empty control vector for 24 h. Cells were then infected with vesicular stomatitis virus expressing green fluorescent protein (VSV-eGFP) for 12 h. After infection, cells were gently harvested by 0.25% trypsinization, centrifuged at 1000 rpm for 5 min, and resuspended in PBS. The resulting single-cell suspensions were filtered and immediately analyzed by flow cytometry to evaluate the antiviral effect of RNF31 overexpression on VSV-GFP infection. The proportion of GFP-positive cells was quantified at 12 h post-infection.

### Mass spectrometry (MS)

HEK293T cells cultured in 10-cm dishes were transfected with HA-tagged EV-A71 3Cpro, the catalytically inactive mutant HA-EV-A71 3C^H40A,C147A^, or the empty vector VR1012 for 36 h, followed by treatment with MG132 for 10 h prior to cell harvest. Proteins were enriched by co-IP, and the resulting eluates were subjected to mass spectrometry analysis. Mass spectrometry was performed at the National Center for Protein Science (Beijing, China).

### Statistical analysis

All results are based on three independent experiments and are expressed as ± standard deviation (SDs). Data synthesis and analysis were performed using GraphPad Prism software. Statistical analysis was conducted using an unpaired, two-tailed Student’s *t*-test, one-way ANOVA followed by Dunett’s multiple comparisons test, or two-way ANOVA followed by Sidak’s multiple comparisons test. The differences are statistically significant: *P < 0.05; **P < 0.01; ***P < 0.001; ****P < 0.0001); ns, no significance.

## Supporting information

S1 Fig Identified RNF31 as a substrate protein of EV-A71 3Cpro. (**A**) Schematic overview of the LC-MS/MS-based experimental workflow. Samples were prepared for LC-MS/MS analysis, and proteins interacting with 3C or the catalytically inactive mutant 3C^H40A,C147A^ were enriched by co-IP. Created in BioRender. Gao, Y. (2026). https://BioRender.com/mv61d2l. (**B**) HEK293T cells were transfected with a control plasmid, HA-3C, or HA-3C^H40A,C147A^ for 36 h and subsequently treated with MG132 (10 μM) for 12 h. Cell lysates were incubated with anti-HA antibody-conjugated protein G agarose beads, followed by IB using anti-HA antibodies. The enriched protein complexes were then subjected to MS analysis. (**C**) LC-MS/MS analysis identified proteins interacting with 3Cpro and preferentially enriched in 3Cpro^H40A,C147A^. Candidates were selected based on a binding enrichment ratio >5 for 3Cpro versus empty vector (left) and >2 for 3Cpro^H40A,C147A^ versus 3Cpro (right), yielding five candidate proteins. (**D**) HeLa cells were co-transfected with Flag-RNF31 and either HA-3C or HA-3C^H40A,C147A^. Co-localization of RNF31 with 3C or 3C^H40A,C147A^ was examined by confocal laser scanning microscopy. Nuclei were counterstained with DAPI. Scale bar, 10 μm.
(TIF)

S2 Fig RNF31 inhibits EV-A71 replication in HEK293T cells. (**A**) Schematic illustration of the CRISPR-Cas9 strategy used to generate RNF31-KO cells. (**B and C**) Validation of RNF31 knockout in RD cells by genomic DNA sequencing (B) and IB analysis (C). (**D**) Cell viability of WT and RNF31-KO RD cell lines. (**E and F**) Validation of RNF31 knockout in HEK293T cells by genomic DNA sequencing (E) and IB analysis (F). (**G**) Cell viability of WT and RNF31-KO HEK293T cell lines. (**H-J**) WT and RNF31-KO HEK293T cells were infected with EV-A71 for 24 h or 48 h. Following infection, cell lysates were analyzed by IB (H), and culture supernatants were subjected to RT-qPCR (I) and TCID₅₀ assays (J). (**K-M**) HEK293T cells transfected with Flag-RNF31 or control plasmids were infected with EV-A71 for 24 h or 48 h. After infection, cell lysates were analyzed by IB (K), and supernatants were collected for RT-qPCR (L) and TCID₅₀ assays (M). Data are presented as the mean ± SEM from three independent experiments. Statistical significance is indicated as follows: *P < 0.05; **P < 0.01; ***P < 0.001; ****P < 0.0001; ns, not significant.
(TIF)

S3 Fig RNF31 modulates the replication process of EV-A71. (**A and B**) WT (A) or RNF31-KO (B) RD cells were transfected with a control plasmid or Flag-RNF31. At 24 h post-transfection, cells were infected with EV-A71 at 4°C. After 1 h of adsorption, cells were washed three times with PBS, harvested, and subjected to RT-qPCR to quantify viral RNA copy numbers, thereby assessing the effect of RNF31 on EV-A71 attachment.Y-axis indicates viral RNA levels expressed as log_10_ copies/mL. (**C and D**) WT (C) or RNF31-KO (D) RD cells were transfected with a control plasmid or Flag-RNF31 for 24 h and infected with EV-A71 at 4°C. After 1 h of adsorption, cells were washed three times with PBS, replaced with maintenance medium, and incubated at 37°C in a 5% CO₂ incubator to allow viral entry. Following 1 h of incubation, cells were washed twice with pre-chilled PBS, once with pre-chilled alkaline high-salt solution, and twice again with pre-chilled PBS. Cells were then collected for RT-qPCR analysis to quantify viral RNA copy numbers and evaluate the effect of RNF31 on EV-A71 entry. (**E and F**) WT (E) or RNF31-KO (F) RD cells were transfected with a control plasmid or Flag-RNF31 and infected with EV-A71 as described above. Cells were harvested for IB at 4 hpi to determine viral protein levels, thereby assessing the effect of RNF31 on EV-A71 translation. Relative viral protein levels were quantified by densitometric analysis of IB results and normalized to ACTB. (**G and H**) WT (G) or RNF31-KO (H) RD cells were transfected with a control plasmid or Flag-RNF31 and infected with EV-A71 as described above. Cells were harvested at 8 hpi for RT-qPCR to quantify viral RNA copy numbers, thereby evaluating the effect of RNF31 on EV-A71 replication. Statistical significance is indicated as follows: *P < 0.05; **P < 0.01; ***P < 0.001; ****P < 0.0001; ns, not significant.
(TIF)

S4 Fig RNF31 enhances the RLR signaling pathway by promoting K63-linked polyubiquitination of RIG-I. **(A and B)** HEK293T cells were co-transfected with Flag-RNF31, Renilla luciferase, and IFN-β-Luc or ISRE-Luc reporter plasmids and subsequently infected with SeV at 24 h post-transfection. After 12 h of infection, IFN-β (A) and ISRE (B) promoter activities were measured using a dual-luciferase reporter assay. **(C and D)** HEK293T cells were transfected with Flag-RNF31 and subsequently infected with SeV at 24 h post-transfection. After 12 h of infection, the mRNA expression levels of IFN-β (C) and ISG15 (D) were quantified by RT-qPCR. (**E and F**) HEK293T cells were transfected with Flag-RNF31 or an empty control plasmid for 24 h and then infected with VSV-GFP for 12 h. Following infection, cells were fixed and examined by confocal microscopy (E) or quantified by flow cytometry (F). Scale bar, 200 μm. (**G and H**) RD cells were transfected with Flag-RNF31 and infected with EV-A71 at 24 h post-transfection. Following infection, the mRNA expression levels of IFN-β (G) and ISG15 (H) were measured by RT-qPCR. (**I and J**) WT or RNF31-KO RD cells transfected with Flag-RNF31 and infected with EV-A71 at 24 h post-transfection. Following infection, the mRNA expression levels of IFN-β (I) and ISG15 (J) were measured by RT-qPCR. (**K**) Co-IP assays were performed using lysates from HEK293T cells co-transfected with HA-RNF31, Flag-MDA5, and MYC-Ub in the presence of MG132 (10 μM). IP was carried out with anti-Flag antibodies, followed by IB analysis. (**L**) Co-IP analysis was conducted using lysates from HEK293T cells co-transfected with HA-RNF31, Flag-TBK1, and MYC-Ub in the presence of MG132 (10 μM), followed by IP with anti-FLAG antibodies and IB analysis. Statistical significance is indicated as follows: *P < 0.05; **P < 0.01; ***P < 0.001; ****P < 0.0001; ns, not significant.
(TIF)

S5 Fig RNF31 promotes proteasomal degradation of EV-A71 VP4 protein. (**A-C**) HEK293T cells were co-transfected with Flag-RNF31 and either HA-VP1 (A), HA-VP2 (B), or HA-VP3 (C) for 36 h and subsequently treated with MG132 (10 μM). After an additional 12 h, cell lysates were subjected to co-IP using anti-Flag antibodies, followed by IB analysis. **(D-F**) HEK293T cells were co-transfected with increasing amounts of Flag-RNF31 together with HA-VP1 (D), HA-VP2 (E), or HA-VP3 (F). Cell lysates were harvested at 48 h post-transfection and analyzed by IB. (**G and H**) HA-VP4 was transfected into WT or RNF31-KO HEK293T cells for 24 h. Cells were then treated with CHX (50 μg/mL) and collected at the indicated time points for IB analysis (G). Band intensities were quantified using ImageJ software and normalized to ACTB as an internal control (H). (**I**) HEK293T cells were co-transfected with Flag-RNF31 and HA-VP4 for 24 h and subsequently treated with DMSO or Bortezomib for 12 h, followed by IB analysis of cell lysates.
(TIF)

S6 Fig The RNF31 enzyme activity inhibitor HOIPIN-8 suppresses RNF31 antiviral activity. (**A**) HEK293T cells were co-transfected with HA-VP4, MYC-Ub, and Flag-RNF31 and treated with DMSO or HOIPIN-8 (20 nM). In the presence of MG132 (10 μM), cell lysates were subjected to co-IP using anti-HA antibodies, followed by IB analysis. (**B-D**) RD cells were treated with DMSO or HOIPIN-8 and subsequently infected with EV-A71. Following infection, cell lysates were collected for IB analysis (B), and culture supernatants were harvested for RT-qPCR (C) analysis and TCID₅₀ assays (D). Statistical significance is indicated as follows: *P < 0.05; **P < 0.01; ***P < 0.001; ****P < 0.0001; ns, not significant.
(TIF)

S7 Fig Cleavage of RNF31 eliminates its antiviral activity. (**A and B**) RD cells were transfected with a control plasmid, Flag-RNF31, Flag-RNF31-N, or Flag-RNF31-C and subsequently infected with EV-A71 at 24 h post-transfection. After 48 h of infection, the mRNA expression levels of IFN-β (A) and ISG15 (B) were quantified by RT-qPCR. Statistical significance is indicated as follows: *P < 0.05; **P < 0.01; ***P < 0.001; ****P < 0.0001; ns, not significant.
(TIF)

S8 Fig RNF31 degrades VP4 of various enteroviruses. (**A-C**) HEK293T cells were co-transfected with Flag-RNF31 and VP4 from CV-A16 (A), CV-B3 (B), or EV-D68 (C). Cell lysates were collected 48 h post-transfection and analyzed by IB.
(TIF)

S1 Table Primers used for plasmid construction and RT-qPCR in this study. (DOCX)

S2 Table Primers used for RT-qPCR in this study. (DOCX)

S3 Table Cleavage sequences of viral and host substrates by EV-A71 3C pro. (DOCX)

S1 Raw Gel The raw data of western blot in this study. (PDF)

S1 Data Mass spectrometry data for EV-A71 3C and EV-A71 3C^H40A,C147A^. (XLS)

S2 Data The raw data supporting each of the manuscript Figures are contained in this Excel file. (XLSX)
