# Supplementary figures and images for: RNF31 restricts EV-A71 replication through innate immune activation and VP4 degradation, and is antagonized by viral 3C proteases
Source: PLoS Pathog. 2026 Jul 2;22(7):e1014415. doi: 10.1371/journal.ppat.1014415 (PMC13345468; doi:10.1371/journal.ppat.1014415)

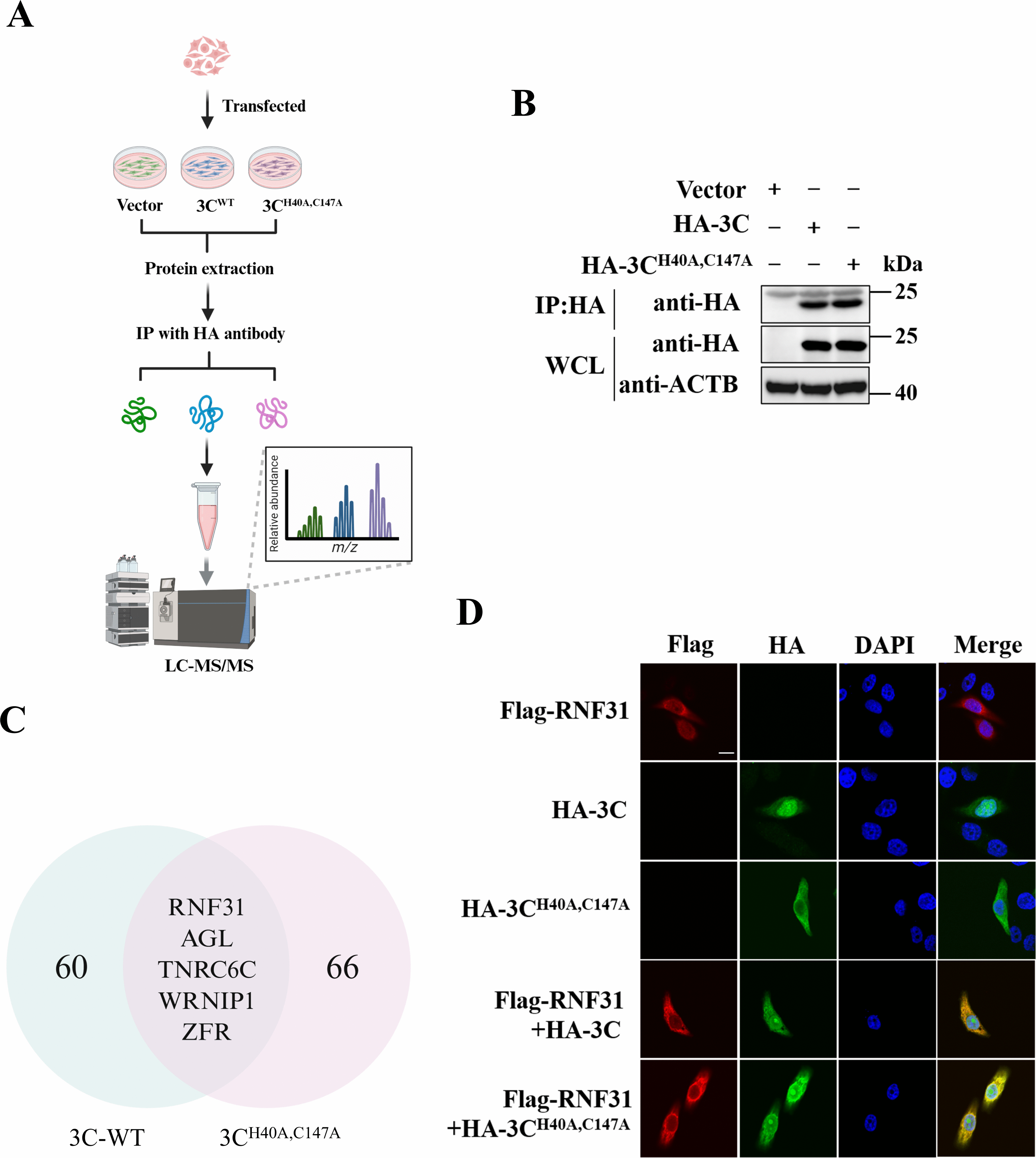

Supplement: S1 Fig — (A) Schematic overview of the LC-MS/MS-based experimental workflow. Samples were prepared for LC-MS/MS analysis, and proteins interacting with 3C or the catalytically inactive mutant 3CH40A,C147A were enriched by co-IP. Created in BioRender. Gao, Y. (2026). https://BioRender.com/mv61d2l. (B) HEK293T cells were transfected with a control plasmid, HA-3C, or HA-3CH40A,C147A for 36 h and subsequently treated with MG132 (10 μM) for 12 h. Cell lysates were incubated with anti-HA antibody-conjugated protein G agarose beads, followed by IB using anti-HA antibodies. The enriched protein complexes were then subjected to MS analysis. (C) LC-MS/MS analysis identified proteins interacting with 3Cpro and preferentially enriched in 3CproH40A,C147A. Candidates were selected based on a binding enrichment ratio >5 for 3Cpro versus empty vector (left) and >2 for 3CproH40A,C147A versus 3Cpro (right), yielding five candidate proteins. (D) HeLa cells were co-transfected with Flag-RNF31 and either HA-3C or HA-3CH40A,C147A. Co-localization of RNF31 with 3C or 3CH40A,C147A was examined by confocal laser scanning microscopy. Nuclei were counterstained with DAPI. Scale bar, 10 μm. (TIF) [file ppat.1014415.s001.tif]

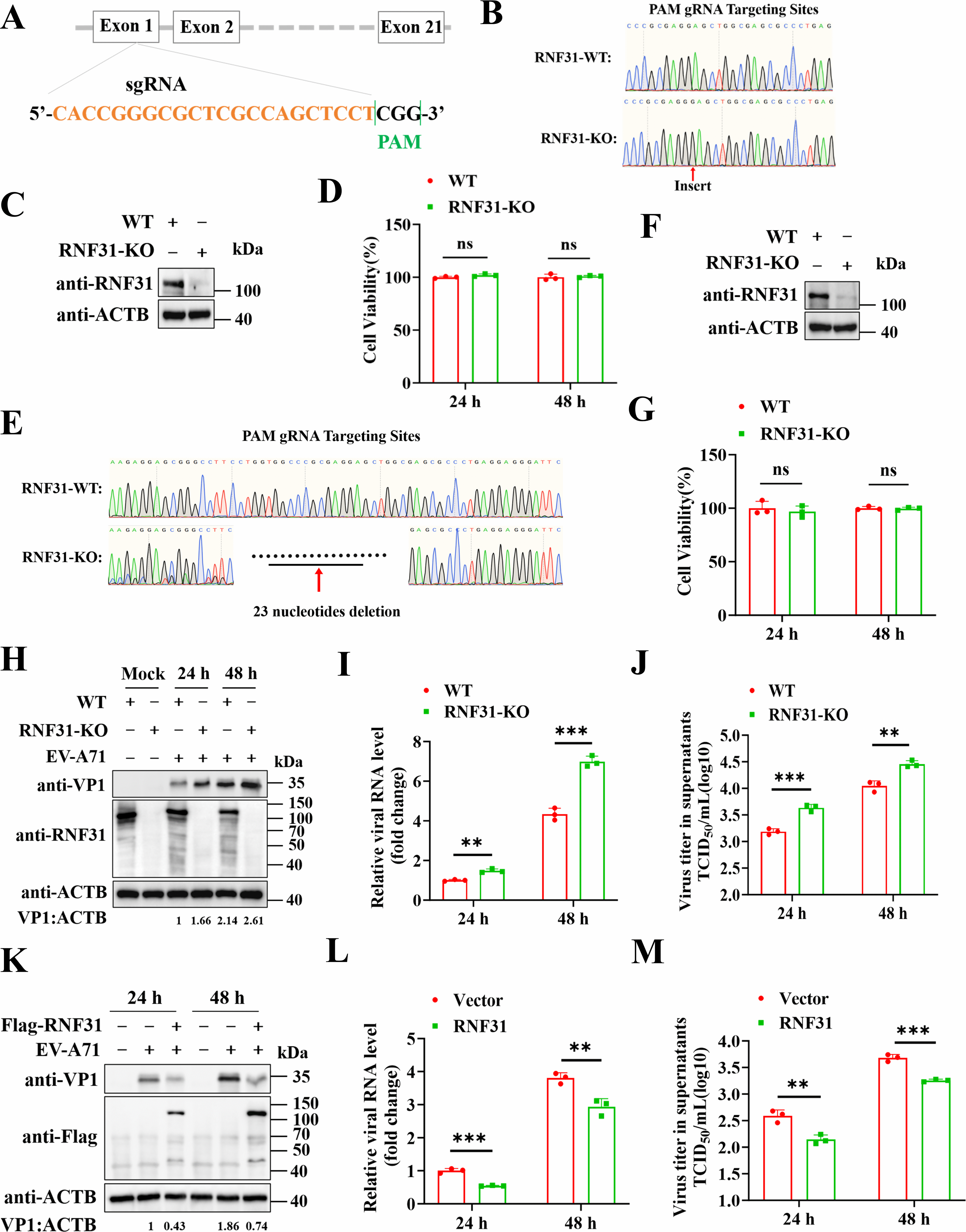

Supplement: S2 Fig — (A) Schematic illustration of the CRISPR-Cas9 strategy used to generate RNF31-KO cells. (B and C) Validation of RNF31 knockout in RD cells by genomic DNA sequencing (B) and IB analysis (C). (D) Cell viability of WT and RNF31-KO RD cell lines. (E and F) Validation of RNF31 knockout in HEK293T cells by genomic DNA sequencing (E) and IB analysis (F). (G) Cell viability of WT and RNF31-KO HEK293T cell lines. (H-J) WT and RNF31-KO HEK293T cells were infected with EV-A71 for 24 h or 48 h. Following infection, cell lysates were analyzed by IB (H), and culture supernatants were subjected to RT-qPCR (I) and TCID₅₀ assays (J). (K-M) HEK293T cells transfected with Flag-RNF31 or control plasmids were infected with EV-A71 for 24 h or 48 h. After infection, cell lysates were analyzed by IB (K), and supernatants were collected for RT-qPCR (L) and TCID₅₀ assays (M). Data are presented as the mean ± SEM from three independent experiments. Statistical significance is indicated as follows: *P < 0.05; **P < 0.01; ***P < 0.001; ****P < 0.0001; ns, not significant. (TIF) [file ppat.1014415.s002.tif]

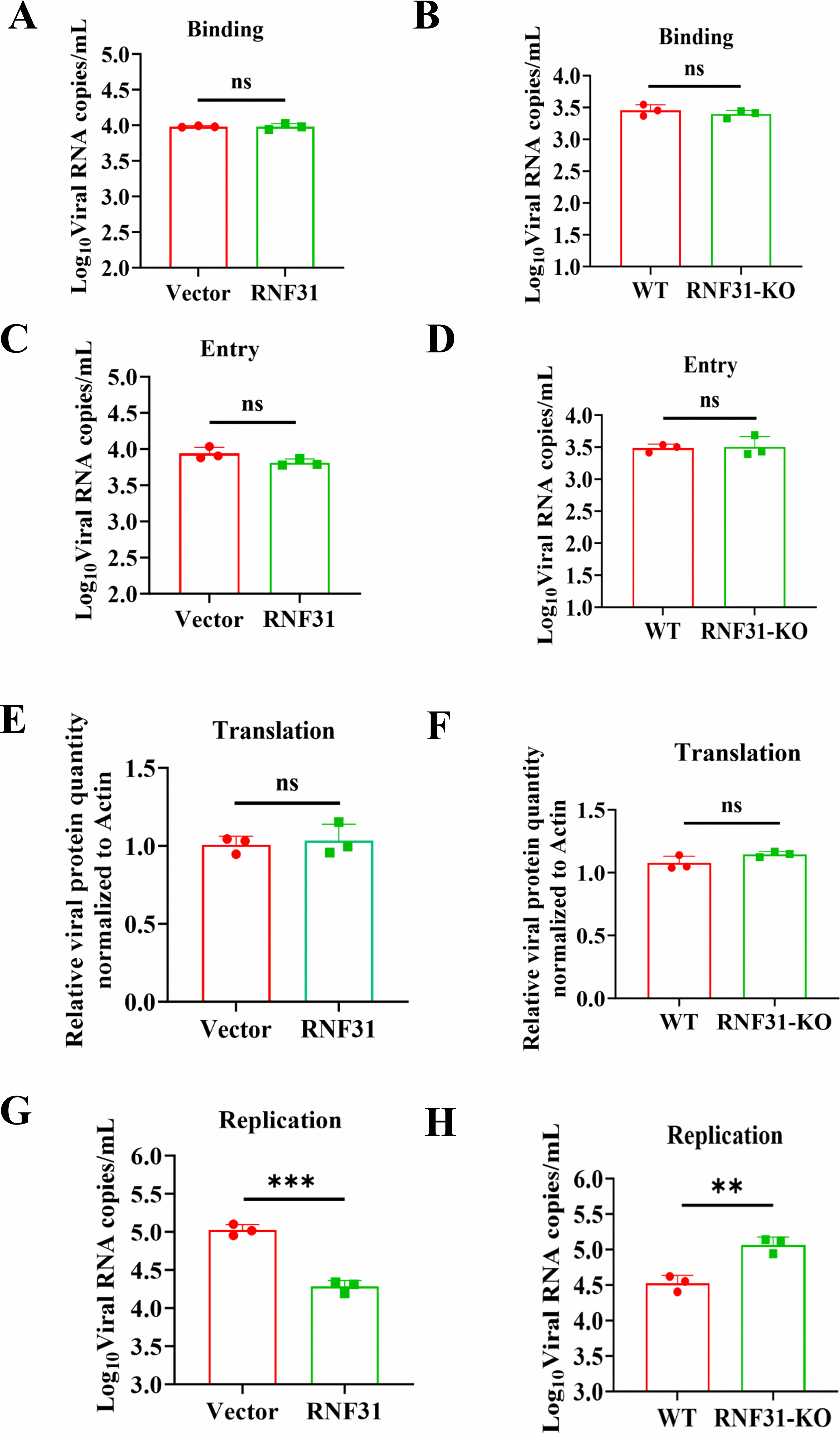

Supplement: S3 Fig — (A and B) WT (A) or RNF31-KO (B) RD cells were transfected with a control plasmid or Flag-RNF31. At 24 h post-transfection, cells were infected with EV-A71 at 4°C. After 1 h of adsorption, cells were washed three times with PBS, harvested, and subjected to RT-qPCR to quantify viral RNA copy numbers, thereby assessing the effect of RNF31 on EV-A71 attachment.Y-axis indicates viral RNA levels expressed as log10 copies/mL. (C and D) WT (C) or RNF31-KO (D) RD cells were transfected with a control plasmid or Flag-RNF31 for 24 h and infected with EV-A71 at 4°C. After 1 h of adsorption, cells were washed three times with PBS, replaced with maintenance medium, and incubated at 37°C in a 5% CO₂ incubator to allow viral entry. Following 1 h of incubation, cells were washed twice with pre-chilled PBS, once with pre-chilled alkaline high-salt solution, and twice again with pre-chilled PBS. Cells were then collected for RT-qPCR analysis to quantify viral RNA copy numbers and evaluate the effect of RNF31 on EV-A71 entry. (E and F) WT (E) or RNF31-KO (F) RD cells were transfected with a control plasmid or Flag-RNF31 and infected with EV-A71 as described above. Cells were harvested for IB at 4 hpi to determine viral protein levels, thereby assessing the effect of RNF31 on EV-A71 translation. Relative viral protein levels were quantified by densitometric analysis of IB results and normalized to ACTB. (G and H) WT (G) or RNF31-KO (H) RD cells were transfected with a control plasmid or Flag-RNF31 and infected with EV-A71 as described above. Cells were harvested at 8 hpi for RT-qPCR to quantify viral RNA copy numbers, thereby evaluating the effect of RNF31 on EV-A71 replication. Statistical significance is indicated as follows: *P < 0.05; **P < 0.01; ***P < 0.001; ****P < 0.0001; ns, not significant. (TIF) [file ppat.1014415.s003.tif]

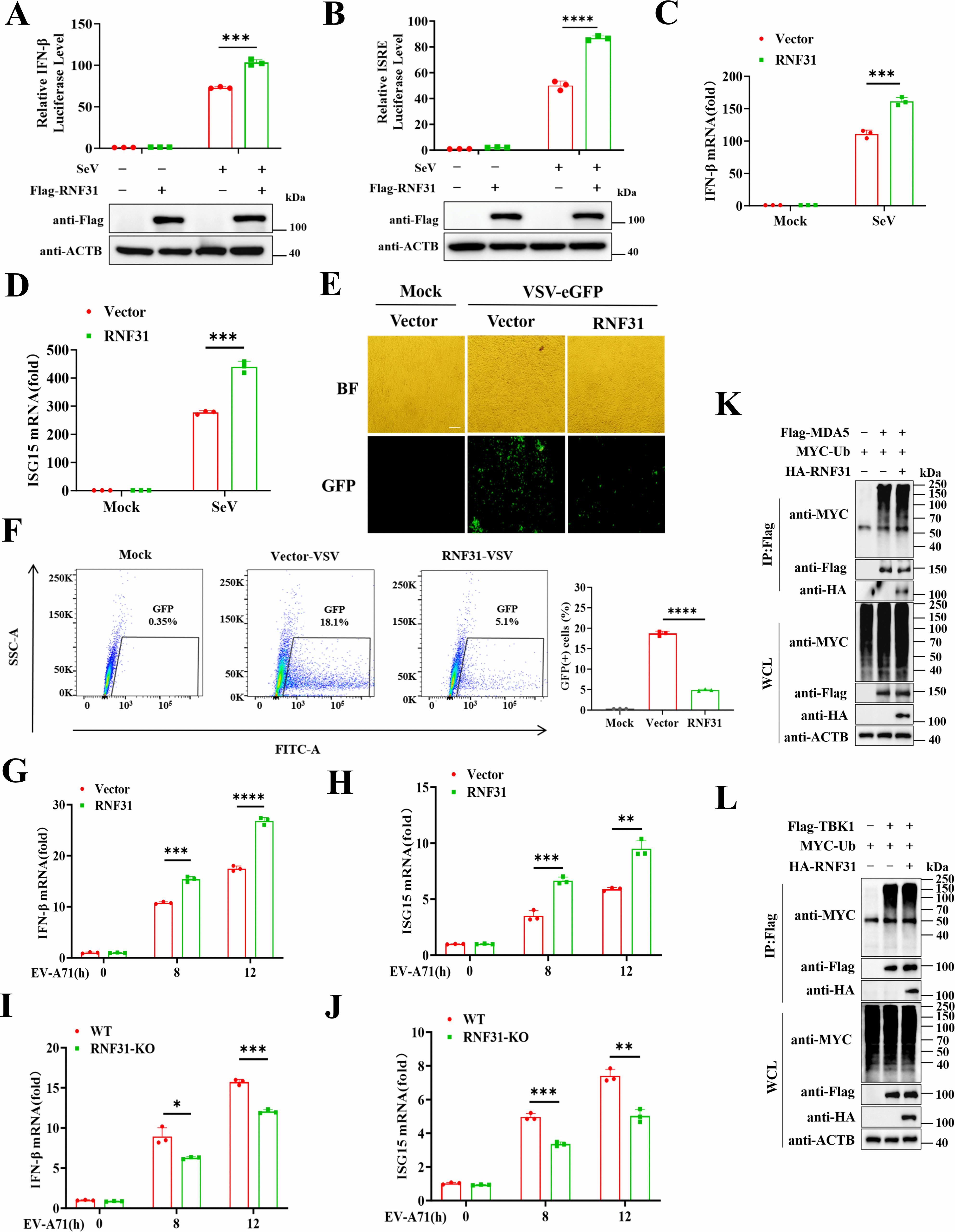

Supplement: S4 Fig — (A and B) HEK293T cells were co-transfected with Flag-RNF31, Renilla luciferase, and IFN-β-Luc or ISRE-Luc reporter plasmids and subsequently infected with SeV at 24 h post-transfection. After 12 h of infection, IFN-β (A) and ISRE (B) promoter activities were measured using a dual-luciferase reporter assay. (C and D) HEK293T cells were transfected with Flag-RNF31 and subsequently infected with SeV at 24 h post-transfection. After 12 h of infection, the mRNA expression levels of IFN-β (C) and ISG15 (D) were quantified by RT-qPCR. (E and F) HEK293T cells were transfected with Flag-RNF31 or an empty control plasmid for 24 h and then infected with VSV-GFP for 12 h. Following infection, cells were fixed and examined by confocal microscopy (E) or quantified by flow cytometry (F). Scale bar, 200 μm. (G and H) RD cells were transfected with Flag-RNF31 and infected with EV-A71 at 24 h post-transfection. Following infection, the mRNA expression levels of IFN-β (G) and ISG15 (H) were measured by RT-qPCR. (I and J) WT or RNF31-KO RD cells transfected with Flag-RNF31 and infected with EV-A71 at 24 h post-transfection. Following infection, the mRNA expression levels of IFN-β (I) and ISG15 (J) were measured by RT-qPCR. (K) Co-IP assays were performed using lysates from HEK293T cells co-transfected with HA-RNF31, Flag-MDA5, and MYC-Ub in the presence of MG132 (10 μM). IP was carried out with anti-Flag antibodies, followed by IB analysis. (L) Co-IP analysis was conducted using lysates from HEK293T cells co-transfected with HA-RNF31, Flag-TBK1, and MYC-Ub in the presence of MG132 (10 μM), followed by IP with anti-FLAG antibodies and IB analysis. Statistical significance is indicated as follows: *P < 0.05; **P < 0.01; ***P < 0.001; ****P < 0.0001; ns, not significant. (TIF) [file ppat.1014415.s004.tif]

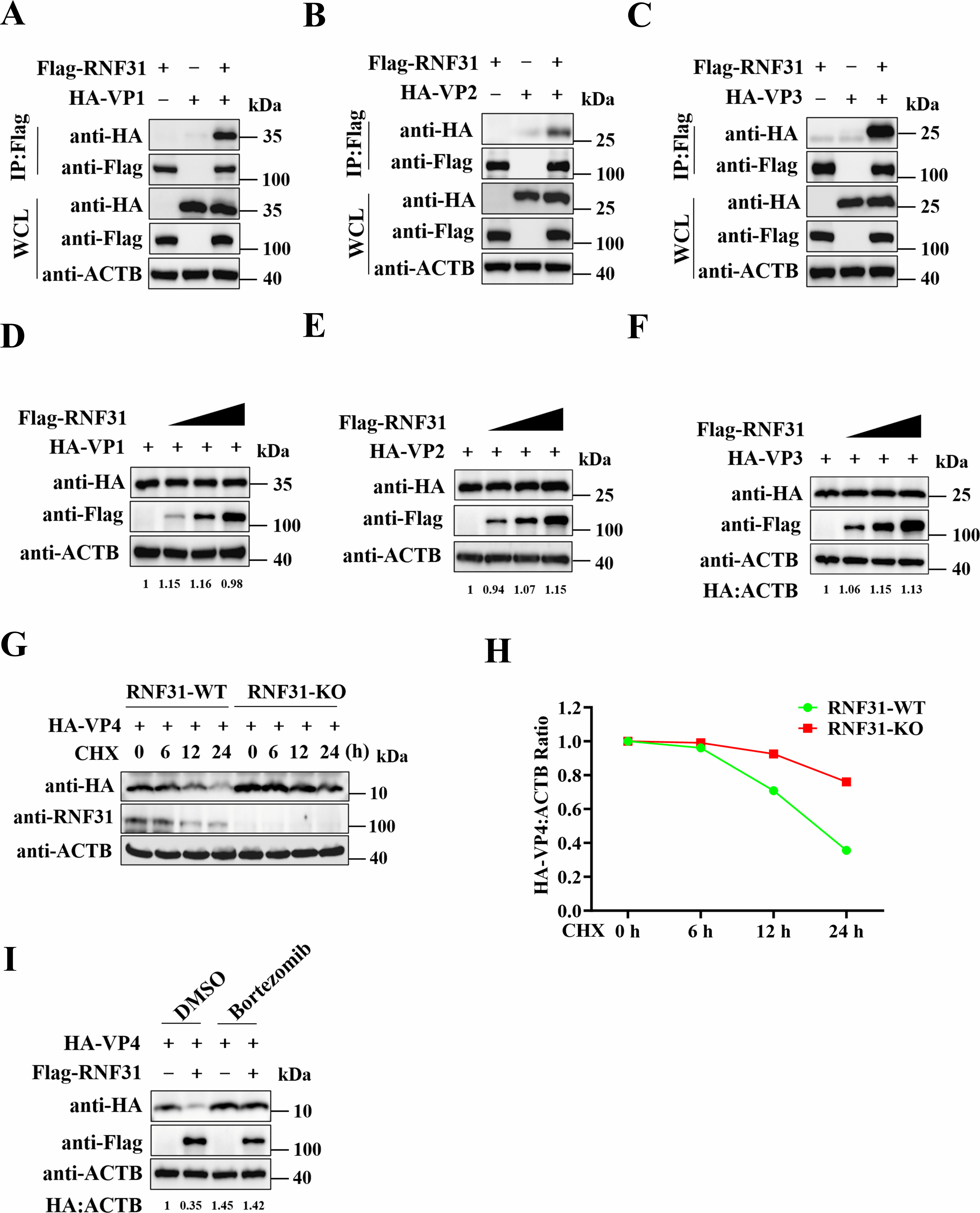

Supplement: S5 Fig — (A-C) HEK293T cells were co-transfected with Flag-RNF31 and either HA-VP1 (A), HA-VP2 (B), or HA-VP3 (C) for 36 h and subsequently treated with MG132 (10 μM). After an additional 12 h, cell lysates were subjected to co-IP using anti-Flag antibodies, followed by IB analysis. (D-F) HEK293T cells were co-transfected with increasing amounts of Flag-RNF31 together with HA-VP1 (D), HA-VP2 (E), or HA-VP3 (F). Cell lysates were harvested at 48 h post-transfection and analyzed by IB. (G and H) HA-VP4 was transfected into WT or RNF31-KO HEK293T cells for 24 h. Cells were then treated with CHX (50 μg/mL) and collected at the indicated time points for IB analysis (G). Band intensities were quantified using ImageJ software and normalized to ACTB as an internal control (H). (I) HEK293T cells were co-transfected with Flag-RNF31 and HA-VP4 for 24 h and subsequently treated with DMSO or Bortezomib for 12 h, followed by IB analysis of cell lysates. (TIF) [file ppat.1014415.s005.tif]

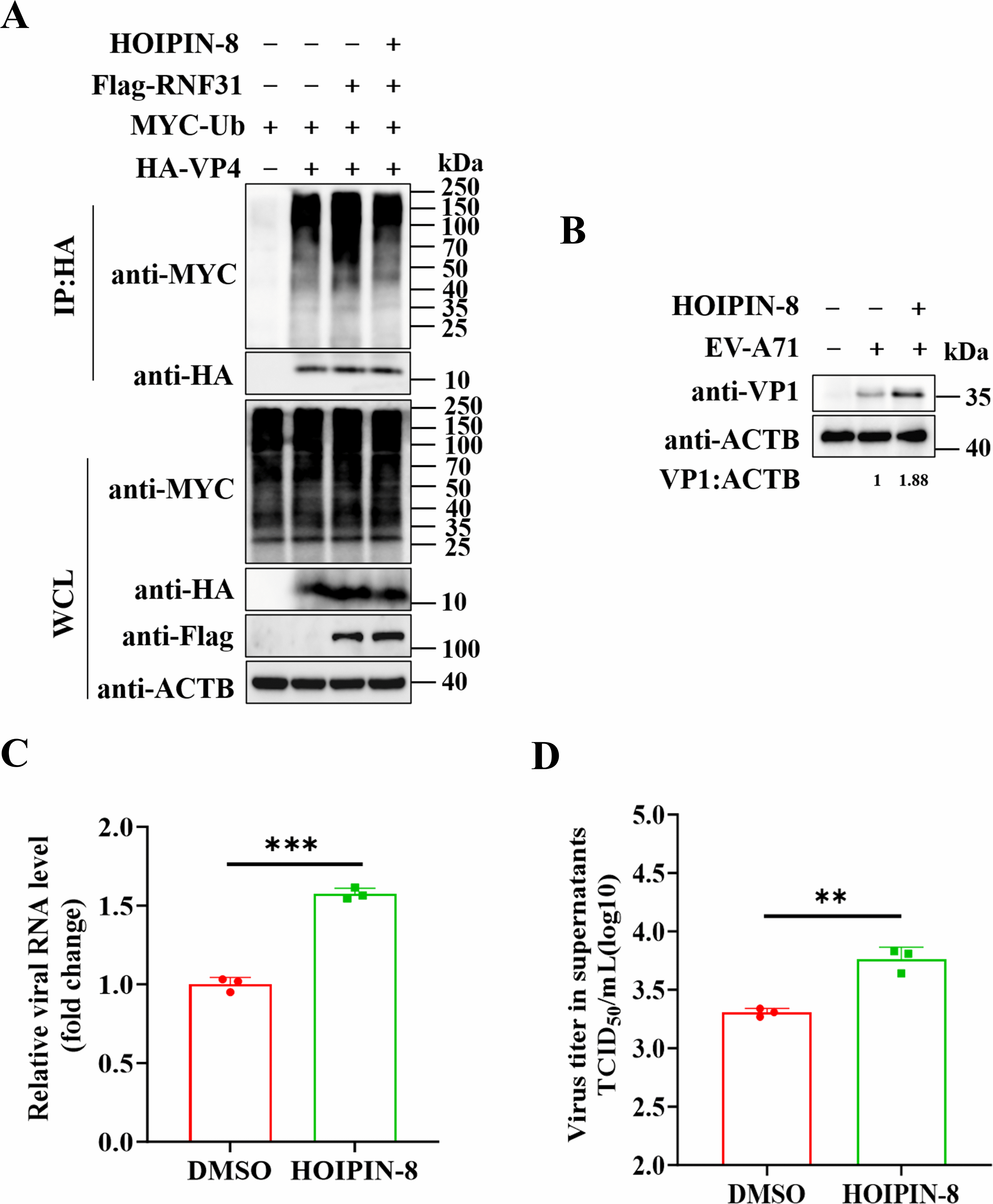

Supplement: S6 Fig — (A) HEK293T cells were co-transfected with HA-VP4, MYC-Ub, and Flag-RNF31 and treated with DMSO or HOIPIN-8 (20 nM). In the presence of MG132 (10 μM), cell lysates were subjected to co-IP using anti-HA antibodies, followed by IB analysis. (B-D) RD cells were treated with DMSO or HOIPIN-8 and subsequently infected with EV-A71. Following infection, cell lysates were collected for IB analysis (B), and culture supernatants were harvested for RT-qPCR (C) analysis and TCID₅₀ assays (D). Statistical significance is indicated as follows: *P < 0.05; **P < 0.01; ***P < 0.001; ****P < 0.0001; ns, not significant. (TIF) [file ppat.1014415.s006.tif]

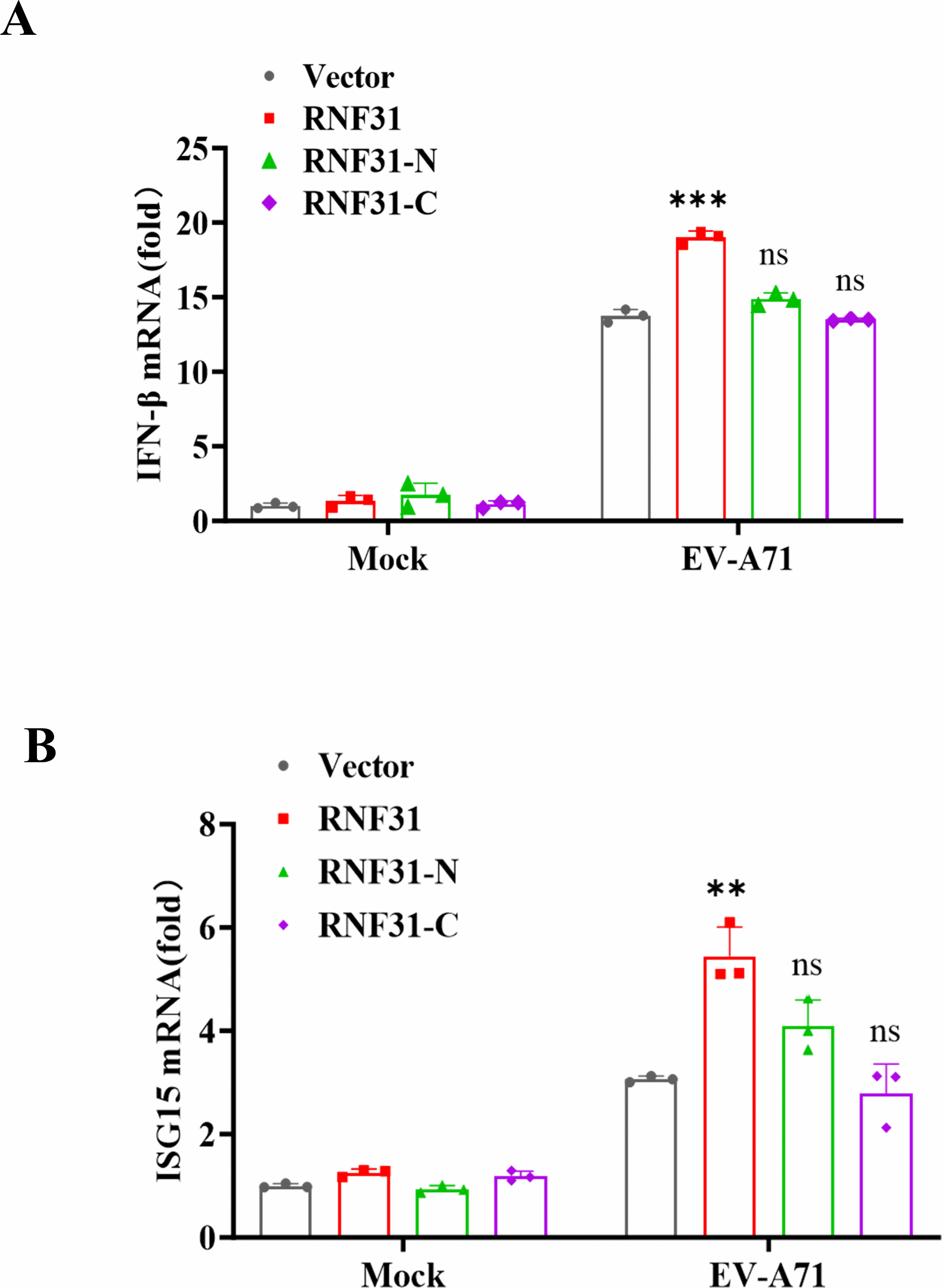

Supplement: S7 Fig — (A and B) RD cells were transfected with a control plasmid, Flag-RNF31, Flag-RNF31-N, or Flag-RNF31-C and subsequently infected with EV-A71 at 24 h post-transfection. After 48 h of infection, the mRNA expression levels of IFN-β (A) and ISG15 (B) were quantified by RT-qPCR. Statistical significance is indicated as follows: *P < 0.05; **P < 0.01; ***P < 0.001; ****P < 0.0001; ns, not significant. (TIF) [file ppat.1014415.s007.tif]

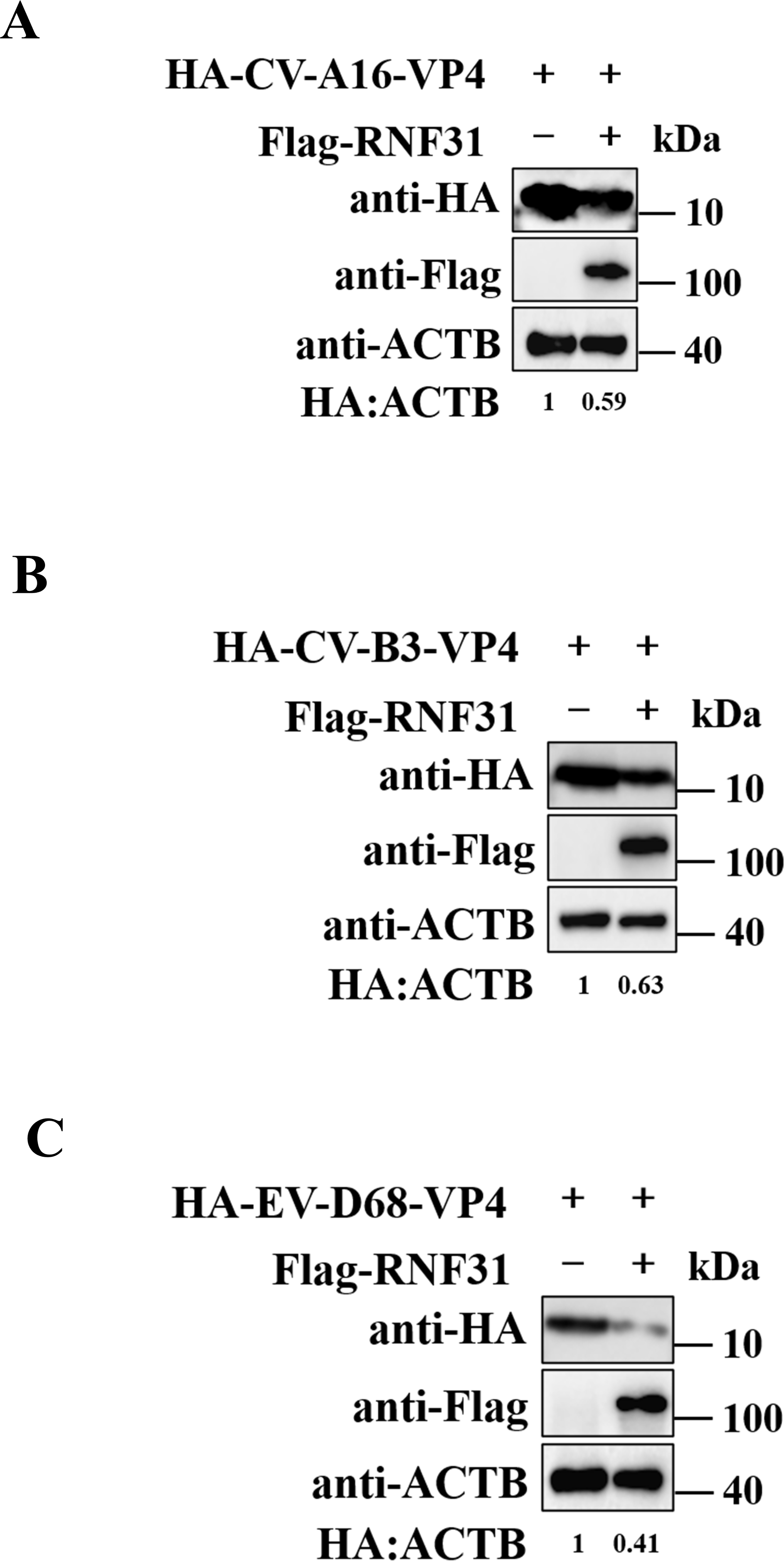

Supplement: S8 Fig — (A-C) HEK293T cells were co-transfected with Flag-RNF31 and VP4 from CV-A16 (A), CV-B3 (B), or EV-D68 (C). Cell lysates were collected 48 h post-transfection and analyzed by IB. (TIF) [file ppat.1014415.s008.tif]
