# Supplementary material for: RNF31 restricts EV-A71 replication through innate immune activation and VP4 degradation, and is antagonized by viral 3C proteases
Source: PLoS Pathog. 2026 Jul 2;22(7):e1014415. doi: 10.1371/journal.ppat.1014415 (PMC13345468; doi:10.1371/journal.ppat.1014415)
Supplement: S1 Table — (DOCX) [file ppat.1014415.s009.docx]

**S1 Table.** **Primers used for gene cloning in this study.**

| Name | Forward sequence (5ʹ-3ʹ) | Reverse sequence (5ʹ-3ʹ) |
| --- | --- | --- |
| *Flag-RNF31-VR1012* | AGATATCGCGGCCGCTCTAGAATGGATTACAAGGATGACGAC | AGGCACAGCAGATCTGGATCCCTACTTCCGCCTGCGGGG |
| *RNF31-Q340A* | CTGAGGGTCCCGCAGGAACTGGAGG | TGCGGGACCCTCAGTTCC |
| *RNF31-Q357A* | GTGGGCCTGCGCGAGCTGTACCTTTG | CGCGCAGGCCCACCGACCCCG |
| *RNF31-Q400A* | TGCAACCACTTCAGGCGGGGGATGCTT | CGCCTGAAGTGGTTGCAGG |
| *RNF31-Q409A* | GGCCTCTGCCGCGAGTCAAGTCTGG | CGCGGCAGAGGCCAGCAAAGCA |
| *RNF31-C885S* | CCGAGGAGGCAGCATGCACTTTC | CTGCCTCCTCGGGCCAGGGCG |
| *EV-A71-3C-H40A* | GTCCTCCCACGCGCCTCACAACCTGGC | GAGGCGCGTGGGAGGACTGCTAAGCGATC |
| *EV-A71-3C-C147A* | AAAGCAGGACAGGCTGGAGGAGTGGTG | CCAGCCTGTCCTGCTTTAGTAGGAAAG |
| *EV-D68 -3C-H40A* | CAGTCATTCCAACAGCTGCATCTGTT | AGCTGTTGGAATGACTGCCACTCTATC |
| *EV-D68-3C-C147A* | AGAGCTGGTCAGGCTGGTGGTGTGG | AGCCTGACCAGCTCTTGTTGGAAA |
| *CV-A16-3C-H40A* | ATTTTGCCGCGCGCCTCGCAACCAG | GGCGCGCGGCAAAATGGCTAGGCG |
| *CV-A16-3C-C147A* | AAGGCAGGGCAGGCTGGAGGTGTGG | AGCCTGCCCTGCCTTTGTGGGGAA |
| *CV-B3-3C-H40A* | TTTTGCCACGCGCCGCCAAACCTG | GGCGCGTGGCAAAACGGCCCACC |
| *CV-B3-3C-C147A* | AGCAGGCCAGGCTGGTGGAGTGC | AGCCTGGCCTGCTCTTGTGGGG |
| *EV-A71-VP4-K33R* | CACCATTAATTACTACAGAGACTCCTATGCTG | TCTGTAGTAATTAATGGTGGTGTAGTTTA |
| *EV-A71-VP4-K42R* | CACAGCAGGCAGACAGAGTCTCAAGC | TCTGCCTGCTGTGGCAGCATAG |
| *EV-A71-VP4-K46R* | CAAACAGAGTCTCAGGCAGGATCCAGAC | CCTGAGACTCTGTTTGCCTGCTGTG |
| *EV-A71-VP4-K51R* | CAGGATCCAGACAGGTTTGCAAATCCT | CCTGTCTGGATCCTGCTTGAGACT |
| *EV-A71-VP4-K57R* | TTTGCAAATCCTGTTAGAGACATCTTCACTGA | TCTAACAGGATTTGCAAACTTGTCTGG |
| *RNF31-N* | GACGACGATAAGGAATTCATGCCGGGGGAGGAAGAGGAG | GATCTGCTAGCTCGAGCTGCTGAAGGGGTTGCAGGCAAA |
| *RNF31-C* | GACGACGATAAGGAATTCGGGGATGCTTTGCTGGCCTCTGC | TAAGATCTGCTAGCTCGAGCTACTTCCGCCTGCGGGGGAT |
