# Supplementary material for: RNF31 restricts EV-A71 replication through innate immune activation and VP4 degradation, and is antagonized by viral 3C proteases
Source: PLoS Pathog. 2026 Jul 2;22(7):e1014415. doi: 10.1371/journal.ppat.1014415 (PMC13345468; doi:10.1371/journal.ppat.1014415)
Supplement: S2 Table — (DOCX) [file ppat.1014415.s010.docx]

**S2 Table.** **Primers used for RT-qPCR in this study.**

| Name | Forward sequence (5ʹ-3ʹ) | Reverse sequence (5ʹ-3ʹ) |
| --- | --- | --- |
| *RNF31-RT* | GAGCCCCGAAACTACCTCAAC | CTTGACACCACGCCAGTACC |
| *IFN-β-RT* | TCTACAGAGCCTTGCCTGCAT | TGTCGGTGTCCAAAAGGATGT |
| *ISG15-RT* | CGCAGATCACCCAGAAGATCG | TTCGTCGCATTTGTCCACCA |
| *EV-A71-VP1-RT* | AGCACCCACAGGCCAGAACAC | ATCCCGCCCTACTGAAGAAACTA |
| *EV-A71-VP4-RT* | TTCTCAGGTGTCTACACAGC | TGCCATTTCAGTGAAGATGT |
| *EV-D68-VP1-RT* | GGAGAAAGCTGGAACTGTTC | GGGCACCAGTAGGTACAAAC |
| *CV-A16-VP1-RT* | CATGCAGCGCTTGTGCTT | CACACAATTCCCCCGTCTTAC |
| *CV-B3-VP1-RT* | ATGAGACCAGGCTGAATGC | TACTGGTTCTGTGAACTTGC |
