# Supplementary material for: RNF31 restricts EV-A71 replication through innate immune activation and VP4 degradation, and is antagonized by viral 3C proteases
Source: PLoS Pathog. 2026 Jul 2;22(7):e1014415. doi: 10.1371/journal.ppat.1014415 (PMC13345468; doi:10.1371/journal.ppat.1014415)
Supplement: S3 Table — (DOCX) [file ppat.1014415.s011.docx]

**S3 Table.** **Cleavage sequences of viral and host substrates by EV-A71 3C pro.**

| Gene name | Cleavage sites | Cleavage motifs |
| --- | --- | --- |
| *EV-A71-VP3/VP1* | 561...570 | SKFHQGALLV |
| *EV-A71-2A-2BC* | 1008...1017 | EAMEQ GVSDY |
| *EV-A71-2BC-3AB* | 1436...1445 | EALFQGPPKF |
| *EV-A71-3AB-3CD* | 1544...1553 | TATVQGPSLD |
| *EV-A71-2B-2C* | 1107...1116 | MAQKQSASWL |
| *EV-A71-3A-VPg* | 1522...1531 | FAGFQGAYSG |
| *EV-A71-3C-3D* | 1727...1736 | FASEQGEIQW |
| *TRIF* | 308...317 | SAGPQSLPLP |
| *TAK1* | 356...365 | TPQMQ SRSSS |
| *TAB1* | 410...419 | VMPSQGQLVN |
|  | 447...456 | TLTLQSTTTH |
| *TAB2* | 109...118 | LQGGQSNSEL |
| *TAB3* | 169...178 | PSAMQGPSPP |
|  | 339...348 | SYQKQGSHSV |
| *IRF7* | 185...194 | QAVQQSCLAD |
| *NLRP3* | 221...230 | TVVFQGAAGI |
| *PINX1* | 46...55 | GAQEQGATDH |
| *TRAF3IP3* | 83...92 | QSKLQGADSR |
| *OAS3* | 978...987 | YAWEQGGKDS |
| *PMLIV* | 426...435 | KAQVQALGLA |
|  | 440...449 | MAVVQSVPGA |
| *GSDMD* | 189...198 | ATCLQGEGQG |
