# Supplementary material for: RNF31 restricts EV-A71 replication through innate immune activation and VP4 degradation, and is antagonized by viral 3C proteases
Source: PLoS Pathog. 2026 Jul 2;22(7):e1014415. doi: 10.1371/journal.ppat.1014415 (PMC13345468; doi:10.1371/journal.ppat.1014415)
Supplement: S1 Raw Gel — (PDF) [file ppat.1014415.s012.pdf]

Figure 1A

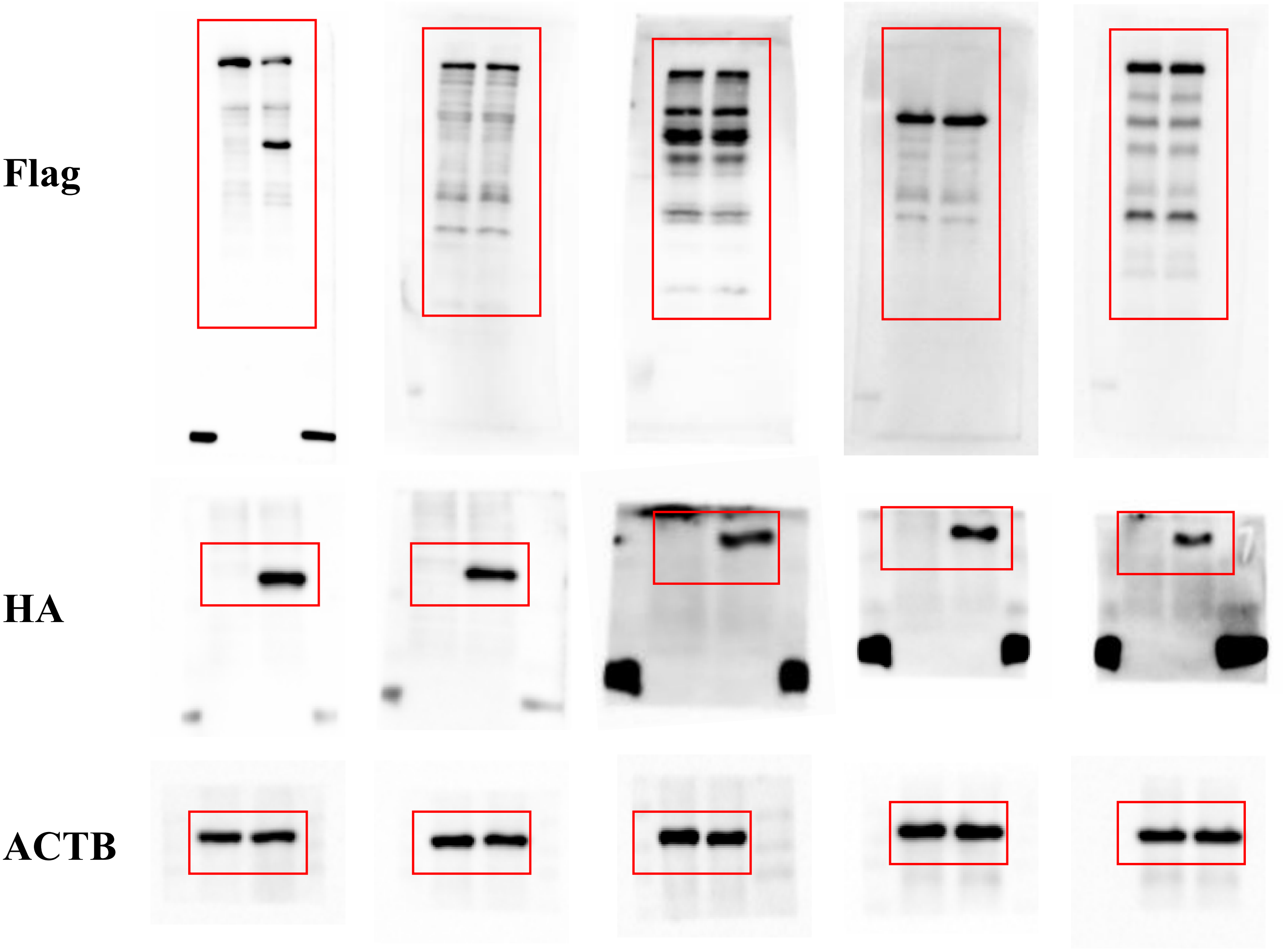

Figure 1B

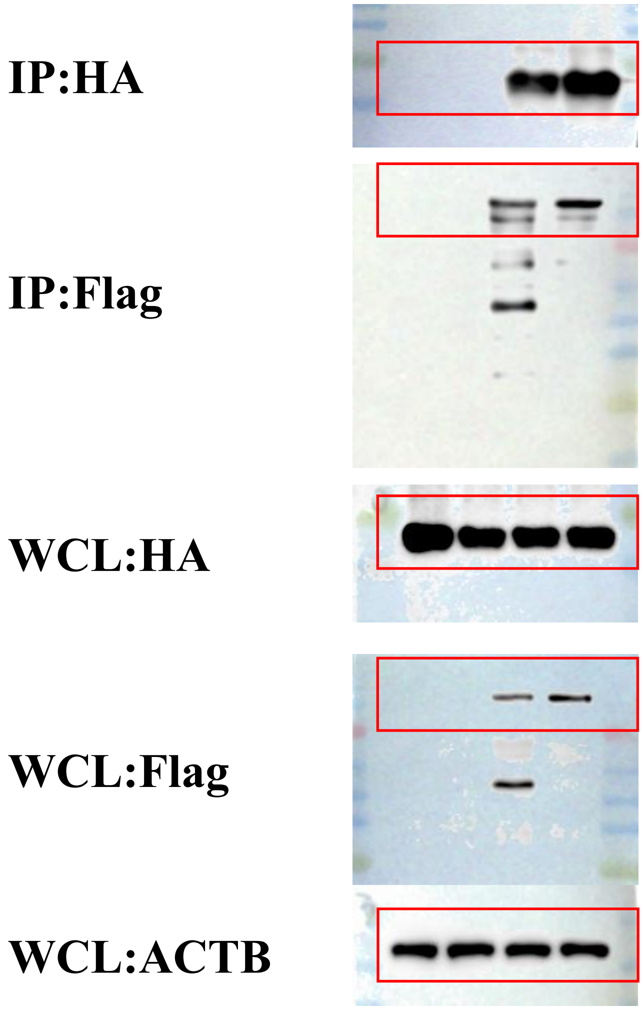

Figure 1C

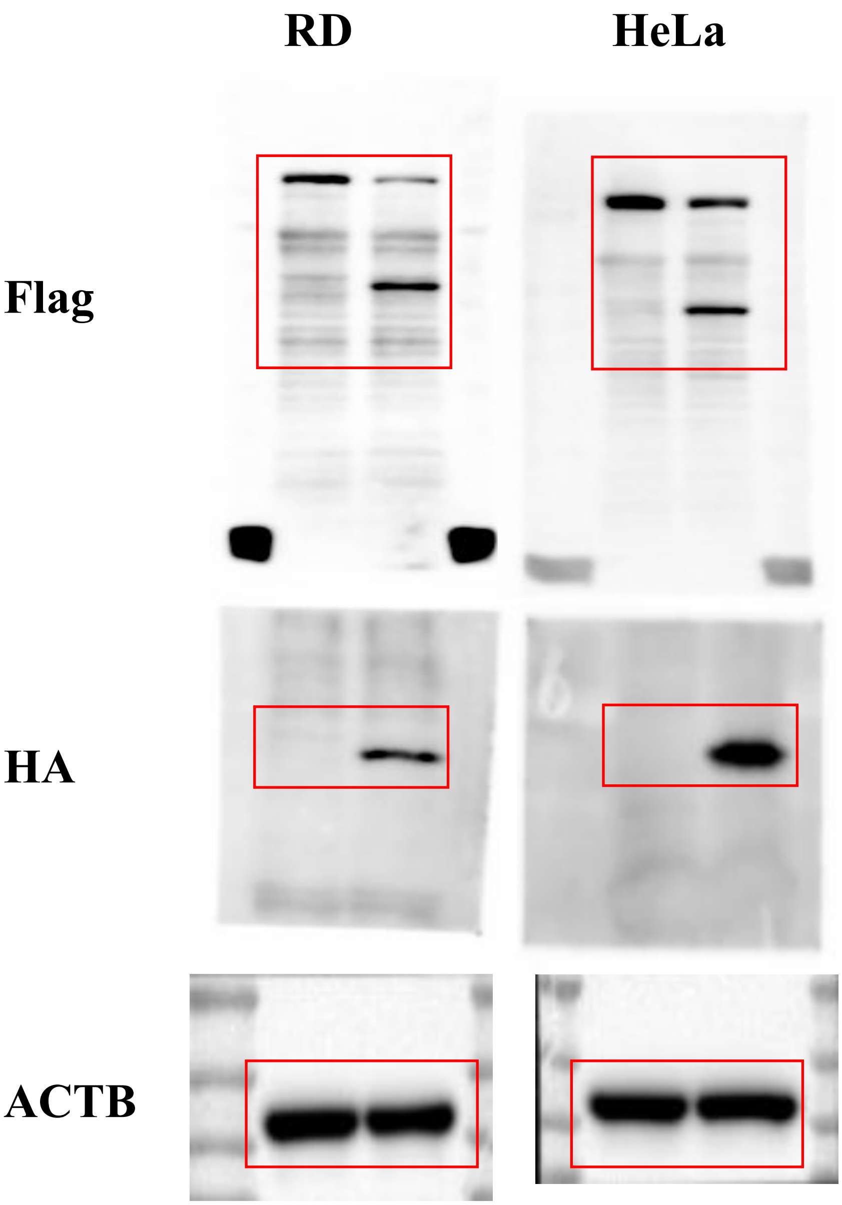

Figure 1D

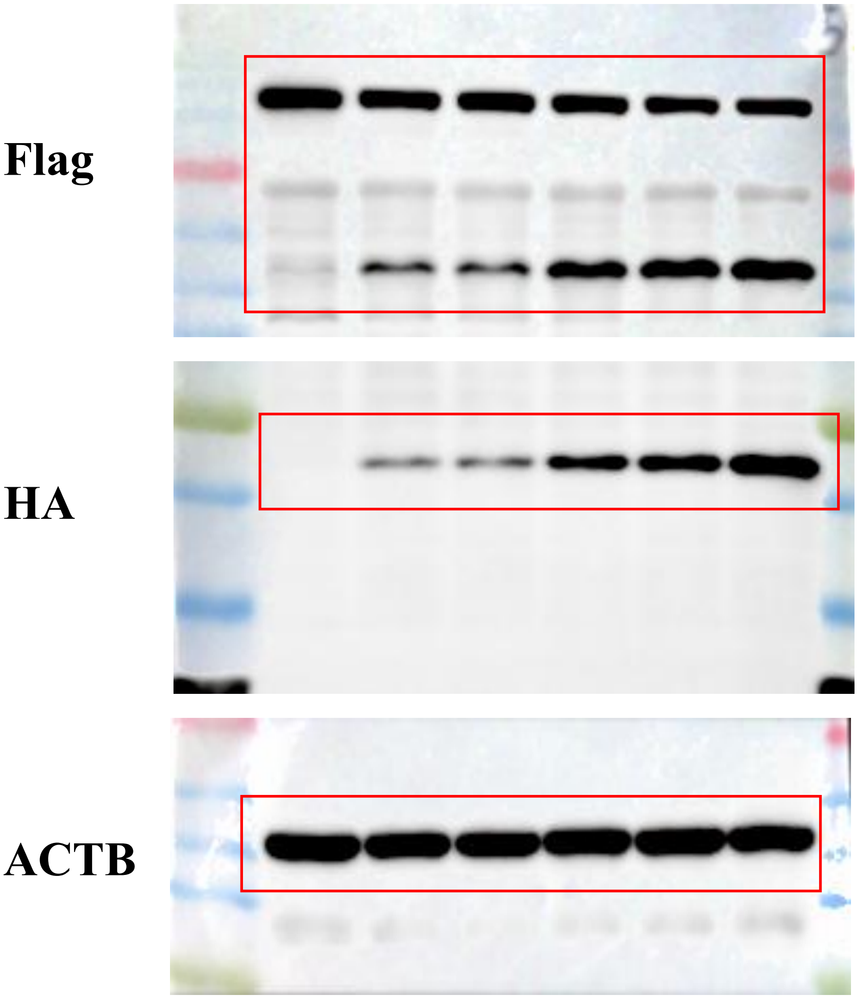

Figure 1F

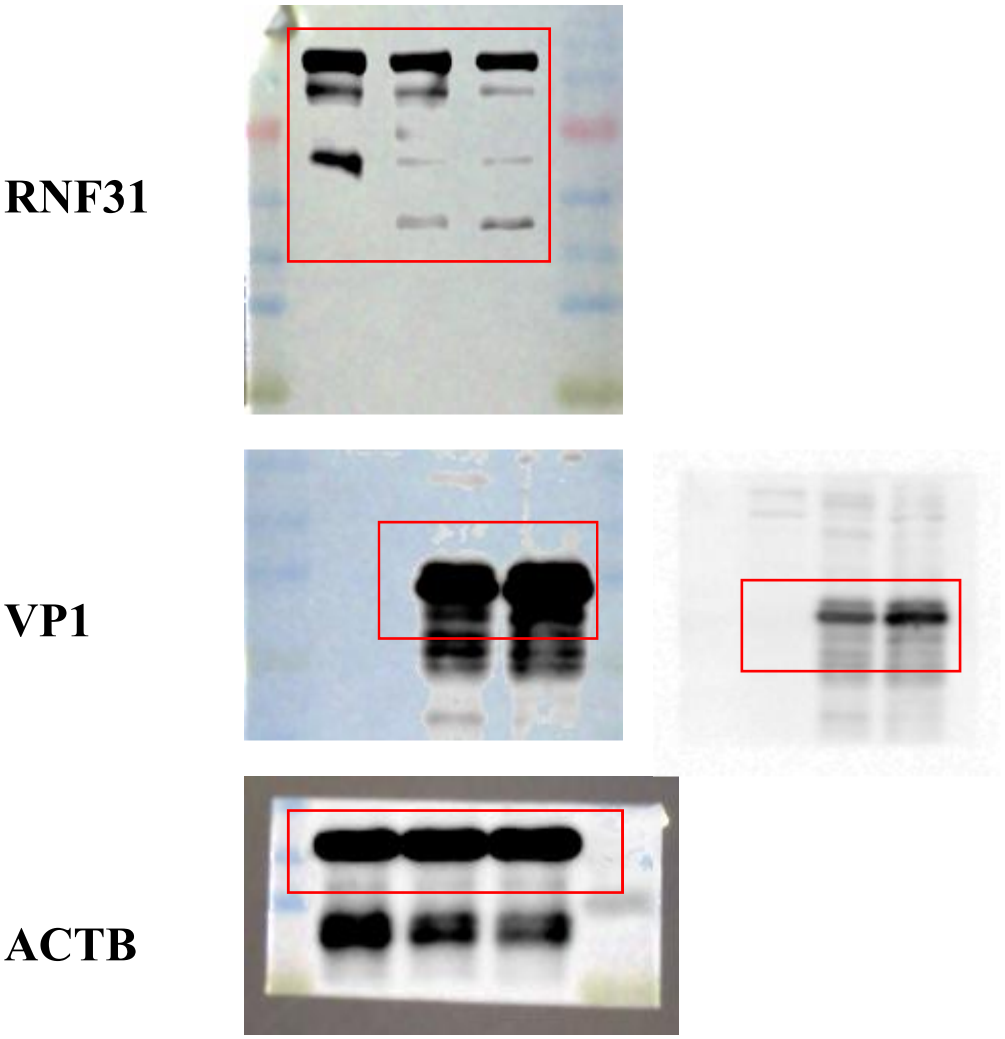

Figure 1G

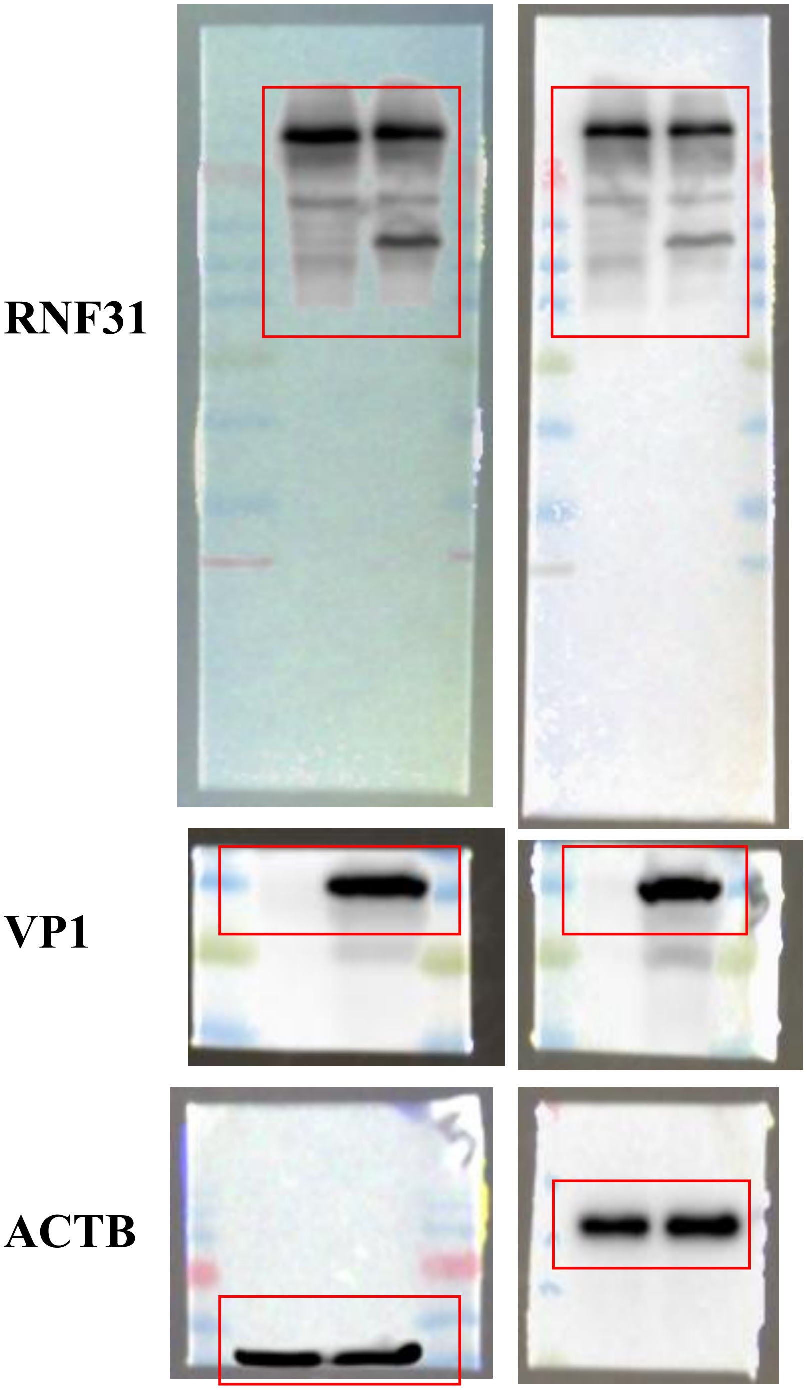

Figure 1H

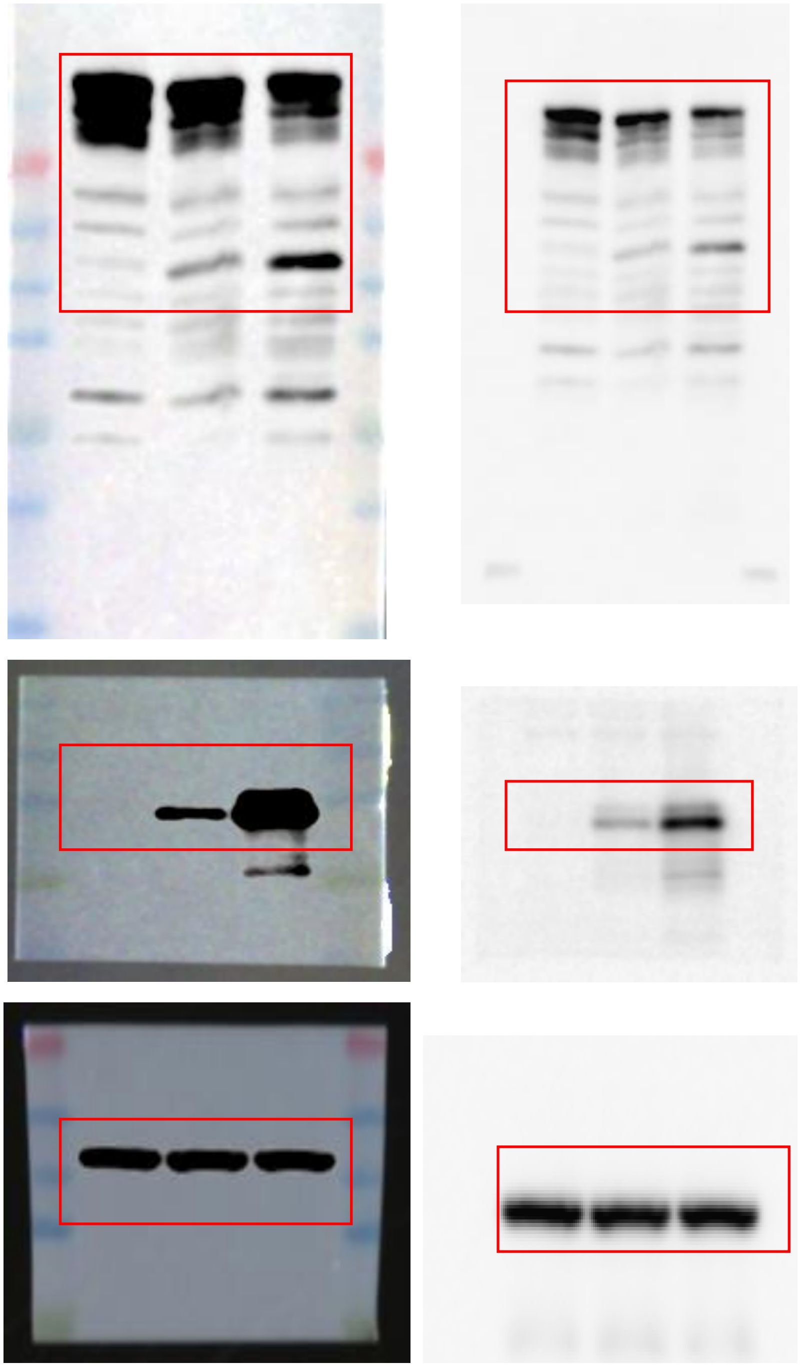

Flag

VP1

ACTB

Figure 2A

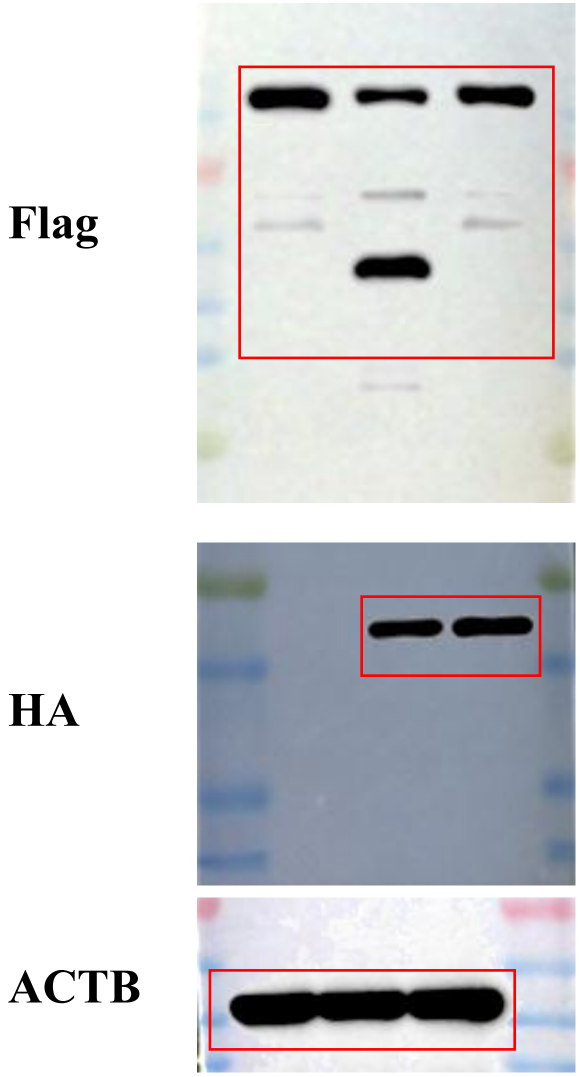

Figure 2B

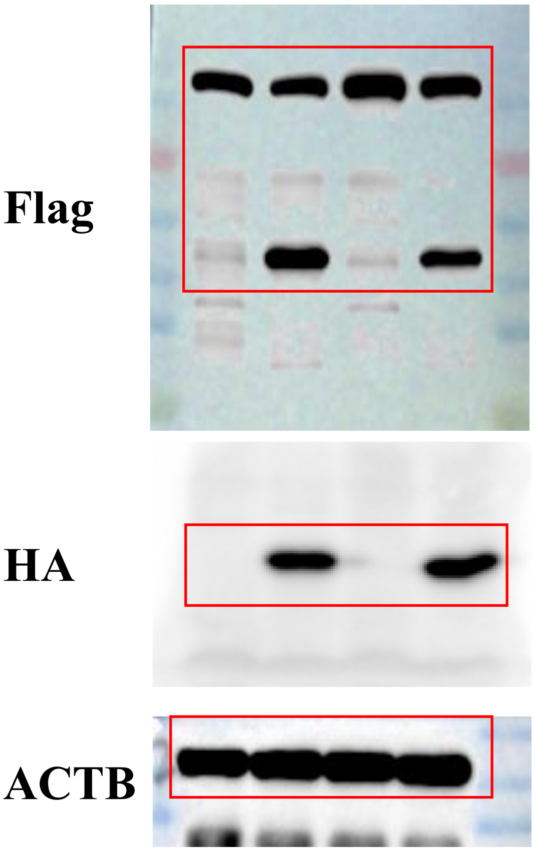

Figure 2C

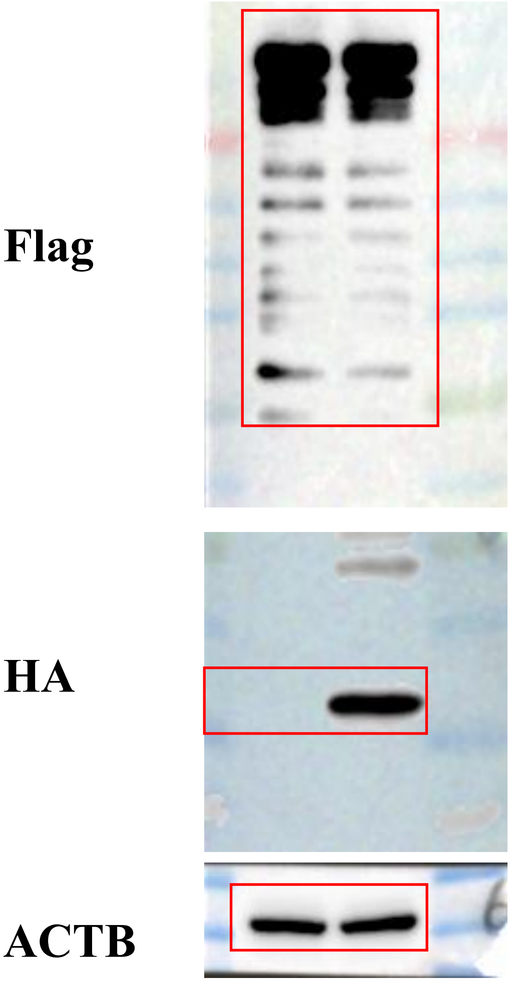

Figure 2D

Flag

HA

ProCASP3

Cleaved CASP3

ACTB

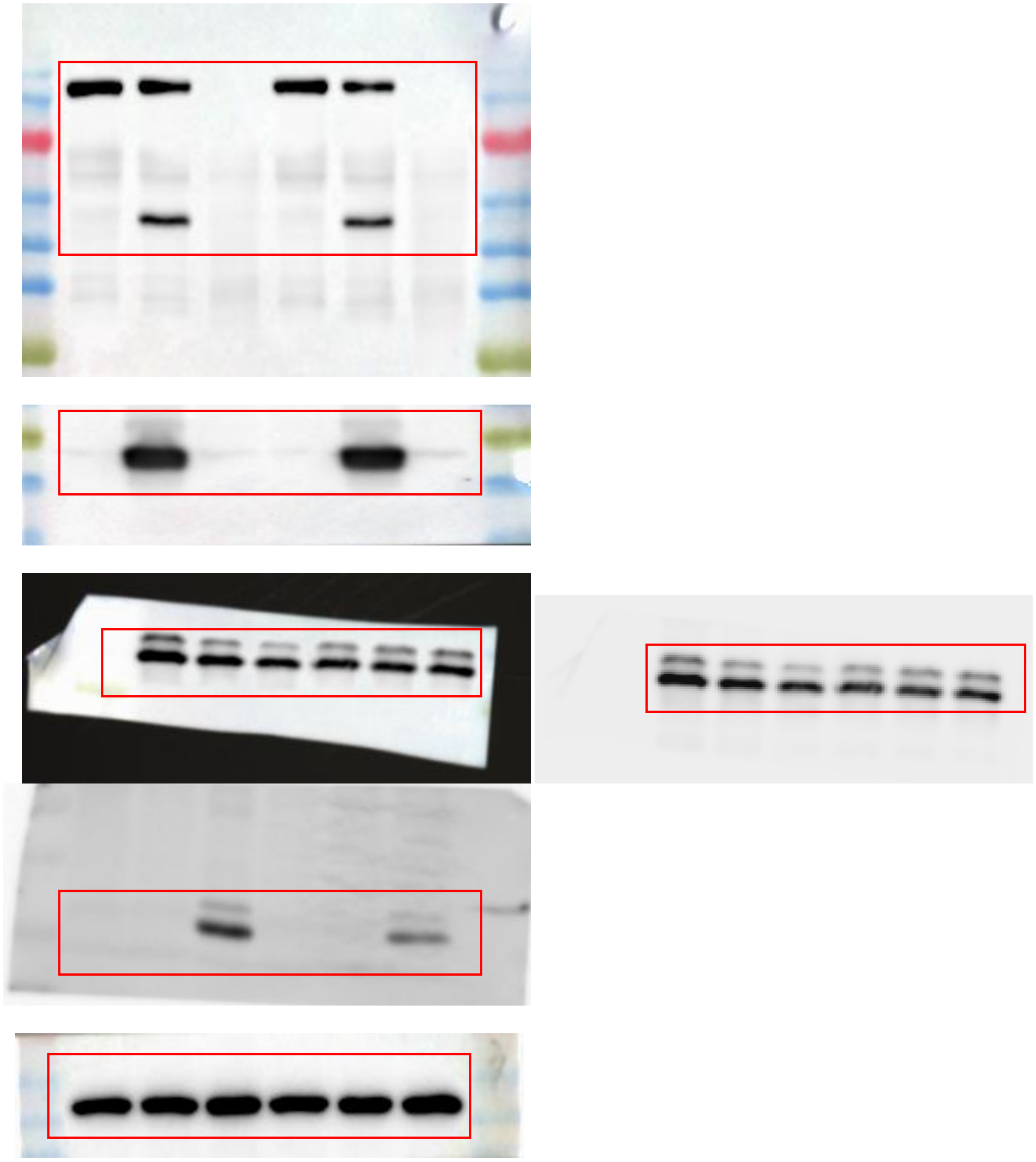

Figure 2G

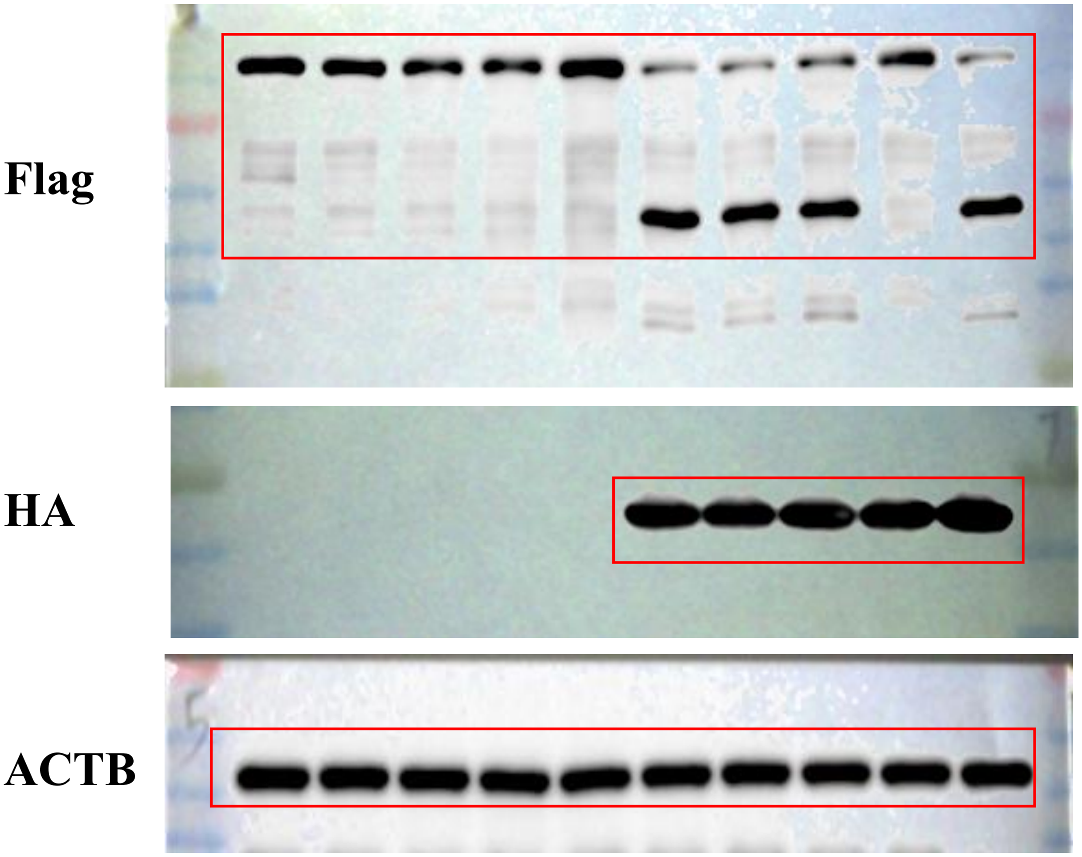

Figure 2H

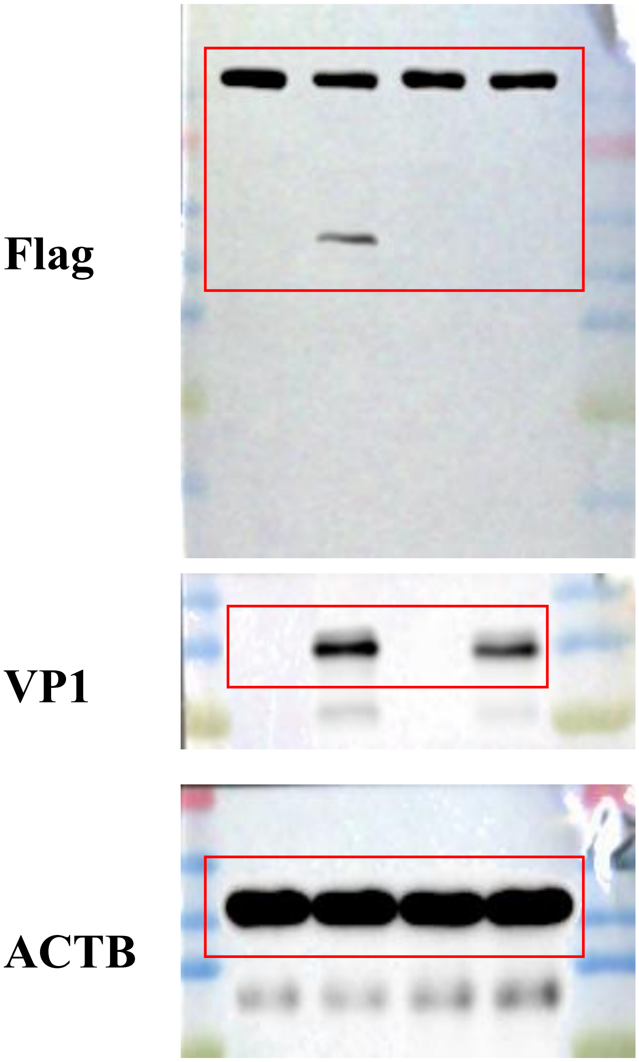

Figure 2J

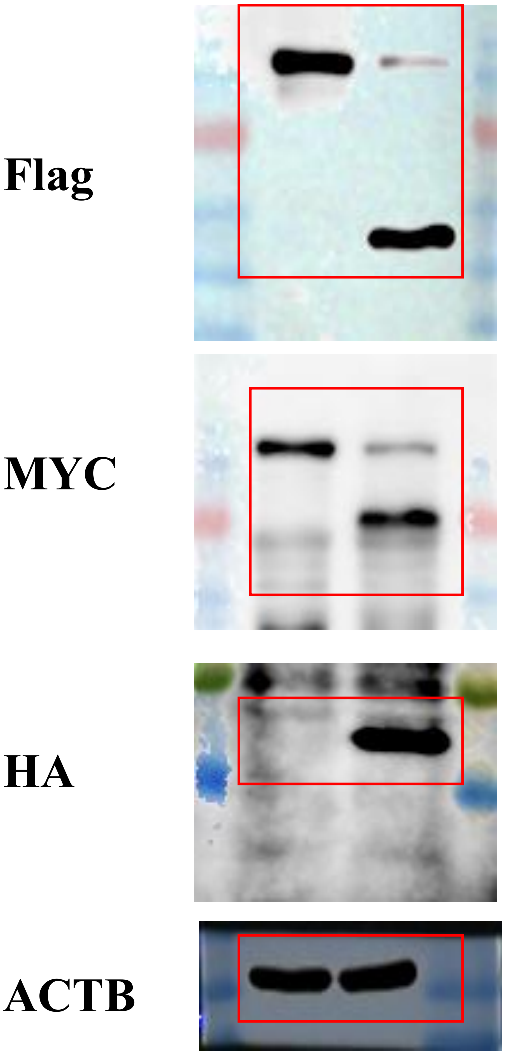

Figure 3A

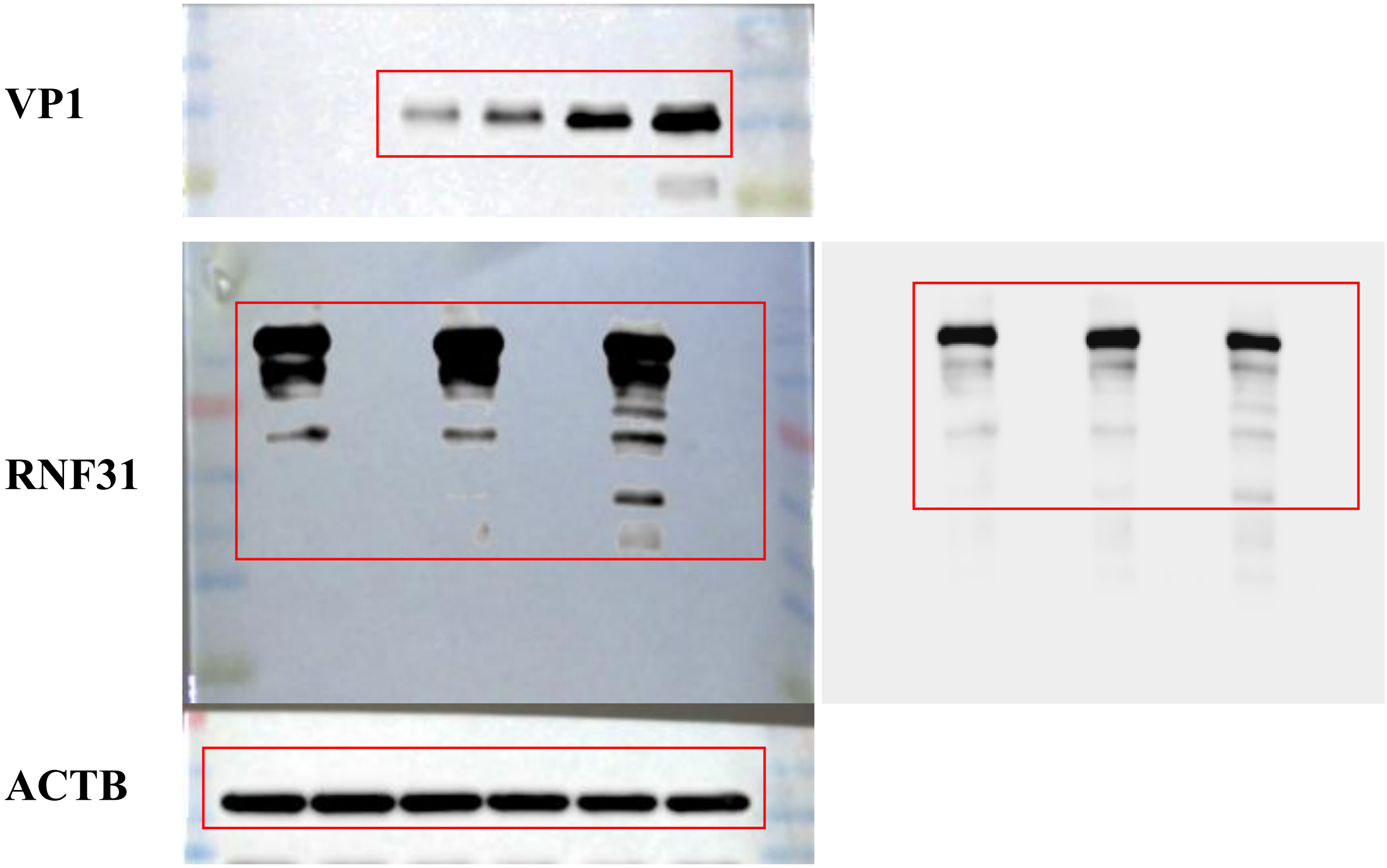

Figure 3F

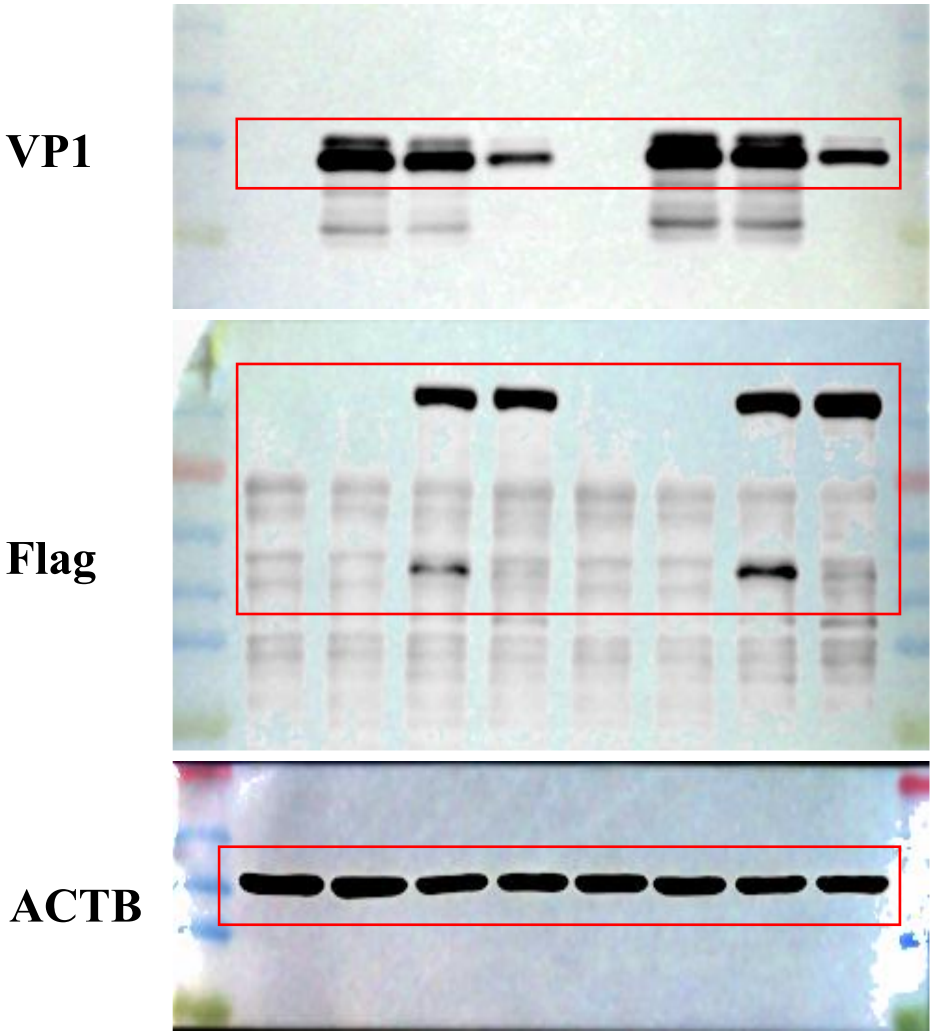

Figure 4A

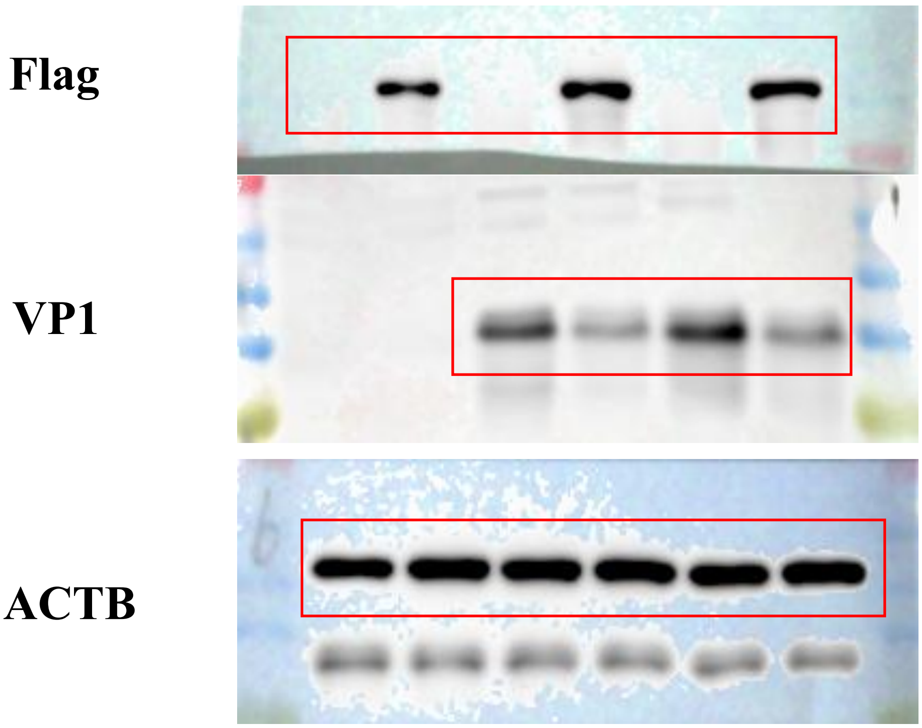

Figure 4B

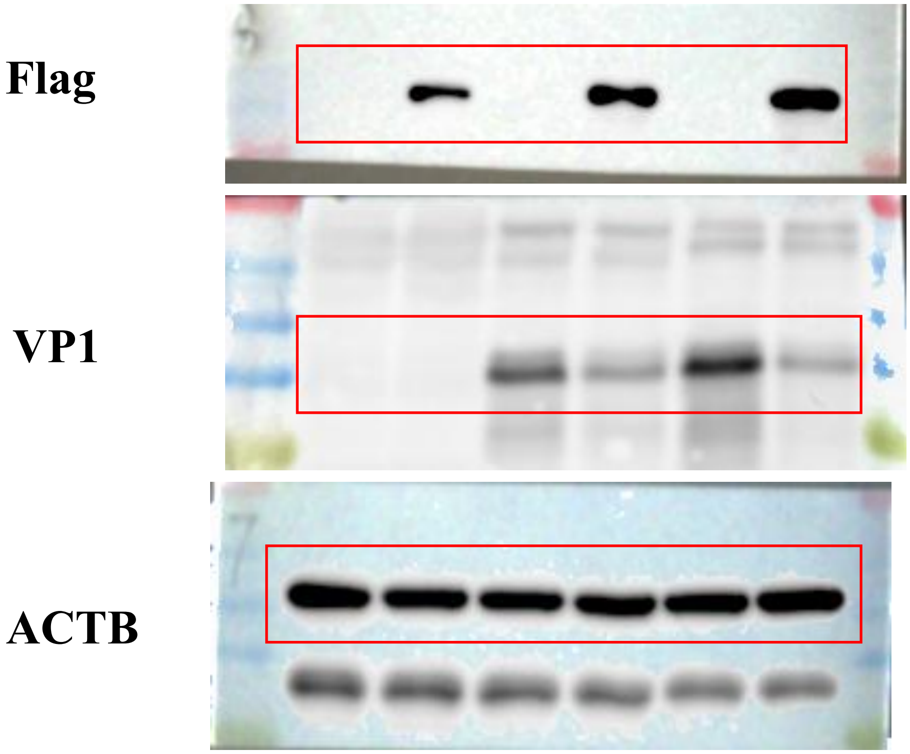

Figure 4C

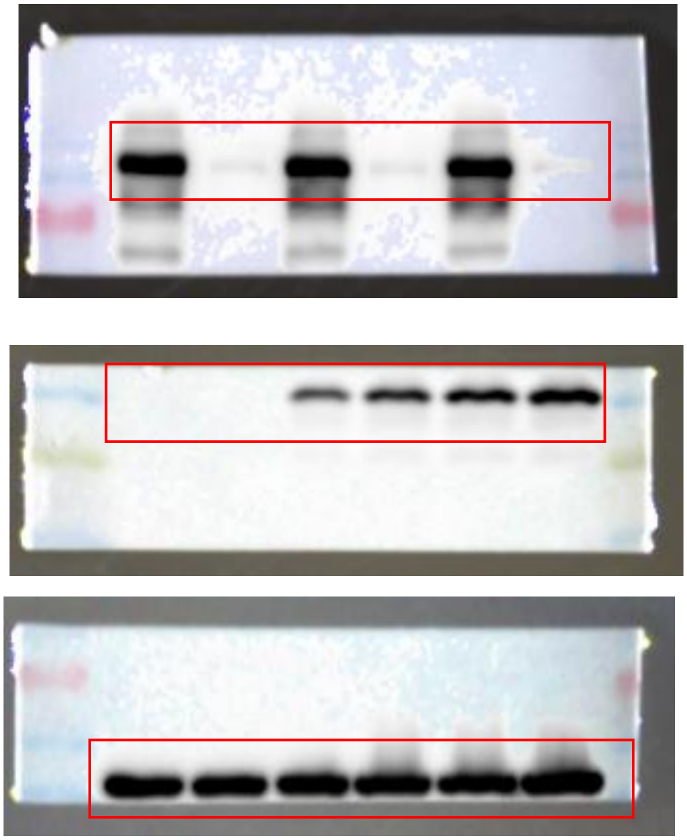

Figure 4D

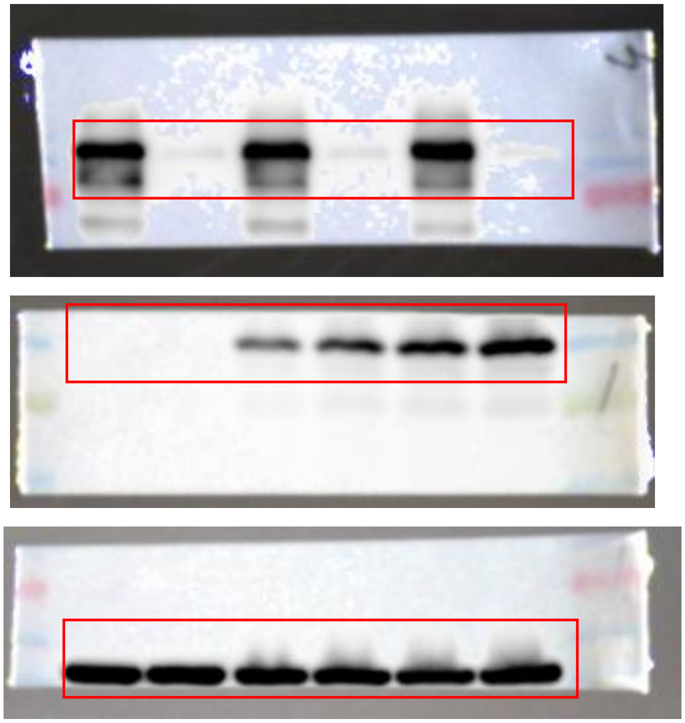

Figure 4E

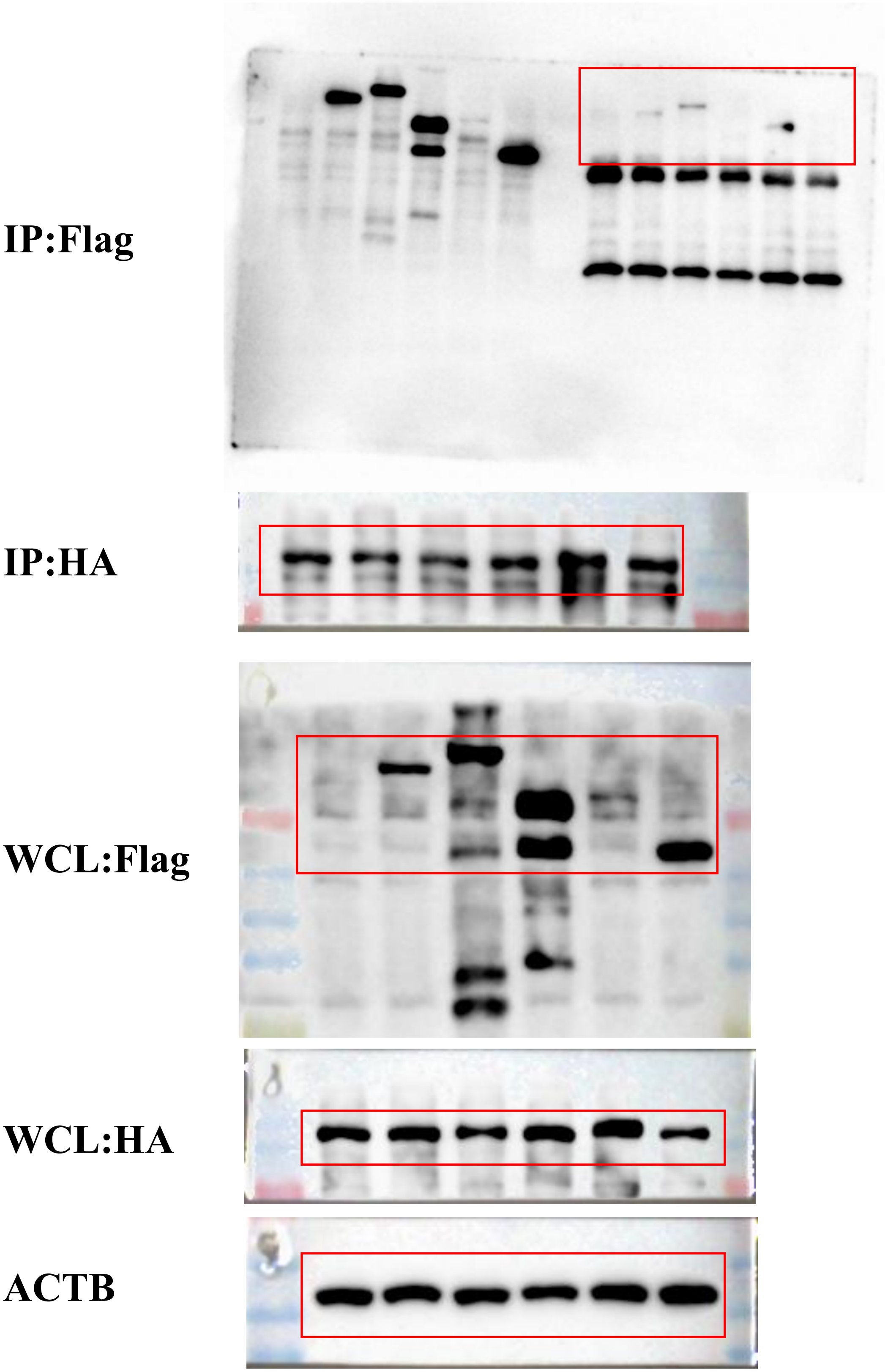

Figure 4F

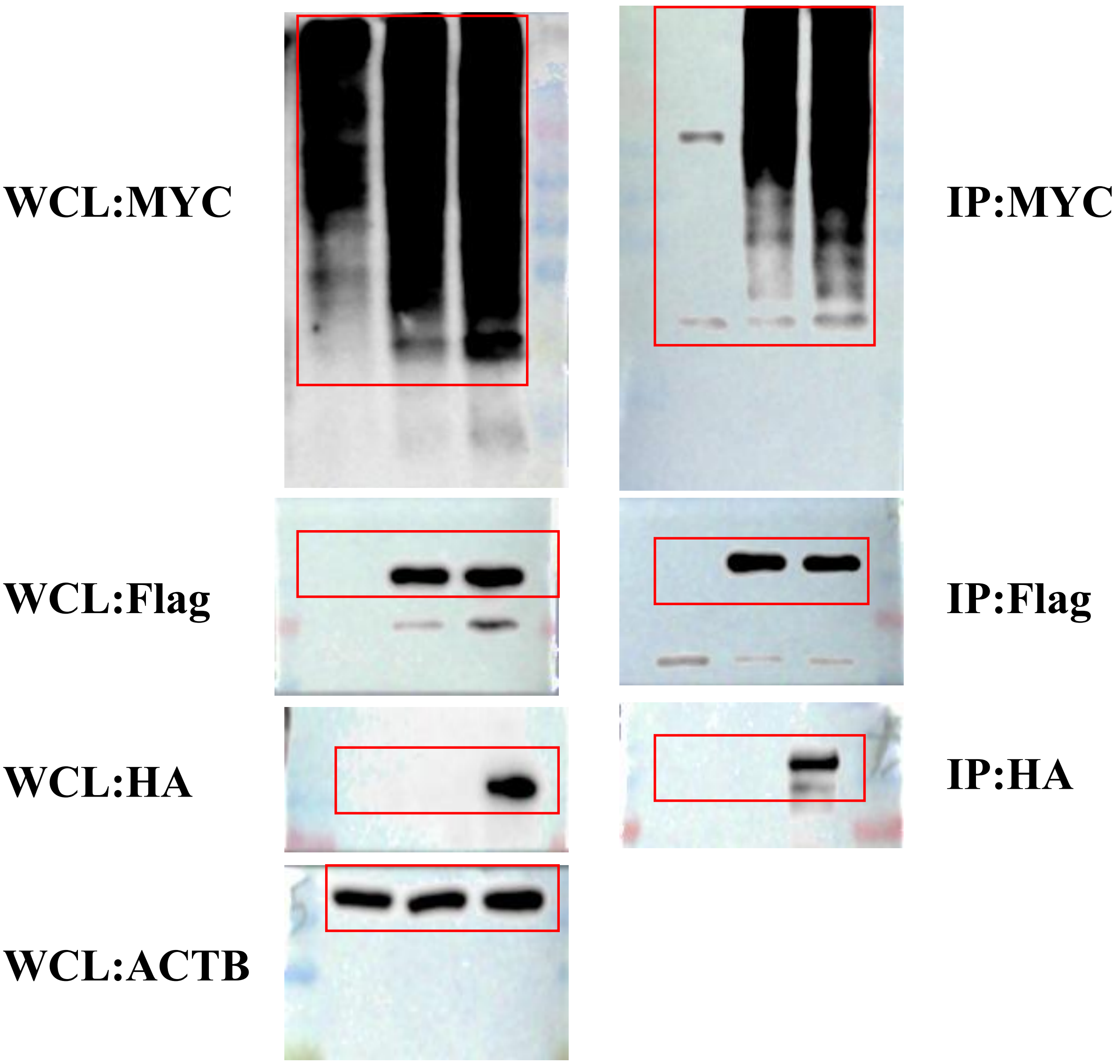

Figure 4G

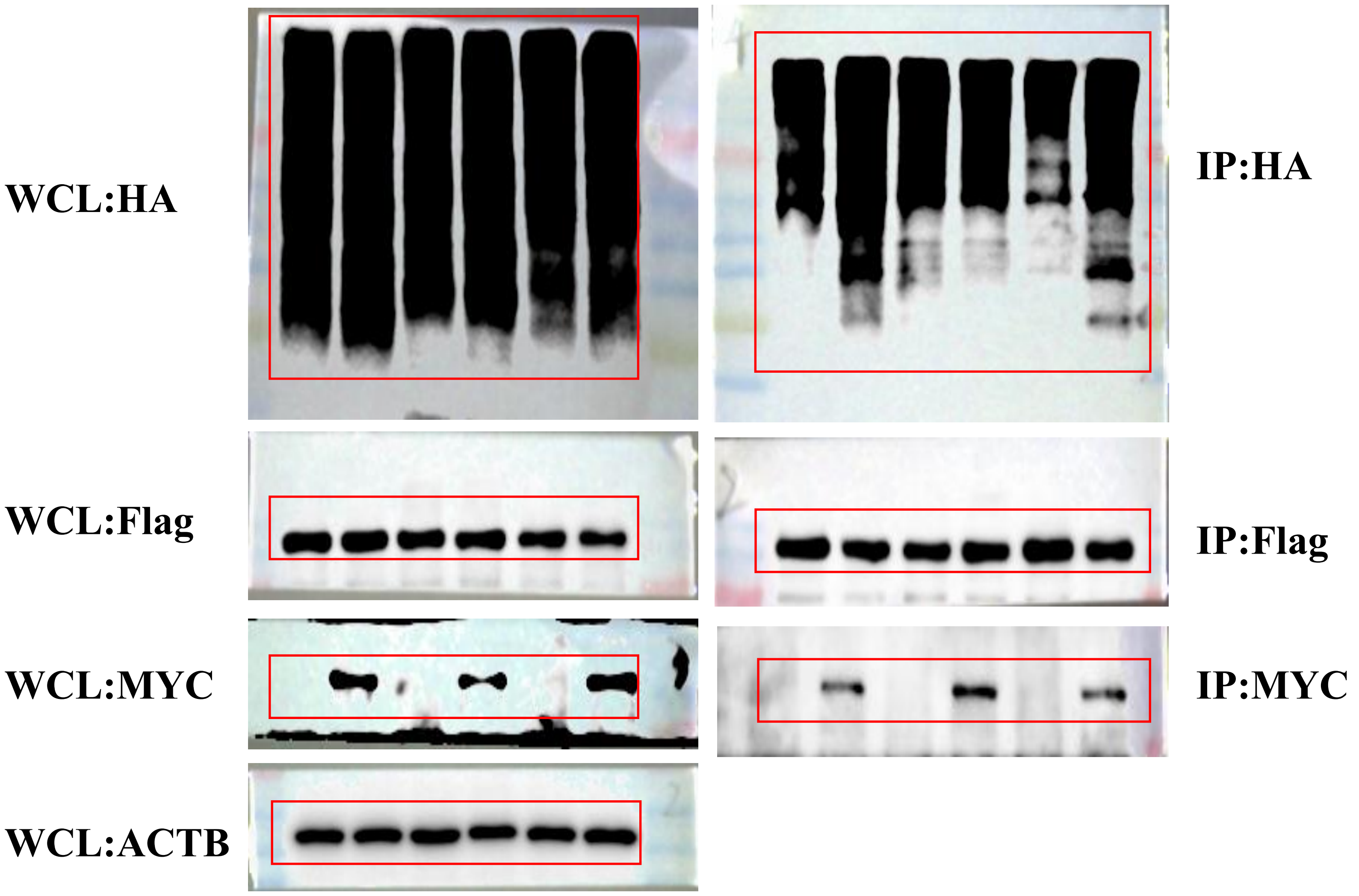

Figure 5A

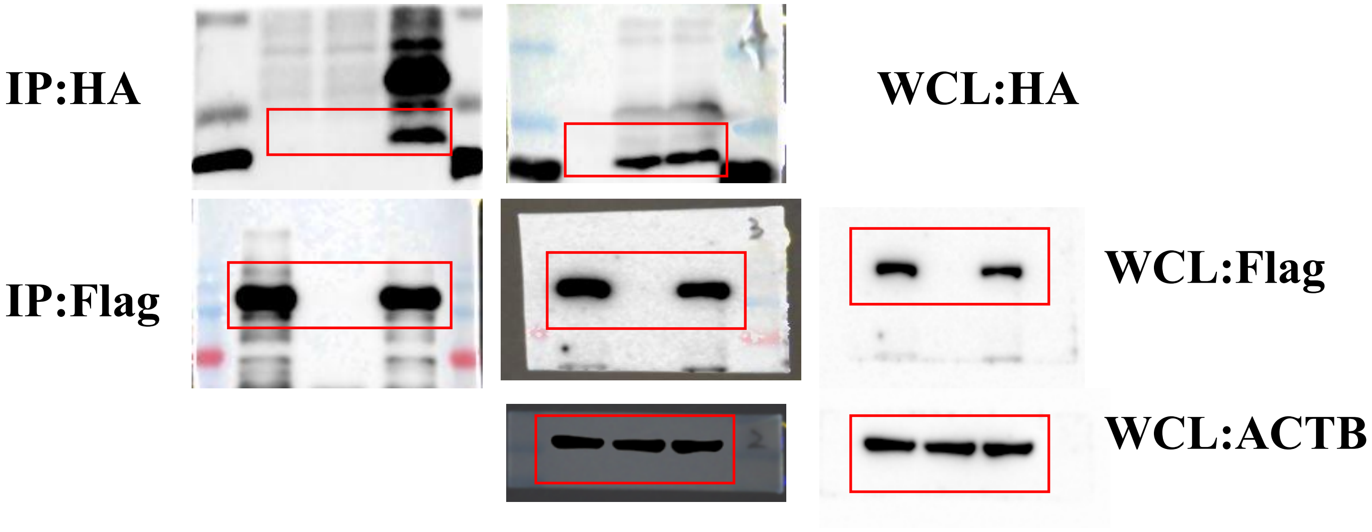

Figure 5B

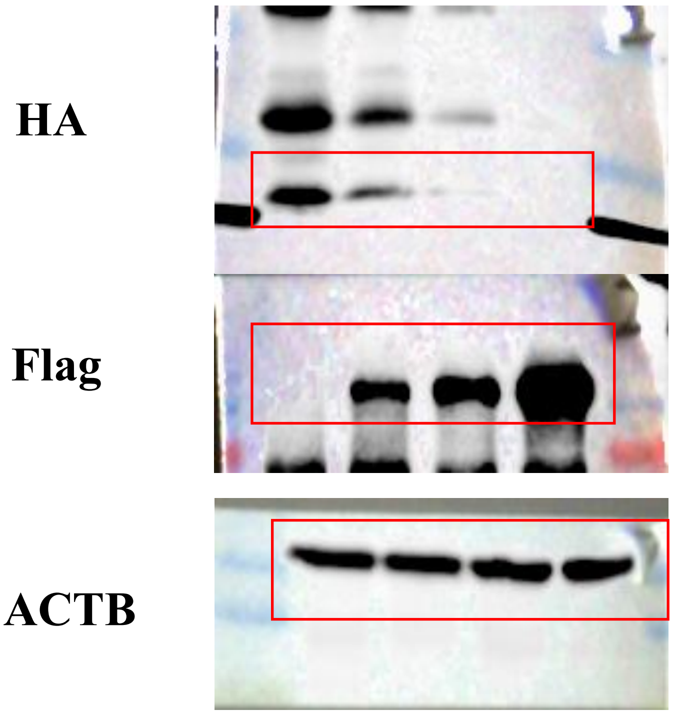

Figure 5C

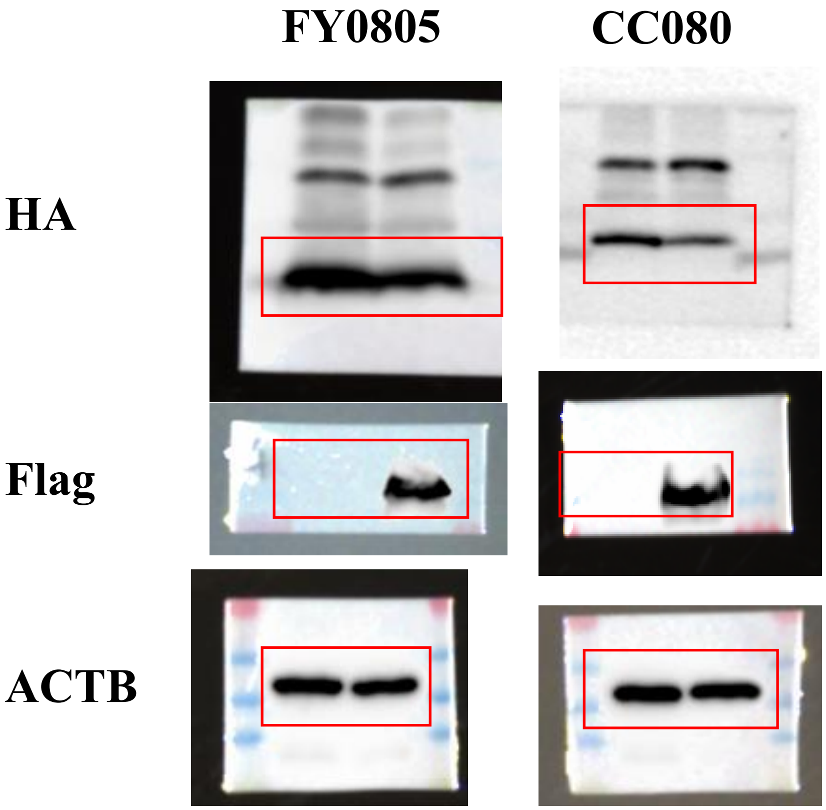

Figure 5D

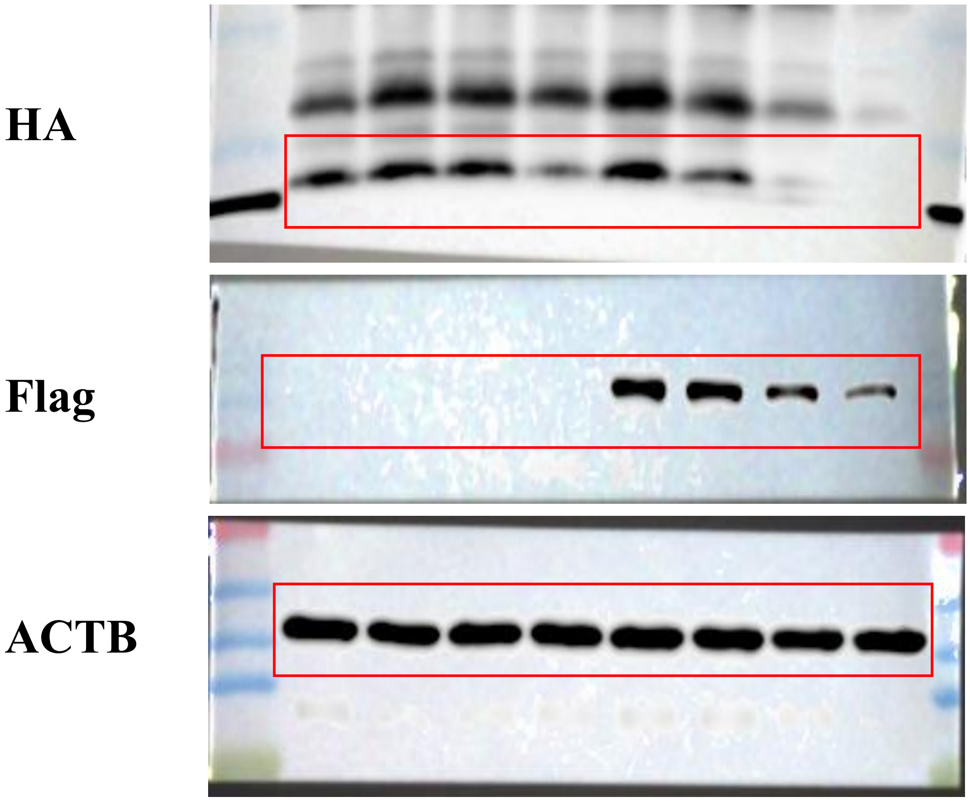

Figure 5I

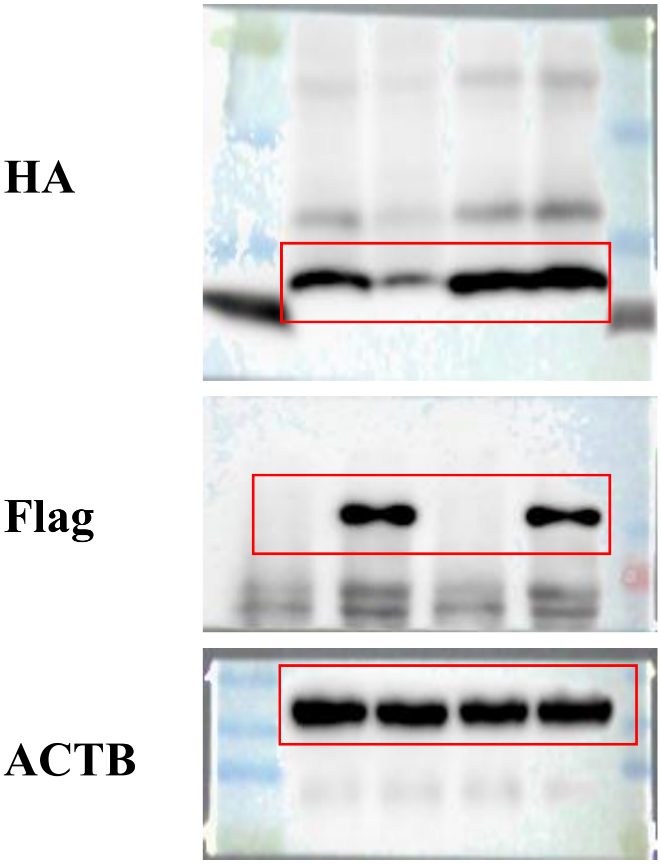

Figure 5J

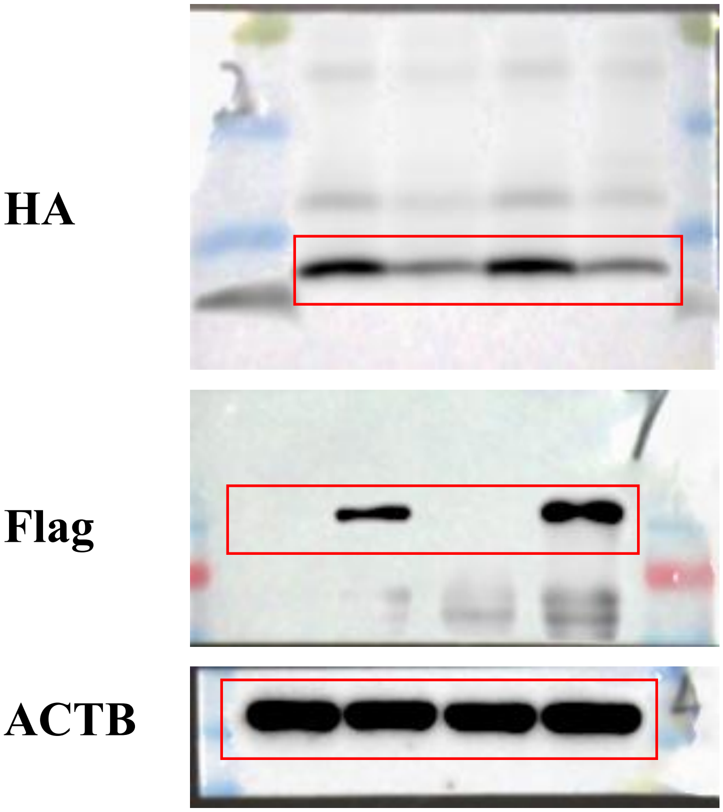

Figure 5K

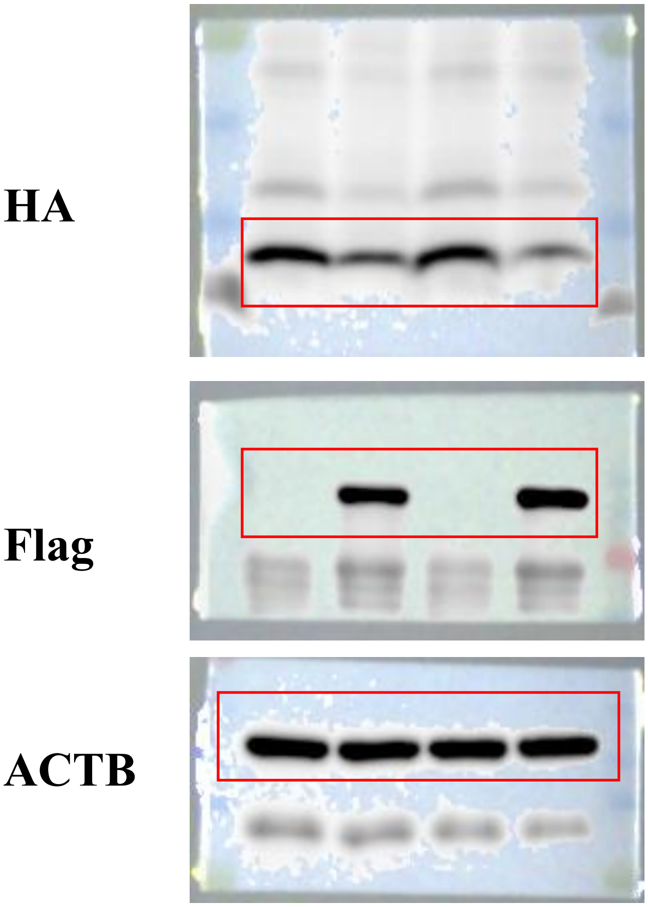

Figure 6A

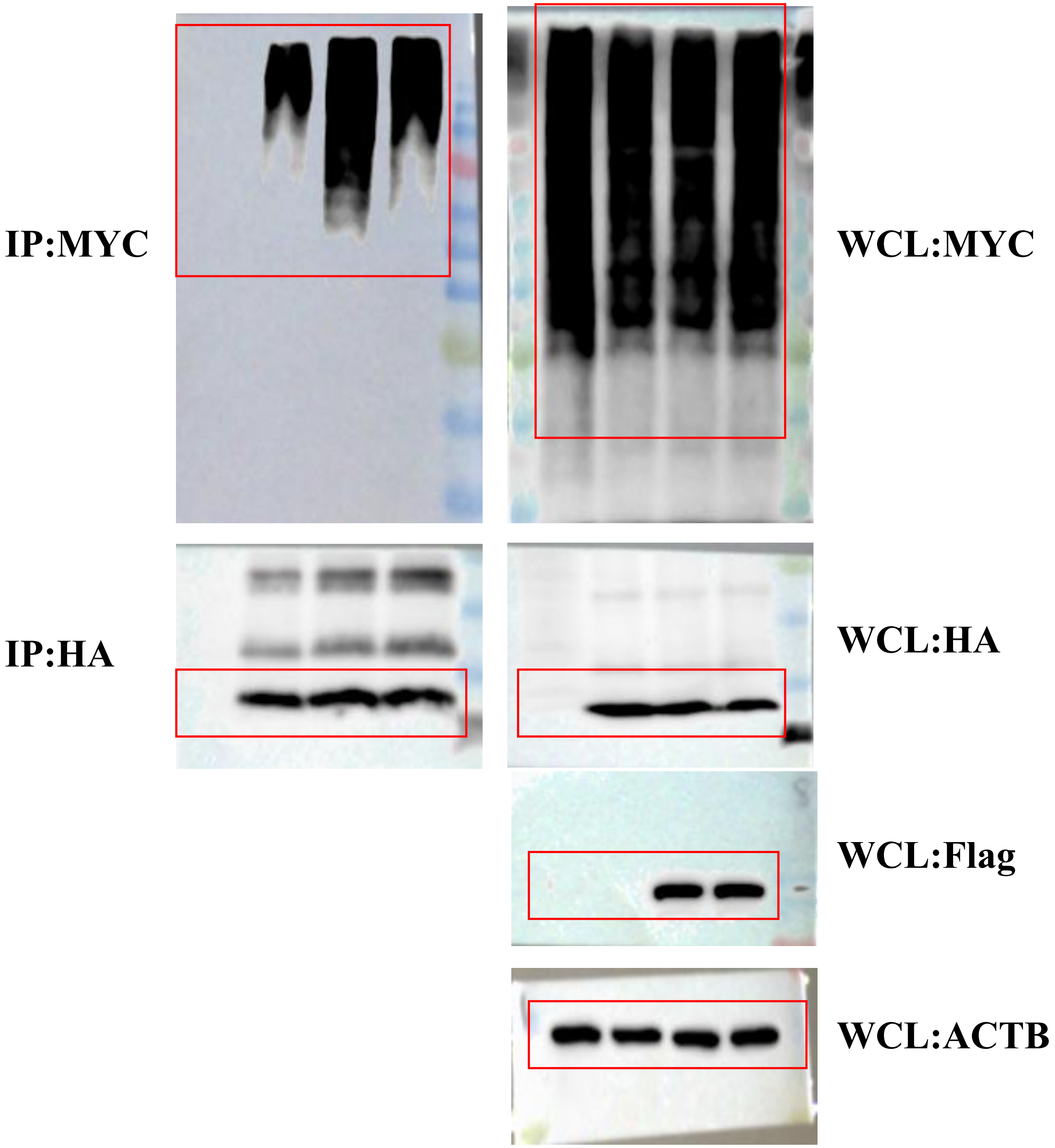

Figure 6B

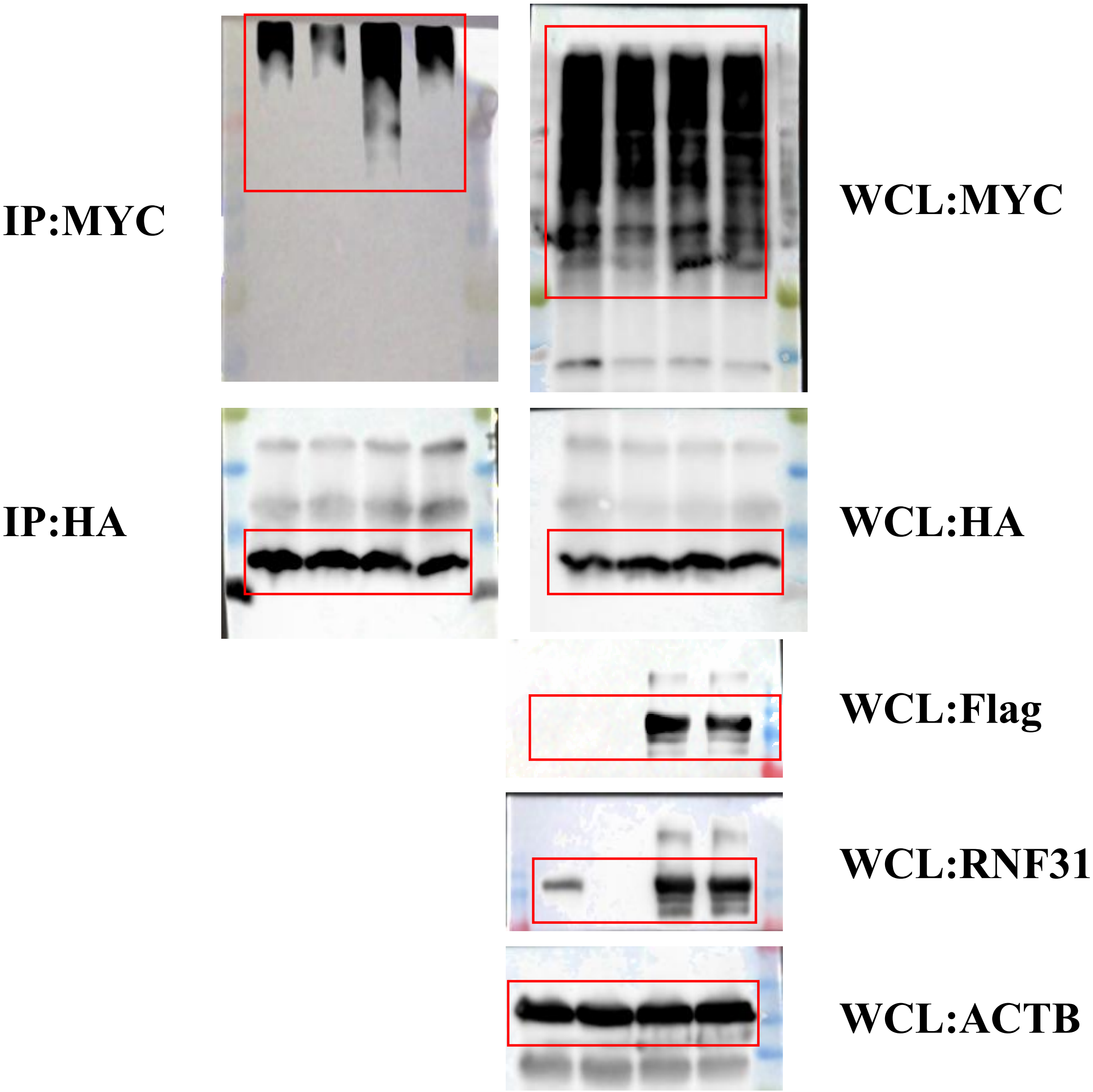

Figure 6C

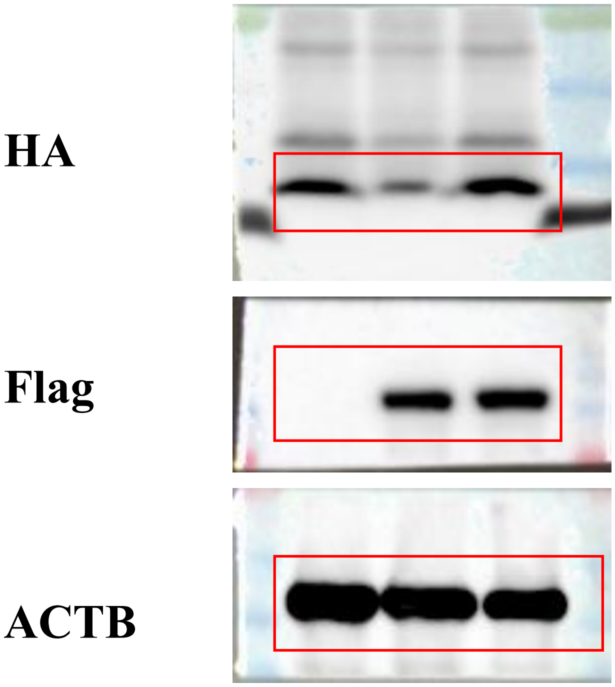

Figure 6D

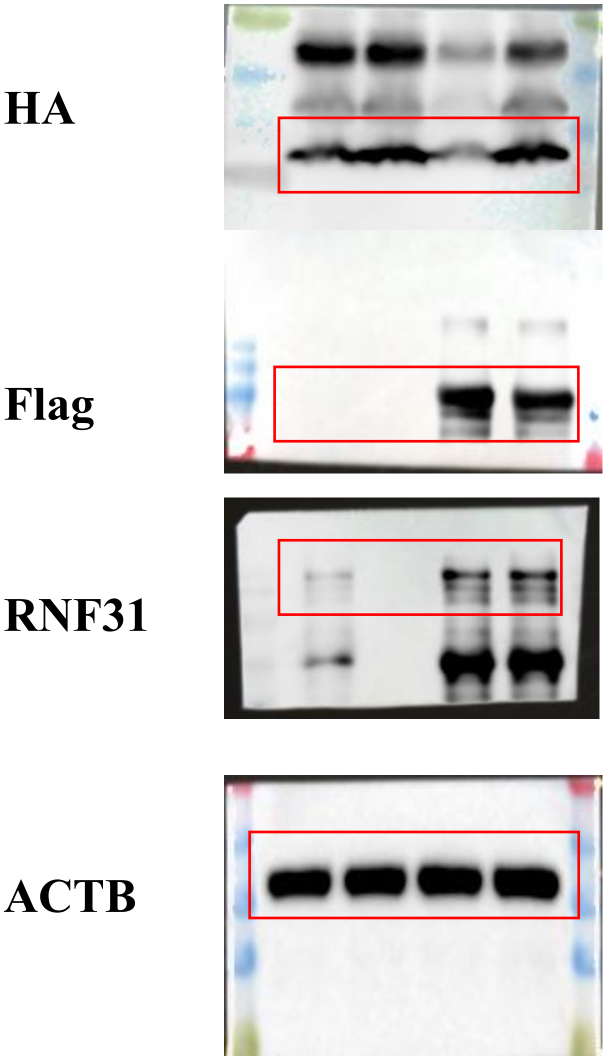

Figure 6E

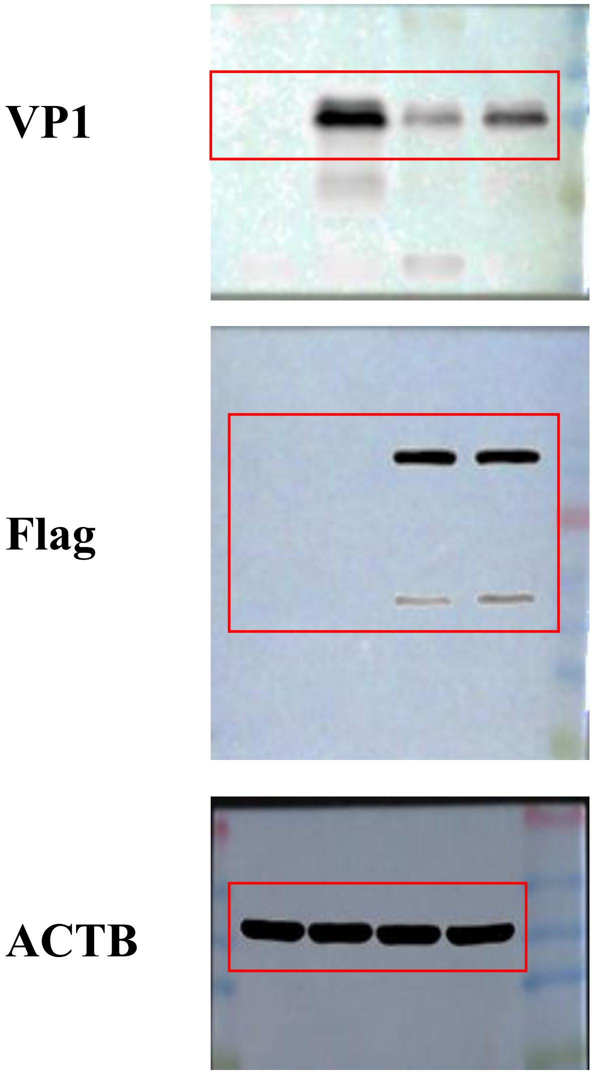

Figure 6H

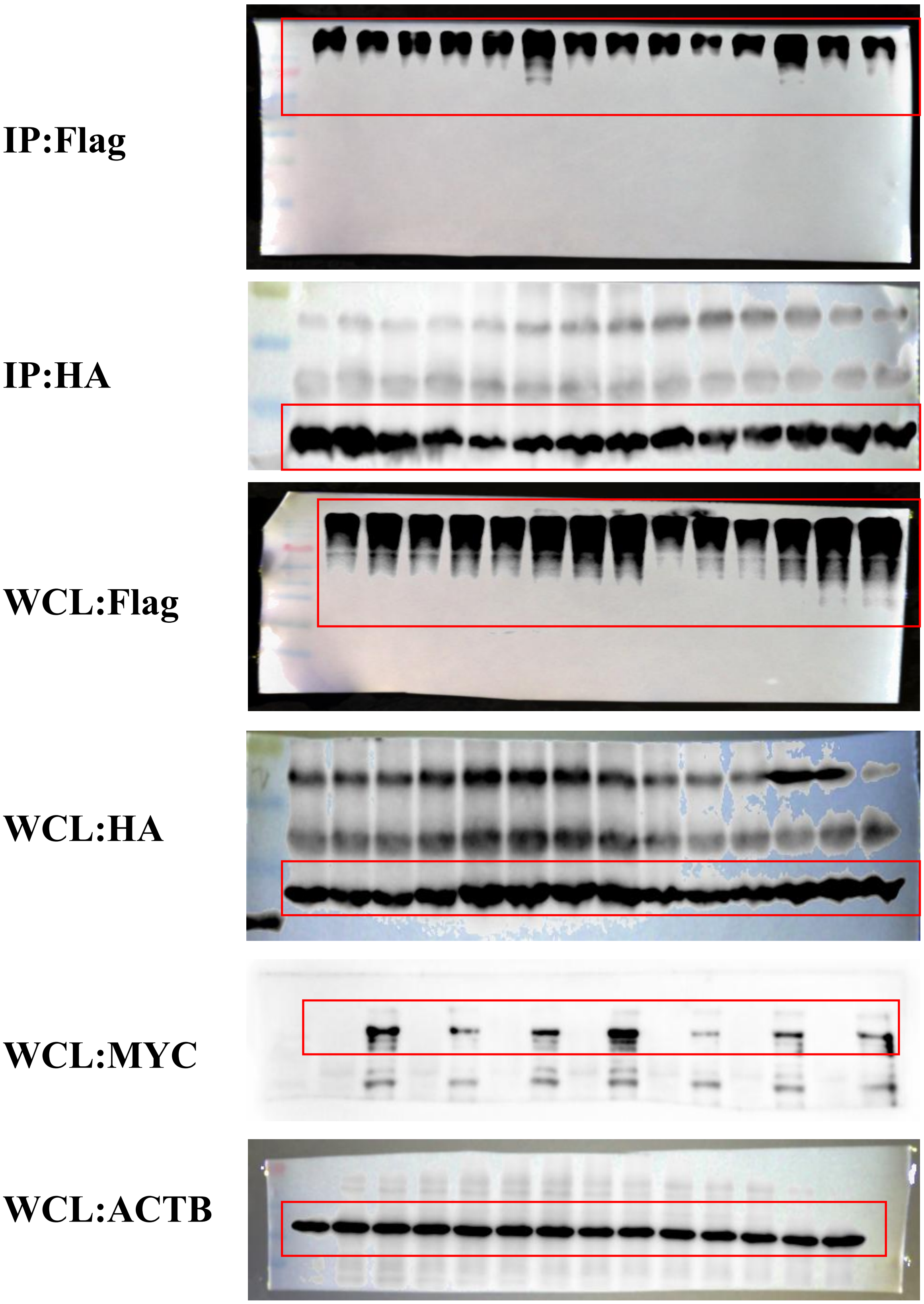

Figure 6J

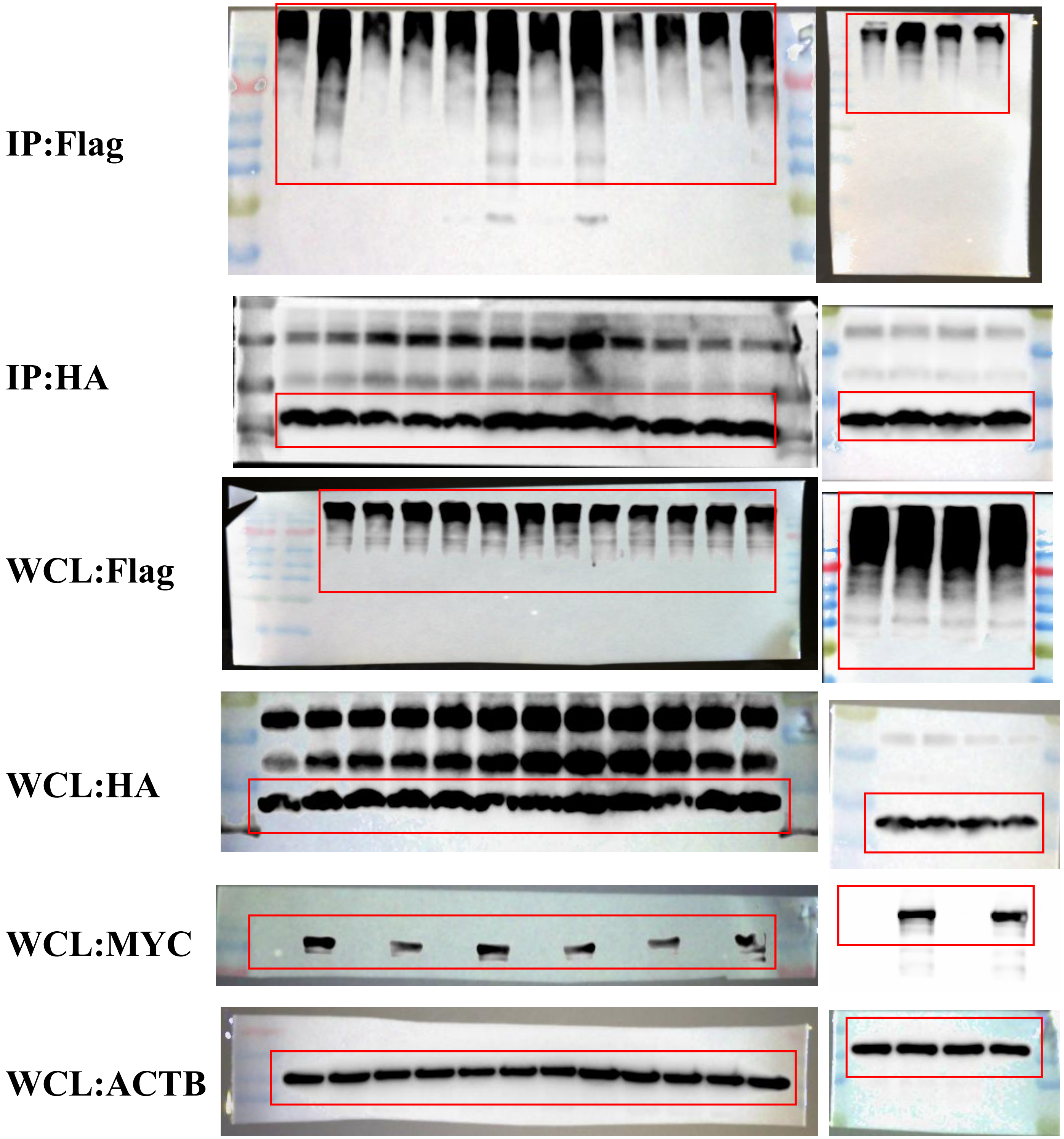

Figure 6K

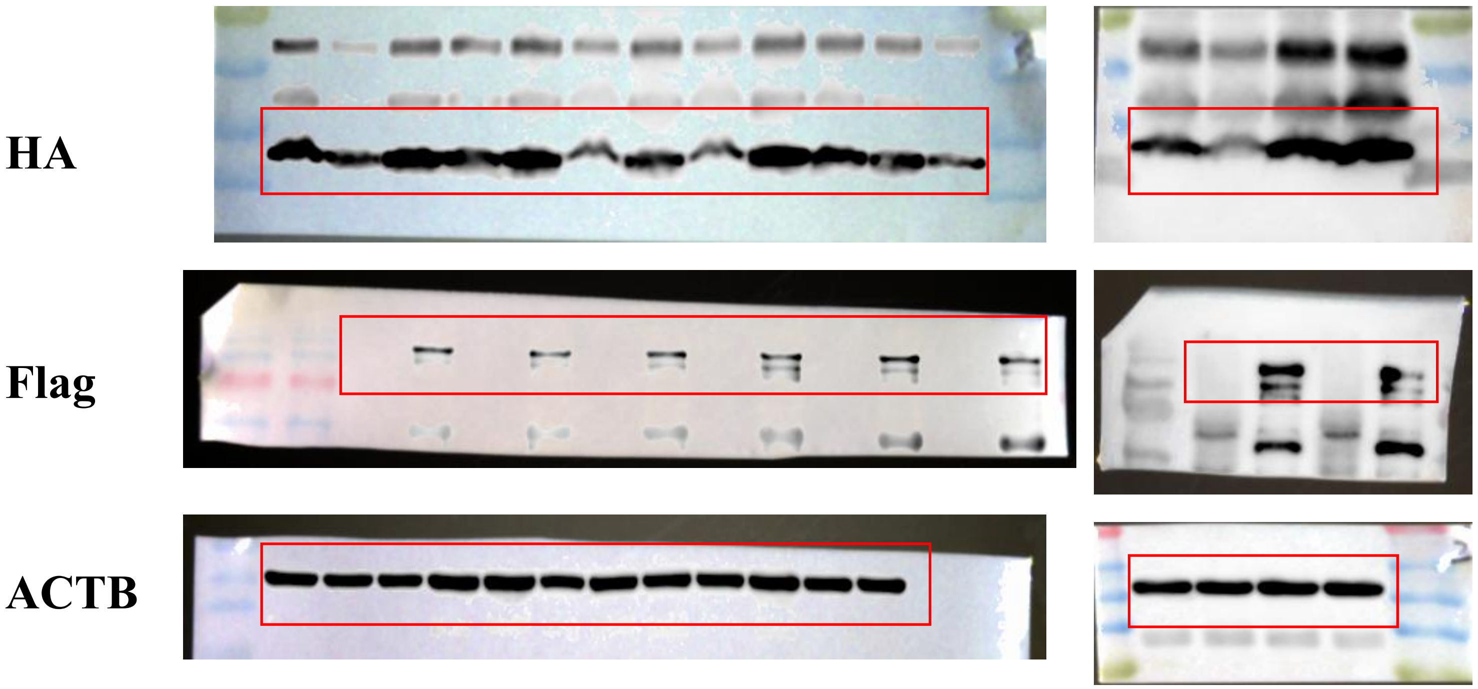

Figure 7A

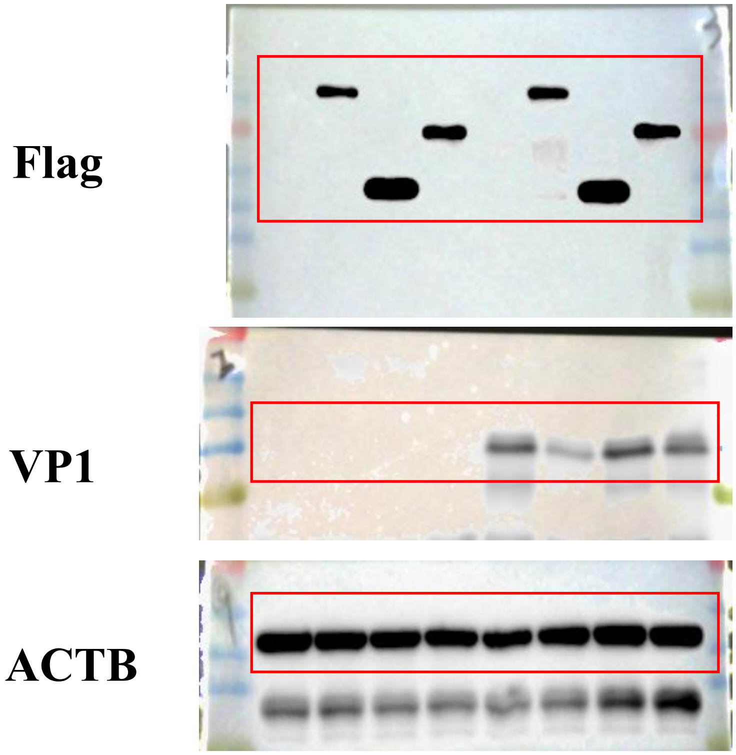

Figure 7B

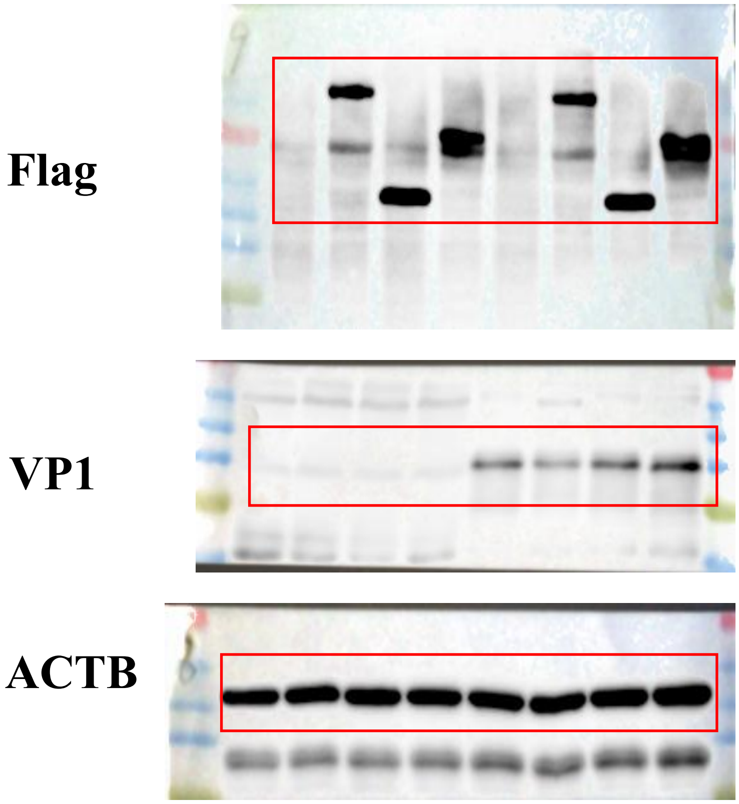

Figure 7C

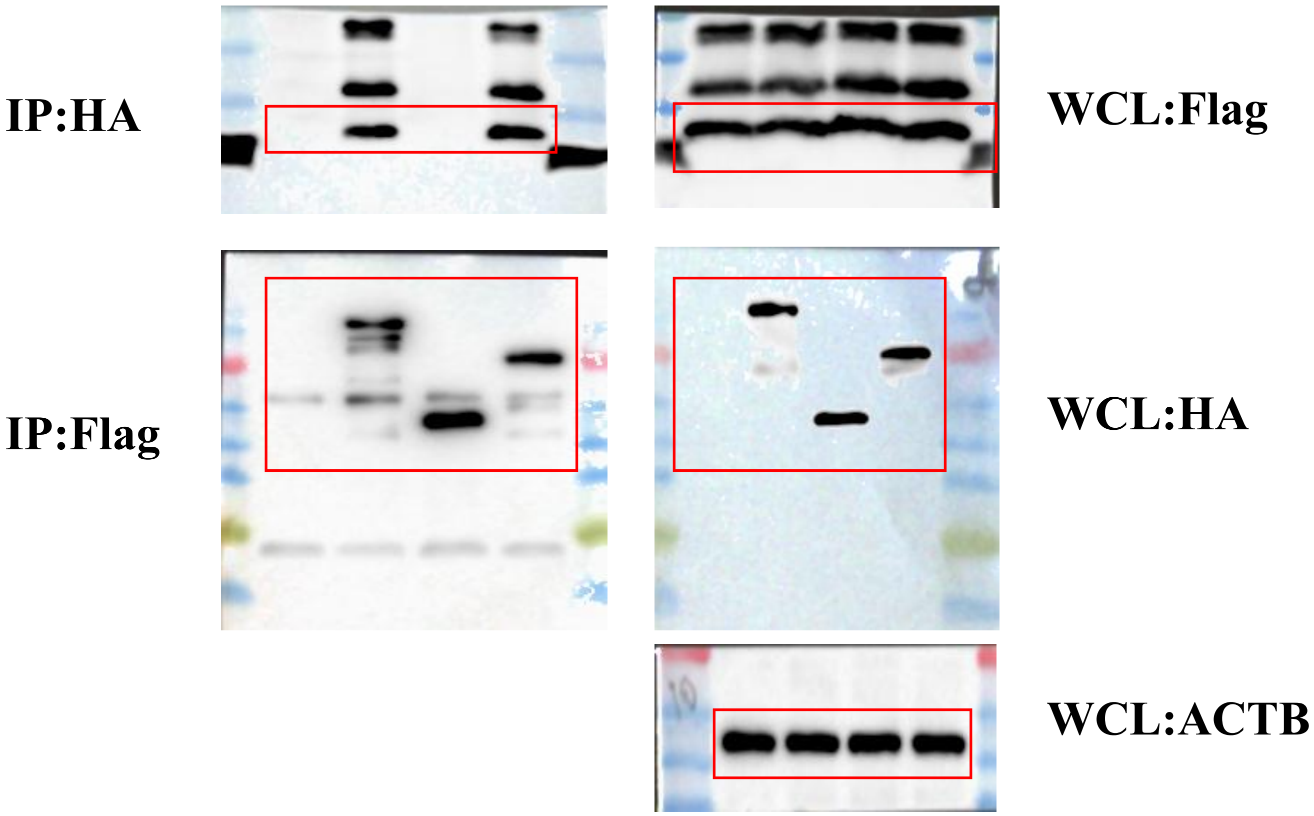

Figure 7D

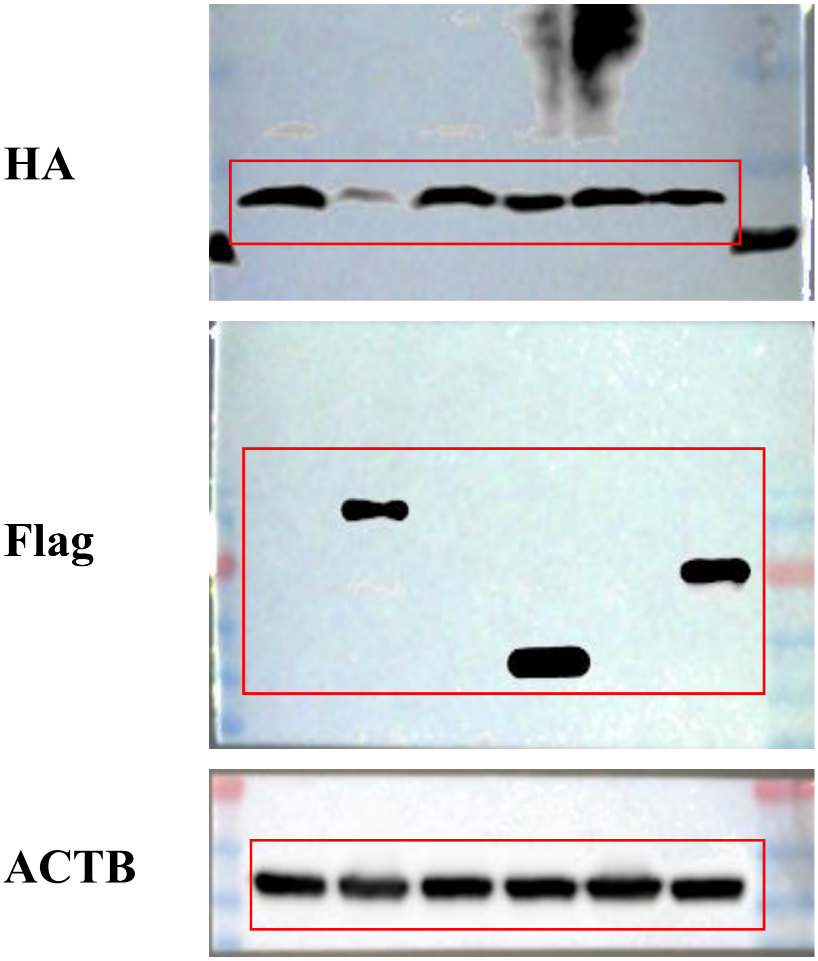

Figure 7E

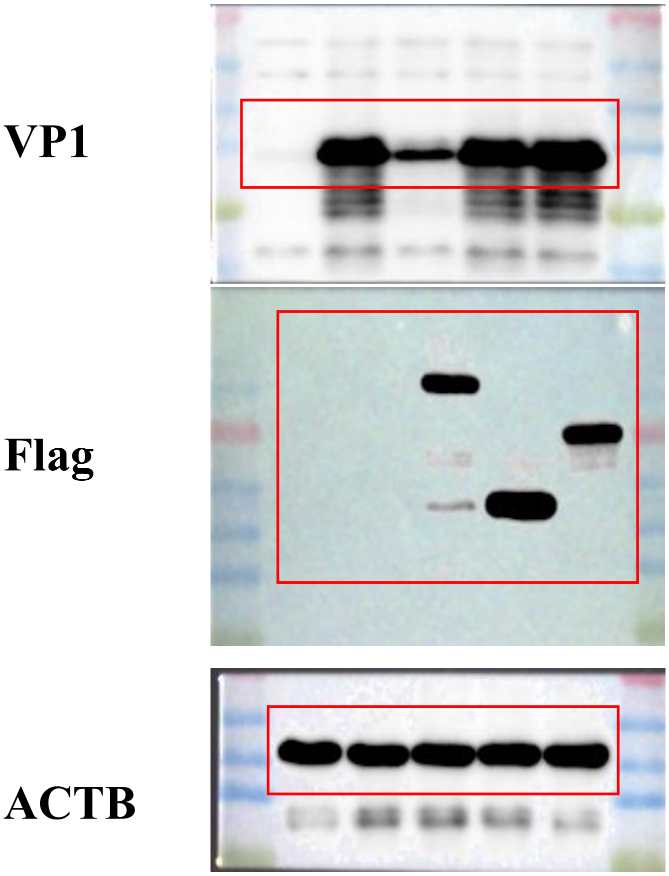

Figure 7H

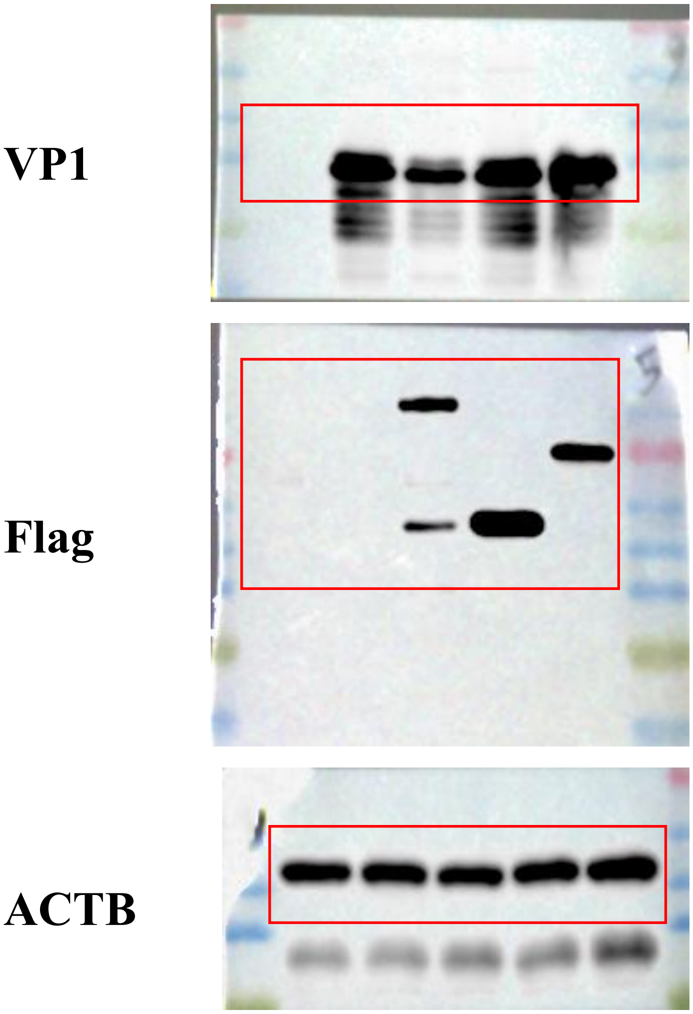

Figure 8A

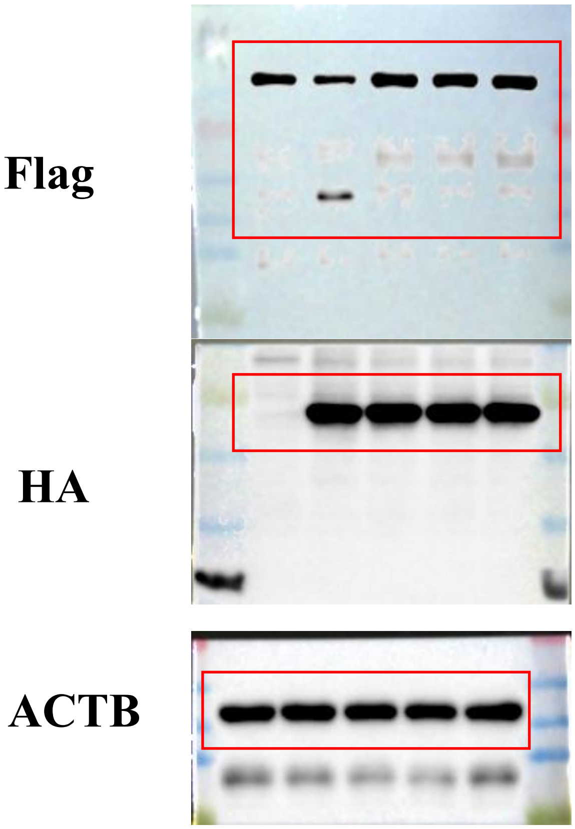

Figure 8B

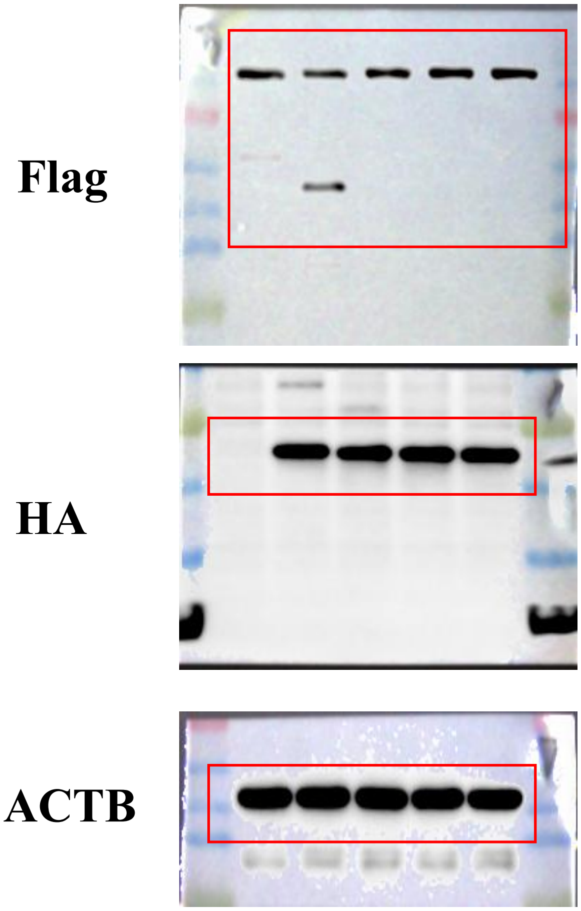

Figure 8C

Flag

HA

ACTB

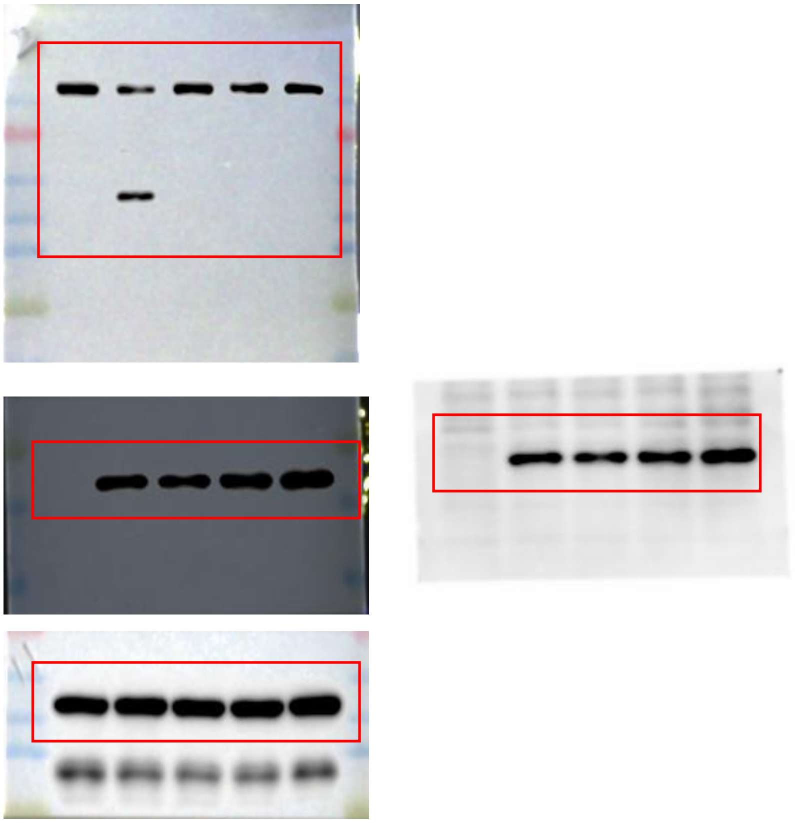

Figure 8D

Flag

HA

ACTB

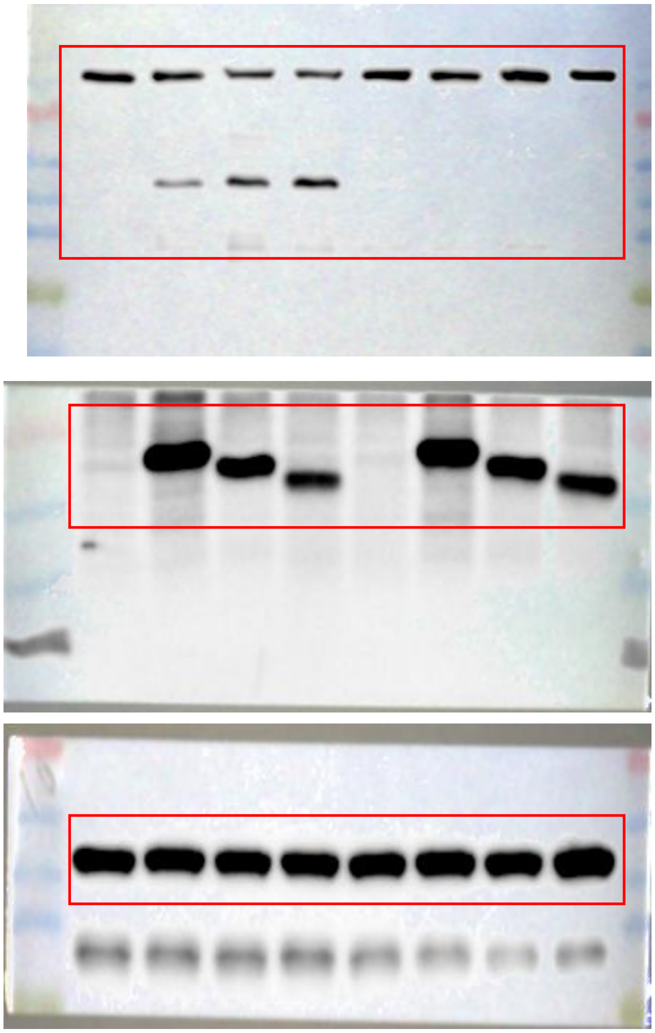

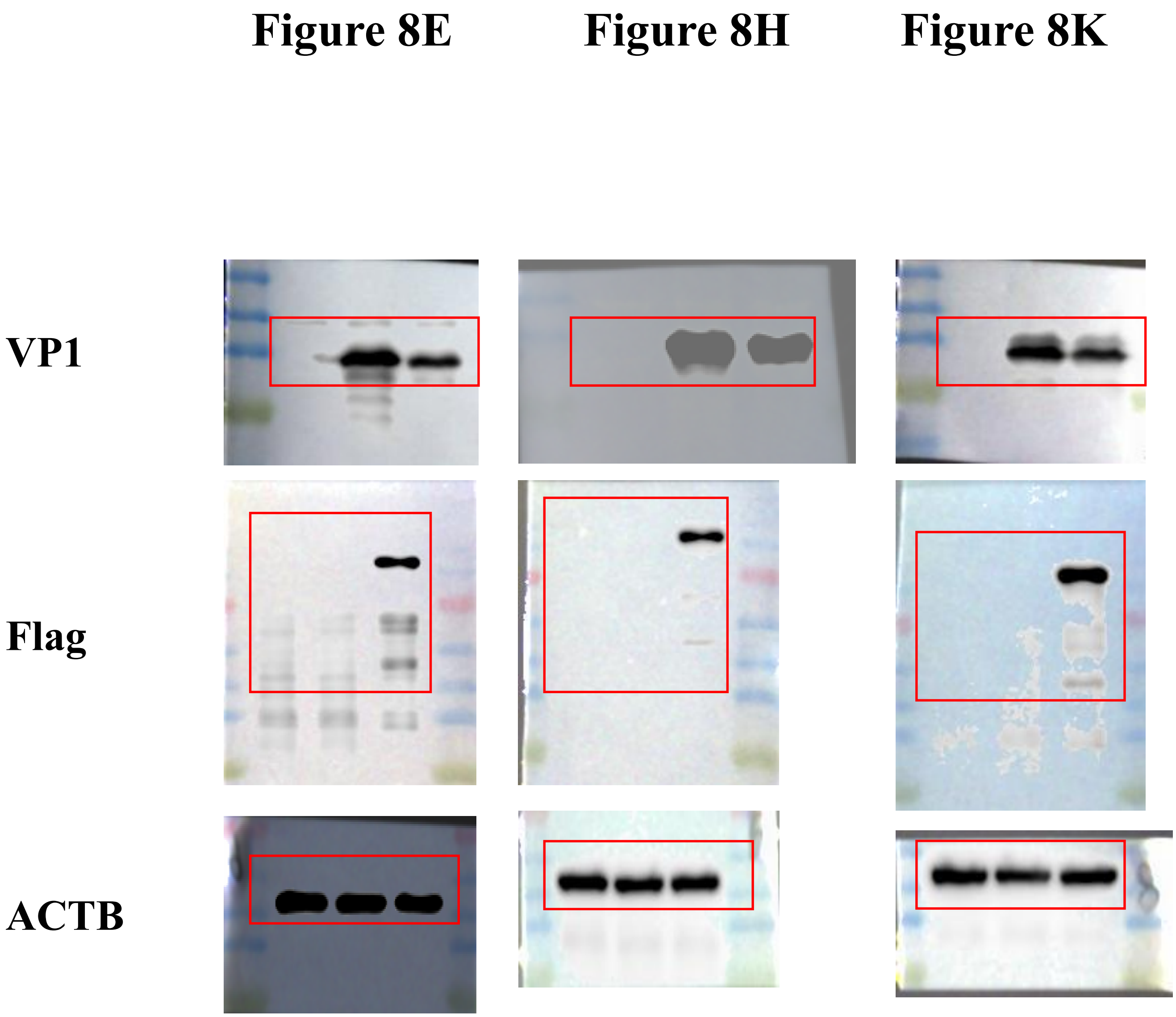
Figure 8E
Figure 8H
Figure 8K
VP1
Flag
ACTB

Figure S1B

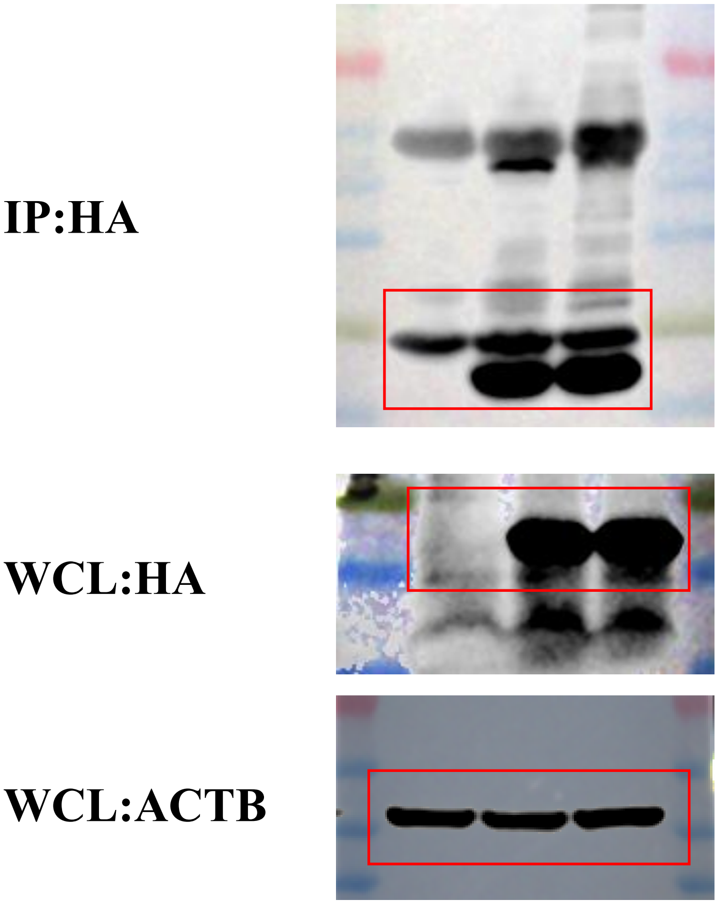

Figure S2C

RNF31

ACTB

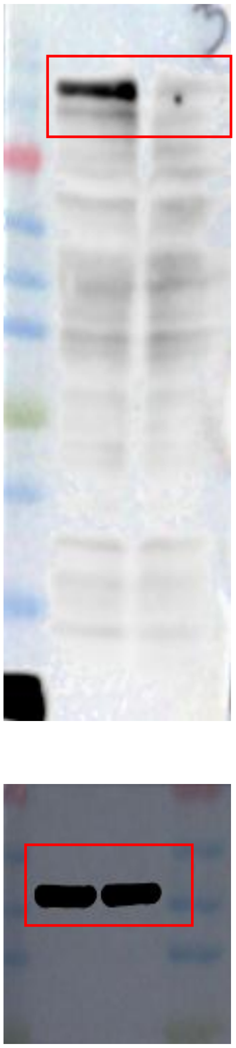

Figure S2F

RNF31

ACTB

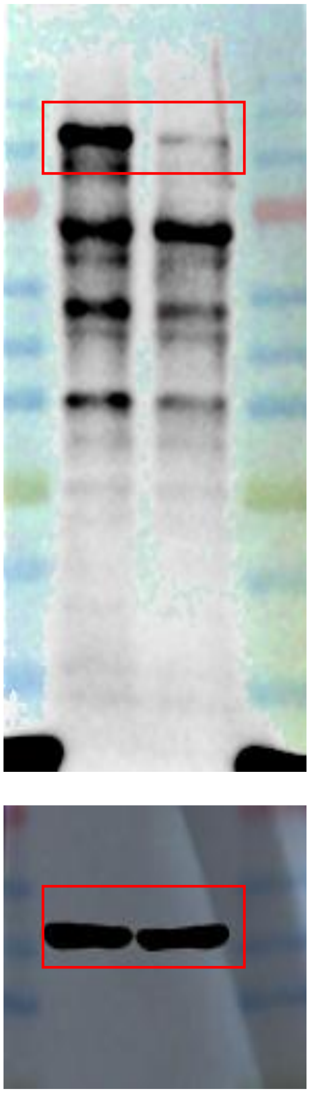

Figure S2H

VP1

RNF31

ACTB

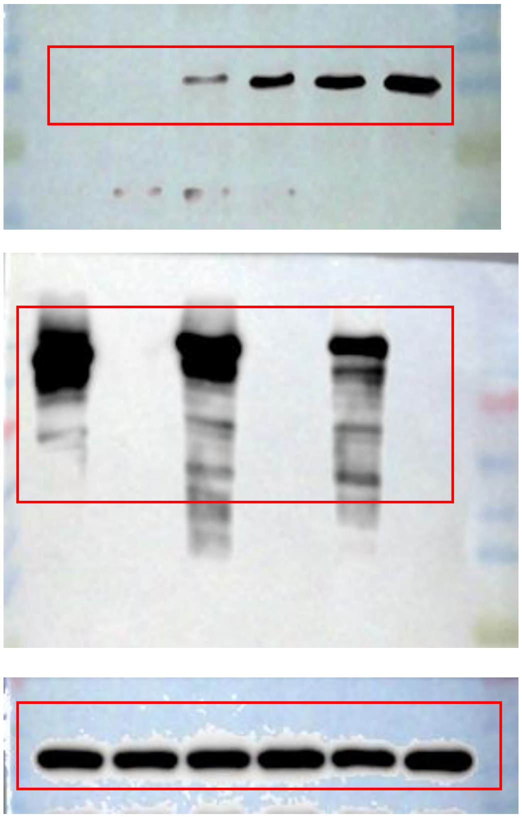

Figure S2K

VP1

Flag

ACTB

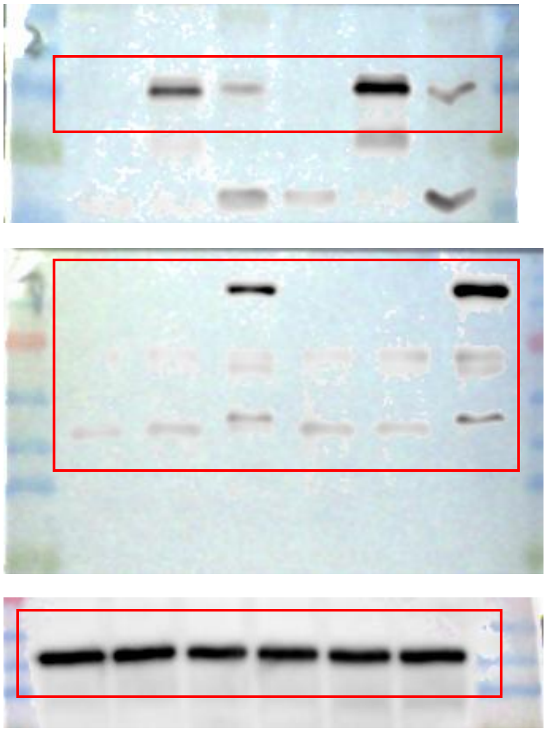

Figure S4A

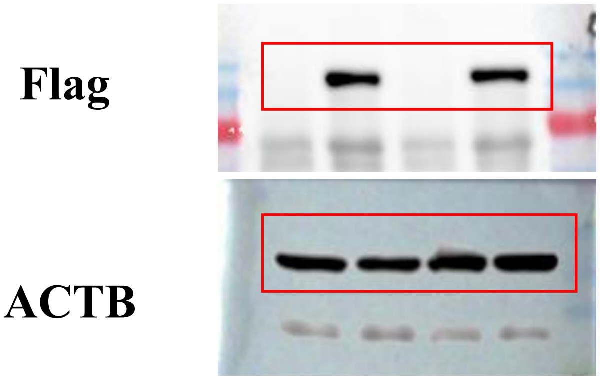

Figure S4B

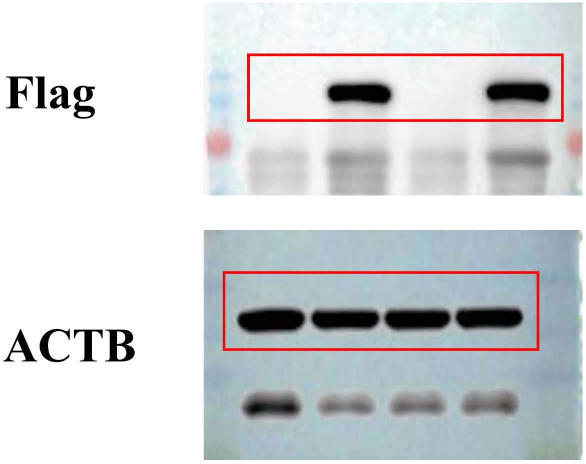

Figure S4K

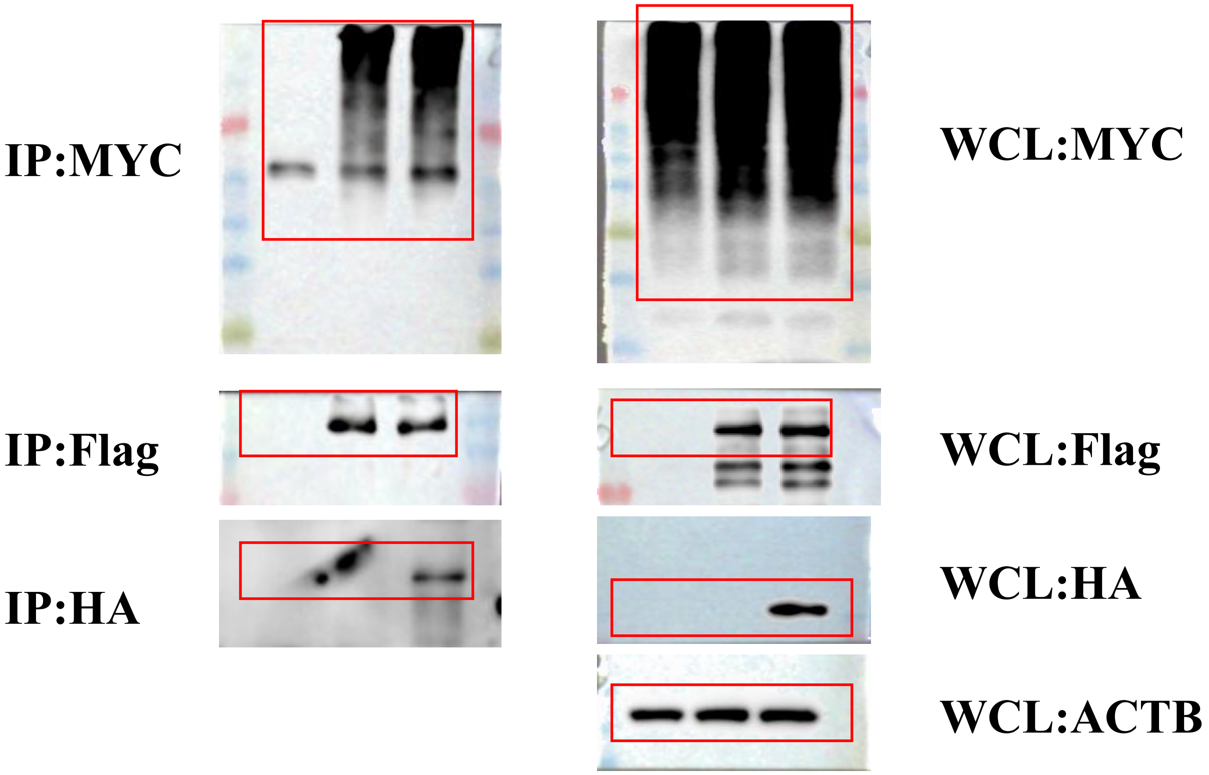

Figure S4L

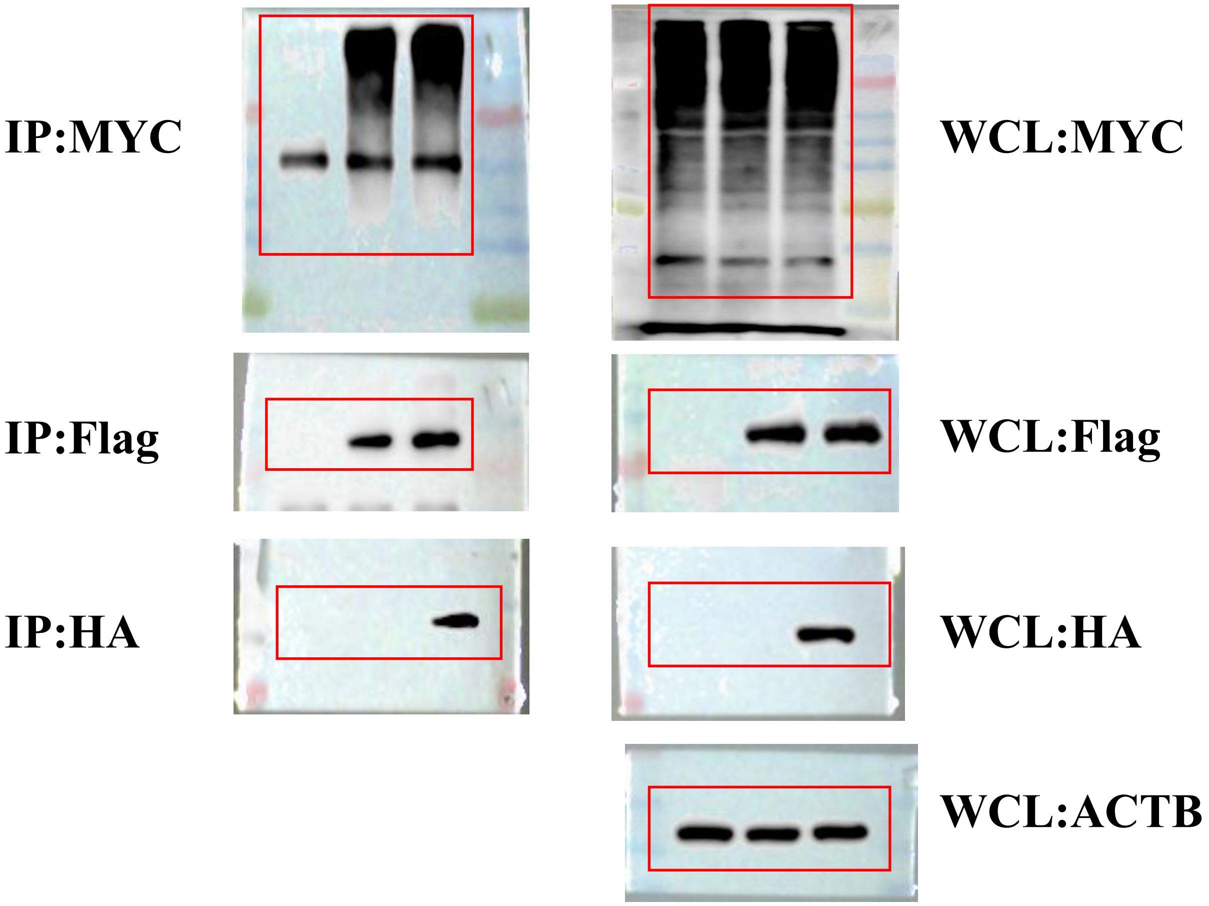

Figure S5A Figure S5B Figure S5C

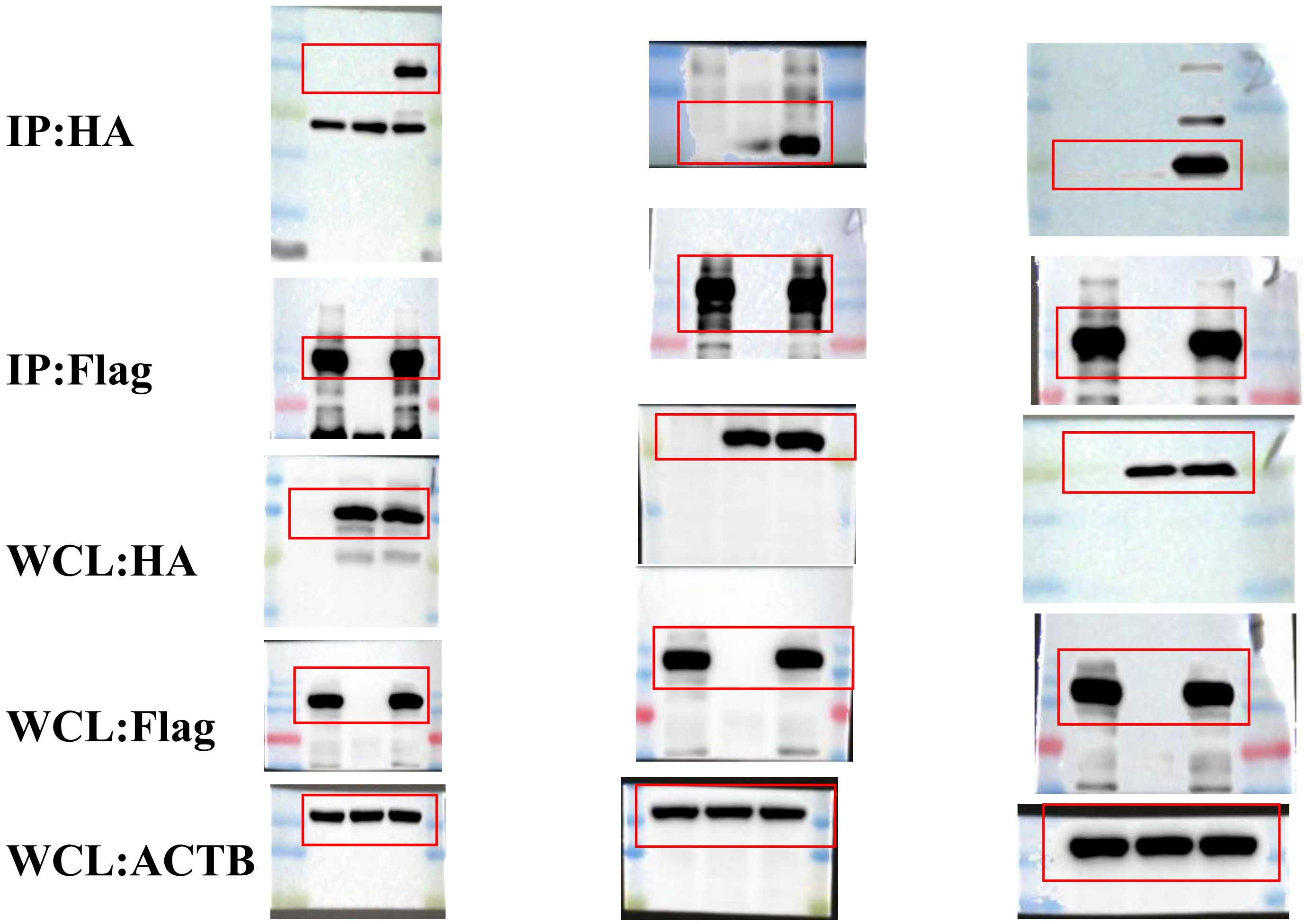

Figure S5D Figure S5E Figure S5F

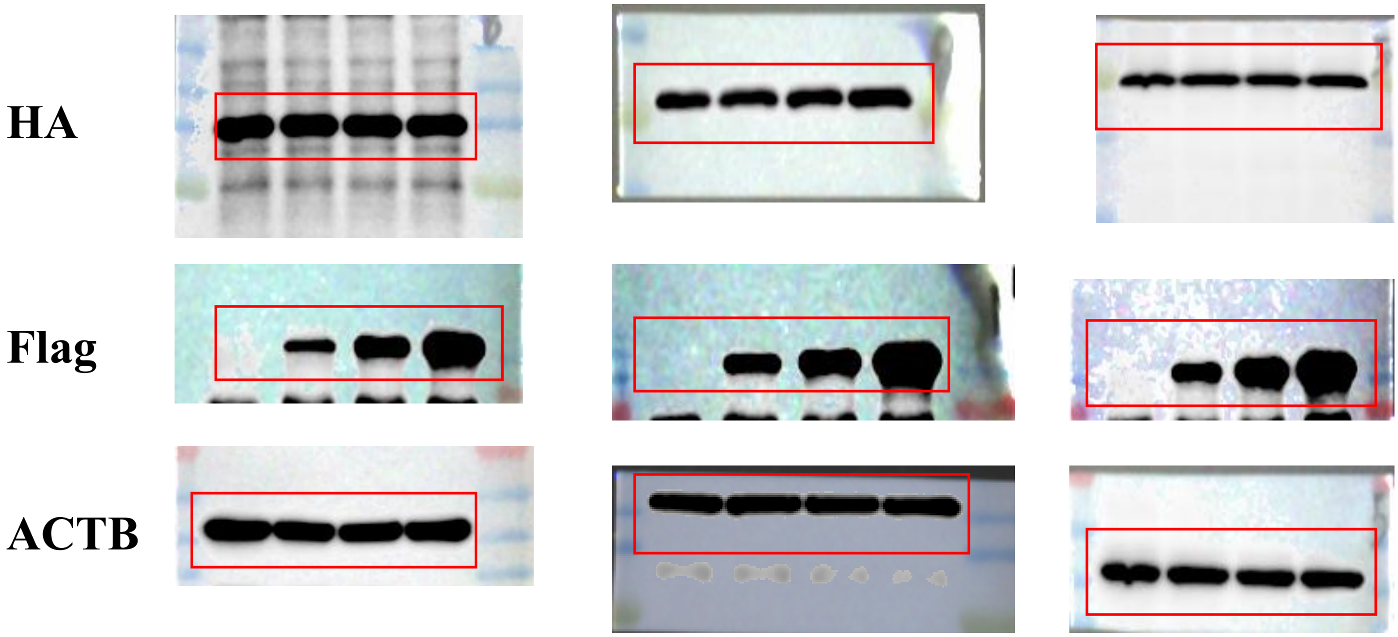

Figure S5G

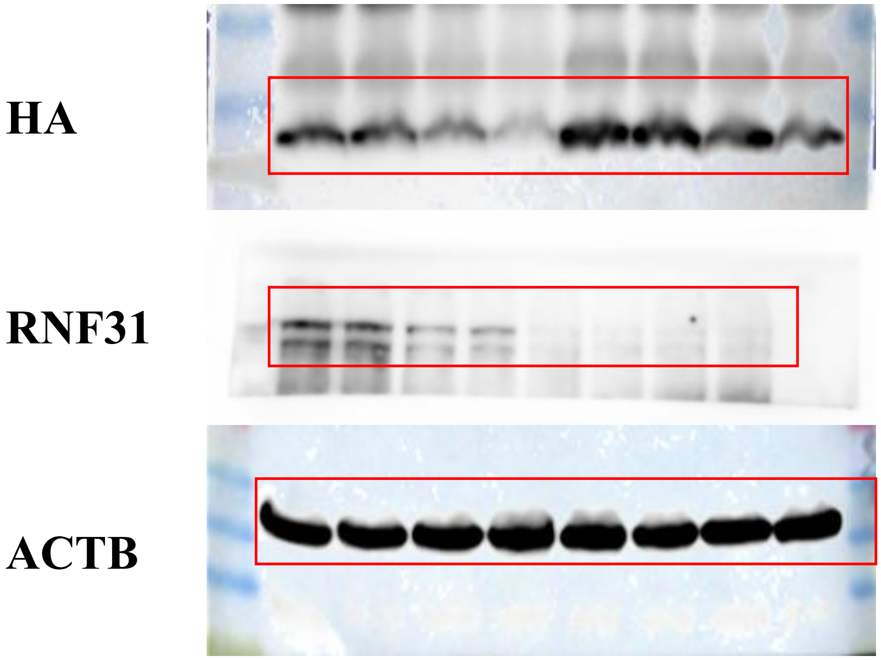

Figure S5I

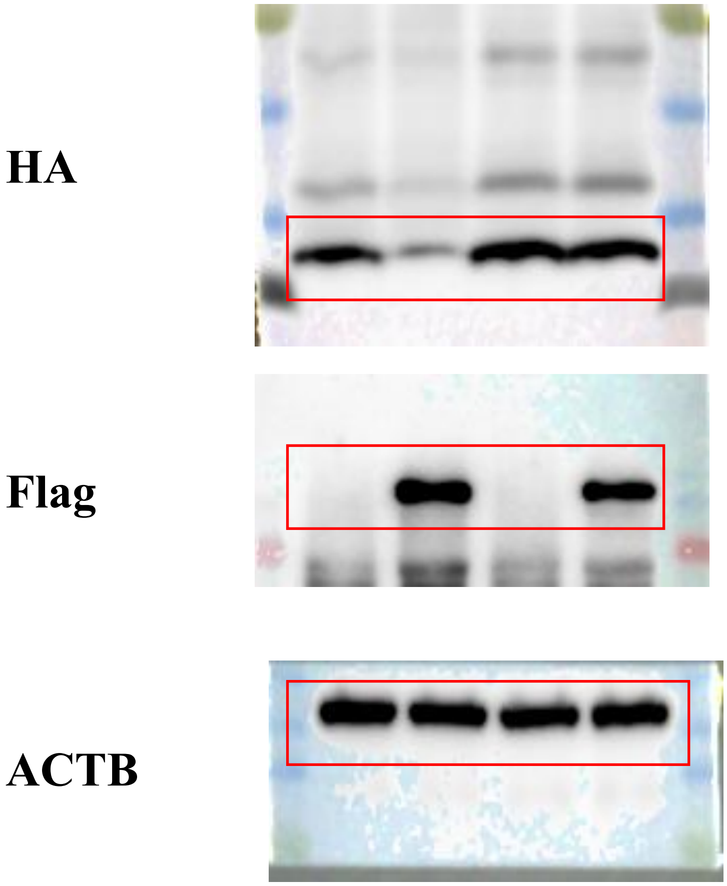

Figure S6A

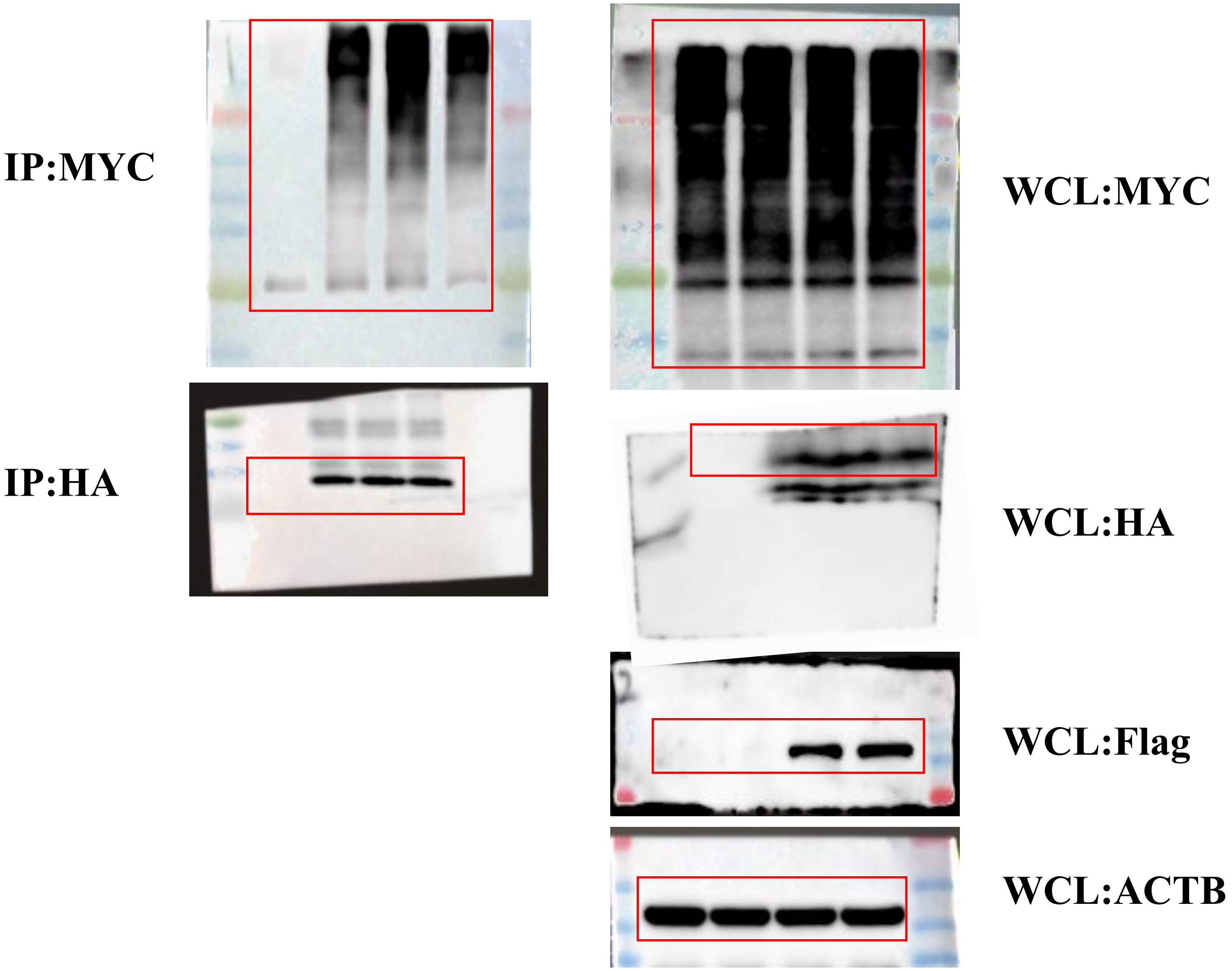

Figure S6B

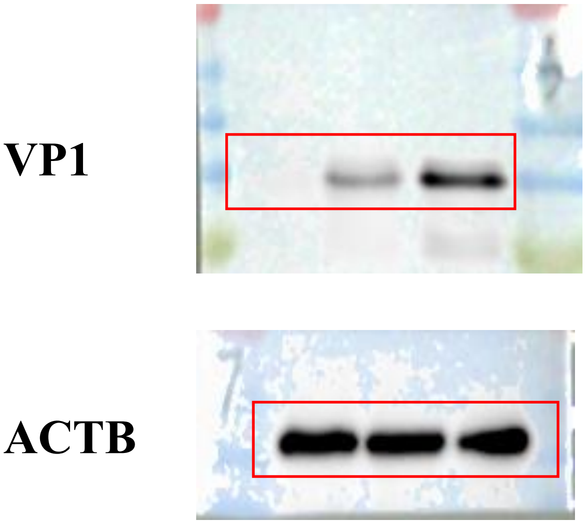

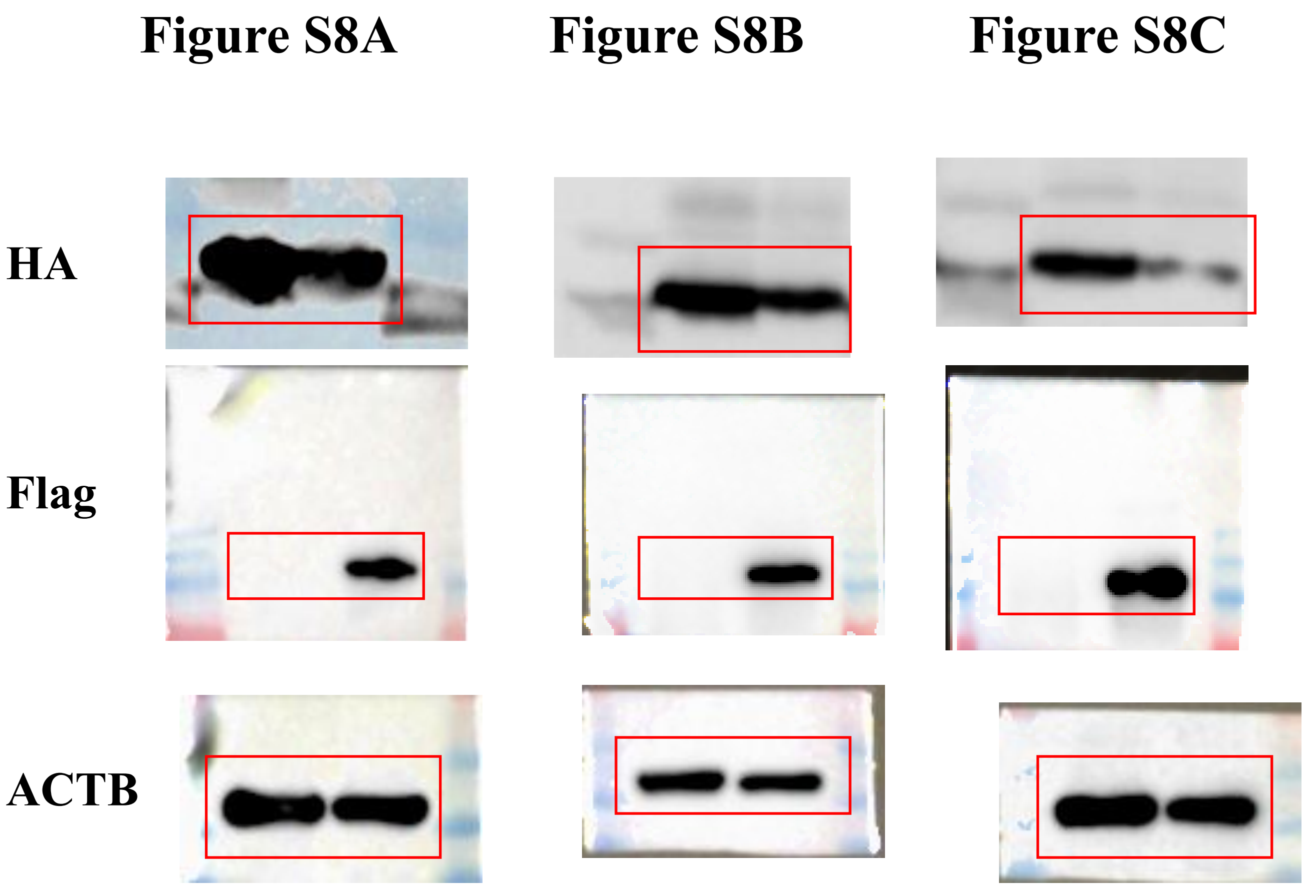
Figure S8A
Figure S8B
Figure S8C
HA
Flag
ACTB
